# Hidden Hazards: Assessment of Exposure Risks from 3-Monochloropropane-1,2-diol Ester (3-MCPDE) and Glycidyl Ester (GE) Consumption Among Malaysian Consumers

**DOI:** 10.3390/toxics14040331

**Published:** 2026-04-16

**Authors:** Siti Hajar Muhamad Rosli, Nurul Izzah Ahmad, Nurul Hidayati Surawi, Rohana Ani, Nor Kamilah Mohamad Alwi, Ami Fazlin Syed Mohamed

**Affiliations:** 1 Herbal Medicine Research Centre, Institute for Medical Research, National Institutes of Health, Ministry of Health Malaysia, Setia Alam 40170, Selangor, Malaysia; 2 Infectious Disease Research Centre, Institute for Medical Research, National Institutes of Health, Ministry of Health Malaysia, Setia Alam 40170, Selangor, Malaysia; nizzah.a@moh.gov.my; 3 Food Safety and Quality Programme, Ministry of Health Malaysia, Wilayah Persekutuan Putrajaya 62675, Malaysia; 4 Institute for Medical Research, National Institutes of Health, Ministry of Health Malaysia, Setia Alam 40170, Selangor, Malaysia; ami@moh.gov.my

**Keywords:** 3-MCPD, glycidyl ester, risk assessment, dietary exposure, cancer risk

## Abstract

3-Monochloropropanediol esters (3-MCPDEs) and glycidyl esters (GEs) are food processing contaminants that raise significant food safety concerns due to their established potential for carcinogenicity. This study aimed to determine the occurrence of 3-MCPDEs and GEs in common Malaysian food items and to evaluate the associated health risks through dietary exposure assessment. A total of 251 samples, consisting of retail products and cooked/prepared meals, were analysed using GC-MS. The food consumption data were obtained from published national food surveys. Risk was characterised using health-based guidance values (HBGVs) and margin of exposure (MOE), lifetime cancer risk (LCR), and disability-adjusted life year (DALY) estimates. 3-MCPDE was detected in 94.8% of samples (range: ND to 7.77 mg/kg), while GE was found in 83.3% of samples (range: ND to 9.41 mg/kg). The highest levels were consistently observed in refined vegetable fats and oil products, specifically shortening (3-MCPDE: 3.53 [IQR 2.76–5.16] mg/kg; GE: 4.78 [IQR 3.52–6.14] mg/kg) and margarine (3-MCPDE: 2.50 [IQR 1.11–3.59] mg/kg; GE: 3.60 [IQR 1.18–5.26] mg/kg). Exposure assessment identified fried rice as the largest contributor to total daily intake (3-MCPDE: 3.16 μg/kg BW/day; GE: 1.36 μg/kg BW/day). Total exposure to 3-MCPDE exceeded the provisional maximum tolerable daily intake (PMTDI) established by JECFA by 39.5%, indicating a potential health concern. Low MOE estimates (<10,000) for 3-MCPDE and GE were determined for several food categories, including snacks, kuih-muih, and fried cooked dishes. Chronic GE exposure was estimated to cause up to 6.9 (for mean consumers) and 24.9 (for high consumers) cancer cases per year, with total the DALYs quantified at 124.2 years lost per 100,000 of the population. These data represent a worst-case scenario; however, risks could be minimised through continued surveillance, mitigation strategies by relevant authorities regarding food processing, and informed dietary choices.

## 1. Introduction

Food safety concerns regarding the presence of 3-monochloropropane-1,2-diol esters (3-MCPDEs) and glycidyl esters (GEs) in everyday food products have become a persistent public health issue due to their potential health risks and toxicological profiles [1]. The formation of these contaminants is closely linked to the deodorisation step in vegetable oil refining, which occurs under high-temperature conditions, particularly those exceeding 200 °C [2].

Upon ingestion, 3-MCPDEs are hydrolysed in the gastrointestinal tract to release 3-MCPD, which has been classified by the International Agency for Research on Cancer (IARC) as a possible human carcinogen (Group 2B). Similarly, GEs are hydrolysed to glycidol, a compound recognised as a genotoxic carcinogen and classified under Group 2A [3]. Glycidol has been characterised with an oral cancer slope factor of 1.3 mg/kg per day [4], derived from the dose–response relationship for multiple tumour sites in rats exposed to glycidol orally. The types of cancers included in the final cancer potency assessment include brain glioma, forestomach carcinoma, mammary gland fibroadenoma, and thyroid gland follicular cell adenoma/carcinoma, among others [5]. Given the widespread use of refined vegetable oils in various food products, ranging from infant formulas to confectionery, ensuring these contaminants are kept at minimal levels is crucial.

Recognising the risks, regulatory bodies such as the European Union (EU) and Codex Alimentarius have established maximum permissible limits for these contaminants and have issued guidelines for their reduction in food products [6]. This has led to comprehensive exposure assessments and subsequent regulatory measures aimed at reducing the levels of 3-MCPDE and GE in the food supply. Current regulations set by the European Commission (EC) prescribe maximum levels of 2.5 mg/kg for 3-MCPD and 1.0 mg/kg for GE for vegetable oils and fats used as ingredients in food [6]. The Joint Food and Agriculture Organisation/World Health Organisation (FAO/WHO) Expert Committee on Food Additives (JECFA) established a group provisional maximum tolerable daily intake (PMTDI) of 4 μg/kg body weight for 3-MCPD and 3-MCPDEs, singly or in combination (expressed as 3-MCPD equivalents) [7]. Due to the genotoxic and carcinogenic nature of glycidol, no safety reference threshold can be established for GE; instead, the principle of “As Low as Reasonably Achievable” (ALARA) applies.

Numerous studies have documented that palm oil and its derivatives are especially prone to high GE levels due to their intrinsic diacylglycerol (DAG) content [1,8]. As one of the world’s largest producers and exporters of palm oil, Malaysia faces significant economic pressure to manage these contaminants. As palm oil is considered a staple in the Malaysian diet, with palm oil-rich products commonly providing the main fat source of many dishes, the presence of these contaminants has been considered a potential health risk. To our knowledge, no health risk assessment study focusing on these compounds in cooked/prepared meals has been conducted in Malaysia to date. The current article extends the findings of our previous publication regarding the association of 3-MCPDE intake with the development of renal cancer [9]. In this study, we aim to (i) determine the occurrence levels of 3-MCPDE and GE in common retail and cooked/prepared foods in Malaysia, (ii) comprehensively assess the dietary exposure risks to these contaminants among the Malaysian adult population, and (iii) characterise the probable disease burden of cancers associated with chronic consumption and their impact on public health.

## 2. Materials and Methods

### 2.1. 3-MCPDE and GE Occurrence Levels in Food

#### 2.1.1. Selection and Sampling of Food Items

**Figure 1:**
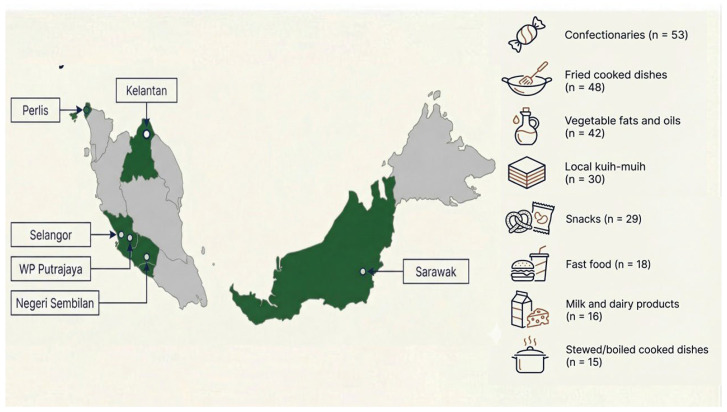
Geographical distribution of the six Malaysian territories selected for sampling of food items from eight food categories comprising retail and cooked/prepared foods (*N* = 251). These territories were selected based on a risk-based prioritisation approach to optimise budgetary and analytical resources. These diverse locations represent a mix of rural and urban settings with varied agricultural and dietary patterns, providing a representative cross-section for exposure assessment.

Between November 2017 and December 2018, food samples were collected using two distinct strategies. For prepared foods, a minimum of three freshly cooked samples were purchased at random from various restaurants and stalls. For processed retail foods, market surveys identified top national brands by sales volume, where 1 kg of each brand was then sourced from major grocery chains across the states of Selangor, Negeri Sembilan, Perlis, Kelantan, Sarawak, and the Federal Territory of Putrajaya (Figure 1). Food items were selected for sampling based on those that were high in the data collection priorities outlined in the EFSA open call for data (October 2014), specifically targeting vegetable oils and fats, bakery wares, bread and rolls, and potato- or cereal-based snacks [10]. This list was tailored to the Malaysian diet, taking into account consumption data available in the Malaysian Adult Nutrition Survey (MANS) 2014 [11]. All selected food items were known to contain significant amounts of vegetable oil, either as an ingredient or due to preparation techniques (e.g., frying, grilling). The most common vegetable oils identified in the samples were palm oil (palm olein), palm kernel oil, sunflower oil, and canola oil. The food samples were grouped into eight categories: vegetable fats and oils, milk and dairy products, confectioneries, snacks, fast food, local *kuih-muih* (Malaysian sweet or savoury bite-sized desserts), fried cooked dishes, and stewed/boiled cooked dishes.

#### 2.1.2. Determination of 3-MCPDE and GE in Food Samples Using GC-MS

The analysis was conducted by two separate accredited laboratories, each processing samples collected from different states. The standard method Cd 29a-13 for 3-MCPD quantification in edible oils and fats, developed by the American Oil Chemists’ Society (AOCS), was employed [12]. Analysis was performed using an indirect method where oil-based samples (0.5 g) were extracted with n-heptane/MTBE (1:2 *v*/*v*) and enriched with internal standards. Glycidyl esters were converted to 3-monobromopropanediol (3-MBPD) monoesters via acidic sodium bromide, followed by acid transesterification of all analytes into free forms at 40 °C for 16 h. The released diols were derivatised with phenylboronic acid and analysed on a GC-MSD system (Agilent Technologies, Santa Clara, CA, USA) equipped with a DB-5ms capillary column. Helium served as the carrier gas at 0.8 mL/min, with a 2 μL pulsed splitless injection at 250 °C. The oven temperature was programmed from 80 °C to 300 °C using a multi-step ramp to ensure optimal separation. Detection was conducted in electron impact (EI) mode using selected ion monitoring (SIM), with quantification based on *m*/*z* 147 (3-MCPD) and *m*/*z* 150 (d_5-3-MCPD). Detailed specifications are available as Supplementary File S1.1. All quality assurance and quality control parameters were assessed in accordance with guidelines, norms, and accredited procedures as practised by the Department of Chemistry, Malaysia.

The content of bound 3-MCPDE is reported in milligrams per kilogram (mg/kg). Analytical results below the limits of detection (LOD) and limit of quantification (LOQ) were treated following recommendations by EFSA [13]. As the proportion of non-quantified values was less than 60% of the overall data, we utilised the upper-bound (UB) approach, whereby all non-detectable (ND) results were set equal to the LOD, and all non-quantified results were set equal to the LOQ. This conservative approach ensures a worst-case scenario estimate, thereby minimising the potential underestimation of dietary exposure. For the calculation of mean and median concentrations of 3-MCPDE and GE in food samples, a health-protective upper-bound estimate was adopted in line with international risk assessment practices.

### 2.2. Food Consumption and Dietary Exposure Assessment

Consumption data for the total Malaysian population were obtained from the *Food Consumption Statistics Report* of the MANS conducted in 2014 [11]. This report, produced and published by the Institute for Public Health, Ministry of Health Malaysia, provides a comprehensive nationwide consumption database for Peninsular Malaysia and East Malaysia (Sabah and Sarawak). The survey listed 165 common foods in Malaysia, providing the estimated mean intake of each food in grams per day, the standard error of the mean, the estimated population, prevalence (%), and median and percentile consumption values among the adult population. Analyses were conducted for the total Malaysian population and stratified by sex (male, female) and race/ethnicity (Malay, Chinese, Indian, Others (*Bumiputera*), and Others (minorities)). The “Others (*Bumiputera*)” category comprises indigenous *Orang Asli* and ethnic groups from Sabah and Sarawak, while “Others (minorities)” includes smaller ethnic groups and non-native populations residing in Malaysia (e.g., Sikhs, Serani/Eurasians, Portuguese-descended groups, Indonesians, and Bangladeshis).

The daily dietary exposure to 3-MCPDE and GE for individual food items was estimated by multiplying the occurrence level by the consumption data for each item, divided by the average body weight (BW) (Equation (1)). BW values were obtained from the MANS report Nutritional Status of Adults Aged 18–59 Years [14]. The overall mean BW for the total population was 62.65 kg, while the means for males and females were 66.56 kg and 58.44 kg, respectively.

Calculation of dietary exposure for each food item and food category is as follows:

(1) $${\mathrm{EDE}}^{i}\;=\;\frac{{IR}^{i}\;\times \;C^{i}\;}{\mathrm{Average}\;\mathrm{BW}\;(\mathrm{kg})}$$

EDE^i^: Estimated dietary exposure for each food item (mg/kg BW/day);
IR^i^: Intake rate for each food item (kg/day);
C^i^: Concentration level of contaminant for each food item (mg/kg).

To assess dietary exposure across food categories, a representative concentration value was calculated for each of the eight major food groups using a consumption-weighted average. This approach integrates the distribution-specific representative concentrations from all constituent food types, weighted by their respective average daily consumption rates (Equation (2)).

Calculation of the representative concentration value for each food category is as follows:

(2) $$C^{\mathrm{category}}\;=\;\frac{\;\sum {(IR}^{i}\;\times {\;C}^{i})\;}{\sum {IR}^{i}}\;$$

$${\mathrm{EDE}}^{\mathrm{category}}=\frac{\sum \left({IR}^{i}\;\times \;C^{i}\right)}{\mathrm{Average}\;\mathrm{BW}\;(\mathrm{kg})}$$

C^category^: Representative consumption-weighted average concentration (mg/kg);
EDE^category^: Estimated dietary exposure for each food category (mg/kg BW/day).

As this study focuses on specific food items containing palm oil, certain foods were not explicitly listed in the MANS. In such cases, food mapping was performed by identifying the most similar food type to obtain a representative consumption value. For instance, potato chips and flavoured snacks were grouped as “snacks,” and various local *kuih-muih* were collectively categorised as *kuih-muih* in the MANS (Supplementary File S1). For cooked/prepared meals, the main ingredient of the dish was used to represent the consumption rate. For example, *chicken soto*, composed primarily of compressed rice cake cubes, was assumed to have a consumption rate similar to that of white rice. Consequently, for these mapped items, a representative concentration value was derived by calculating the arithmetic mean of the concentrations of the constituent food items (Supplementary File S2). This method assumes an equal contribution of each item to the total consumption of that food type, ensuring a more accurate estimation of the consumption-weighted average concentration for each category.

For the intake assessment, the mean and median concentrations were linked to individual-level adult consumption quantities. Both the mean and the 95th percentile (p95) of the intake were considered for each food group. The mean concentration represents the average consumer, while the variation in intake accounts for those who may consume more or less of the contaminated food. The average intake reflects “mean consumers”, whereas the 95th percentile reflects “high consumers”.

### 2.3. Risk Characterisation of 3-MCPDE and GE Exposure

#### 2.3.1. Exposure Assessment Relative to Health-Based Guidance Value (HBGV)

For 3-MCPDE, the estimated exposure was compared to the PMTDI value of 4 µg/kg BW/day, established to protect against renal tubular hyperplasia [7]. Values are reported as a percentage of the PMTDI to identify food groups that exceed this reference value, thereby indicating potential toxicity risks. As no HBGV has been established for GE, we identified the food groups contributing the highest percentage to the total dietary exposure of GE for comparison.

#### 2.3.2. Margin of Exposure (MOE) Assessment

Given that genotoxic and carcinogenic compounds such as glycidol lack a safe threshold, risk was assessed using the MOE approach. The MOE is defined as the ratio of the no-observed-adverse-effect level (NOAEL) derived from animal toxicology studies to the predicted or estimated human exposure level (Equation (3)). This method is adopted as an alternative to low-dose extrapolation to provide a reference point for risk concern regarding genotoxic and carcinogenic chemicals [15]. This approach facilitated the identification of food groups in the Malaysian diet containing high levels of contaminants that may exhibit such hazards. The adaptation of this method follows the guidelines proposed by Edler et al. (2014) regarding the relevance of animal tumour data for human carcinogenic hazard assessment [16].

Calculation of risk characterisation using MOE:

(3) $$\mathrm{MOE}\;=\;\frac{{\mathrm{BMDL}}_{10}\;}{\mathrm{EDI}}$$

MOE: Margin of exposure;
BMDL_10_: Benchmark dose, lowest 10%;
EDI: Estimated daily intake (mg/day).

MOE is expressed as the ratio between an appropriate Point of Departure (POD) on the dose–response curve for tumour response and a relevant estimate of human exposure. In in vivo studies, the POD or reference point used for calculating the MOE is typically the lower 95% confidence limit (BMDL) of the benchmark dose (BMD) that produces a specified response (Benchmark Response or BMR). The BMDL is used to establish a POD that assures high (95%) confidence that the specified response will not be exceeded at that dose level [17].

In this study, a BMDL_10_ value of 0.20 mg/kg BW per day for 3-MCPDE was selected as the reference point for evaluating potential renal effects in humans [18]. Based on experimental evidence of substantial GE hydrolysis to glycidol in the gastrointestinal tract, the JECFA evaluation presumes that GEs hydrolyse completely and exert their toxicity through glycidol within the body [19]. Consequently, for GE, the MOE was calculated using a BMDL_10_ value of 2.4 mg/kg BW/day as the reference point [19]. Risk assessors prioritise mitigation strategies for compounds with low MOE values (<10,000), whereas high MOE values (exceeding 10,000) are considered of low concern for genotoxic or carcinogenic risk [7].

#### 2.3.3. Estimation of Lifetime Cancer Risk (LCR) from Glycidol Exposure

In parallel, the excess LCR was estimated using the cancer slope factor (CSF) approach, where exposure is calculated using the lifetime average daily dose (LADD) (Equation (4)). In this study, the calculated dietary exposure value represents the LADD for chronic exposure. The U.S. Environmental Protection Agency (EPA) defines the CSF for glycidol as approximately 1.3 (mg/kg BW/day)^−1^ [4]. This factor represents the increased risk of cancer resulting from oral exposure to the chemical. The obtained LADD value is then multiplied by the chemical-specific CSF to estimate the lifetime cancer risk (LCR) for each food item (Equation (5)) and across all food categories (Equation (6)). To estimate the mean annual carcinogenic risk (ACR), the LCR was divided by the life expectancy (LE) (Equation (7)).

Calculation of exposure using LADD:

(4) $$\mathrm{LADD}\;=\frac{C\;\times \;\mathrm{IR}\;\times \;\mathrm{EF}\;\times \;\mathrm{ED}}{\mathrm{BW}\;\times \;\mathrm{LT}}\;$$

C: GE concentration in food (mg/kg);
IR: Intake rate (kg/day);
EF: Exposure frequency (days/year);
ED: Exposure duration (years);
BW: Body weight (kg);
LT: Lifetime exposure in days (life expectancy years × 365).

Calculation of excess LCR (per food item):

(5) $${\mathrm{LCR}}^{i}\;=\;{\mathrm{LADD}}^{i}\times \;{\mathrm{CSF}}^{i}$$

Calculation of aggregate LCR (across all food items):

(6) $${\mathrm{LCR}}^{\mathrm{total}}\;=\;\sum \left({\mathrm{LADD}}^{i}\times \;{\mathrm{CSF}}^{i}\right)$$

Calculation of the mean ACR:

(7) $$\mathrm{ACR}\;=\;\frac{{\mathrm{LCR}}^{\mathrm{total}}}{\mathrm{LE}}\;$$

LCR^i^: Lifetime cancer risk (per food item);
LCR^total^: Total lifetime cancer risk (all food items);
CSF^i^: Cancer slope factor (per food item);
ACR: Annual cancer risk;
LE: Life expectancy (total population: 75.2 years; males: 73 years; females: 77.8 years) [20].

The calculated LCR and ACR from dietary exposure to glycidol were determined for various food categories using a standardised risk assessment model, considering both the mean and high consumption patterns. A No Significant Risk Level (NSRL) of 0.54 µg/day, derived by the California Office of Environmental Health Hazard Assessment [21] was used as the reference point. This value corresponds to a lifetime cancer risk of 1 in 100,000.

#### 2.3.4. Estimating the Burden of Disease from Glycidol-Related Cancers Using the Disability-Adjusted Life Year (DALY) Approach

In risk assessment, the DALY is a crucial metric for quantifying the overall disease burden, representing the number of healthy years of life lost due to morbidity, disability, and premature mortality [22]. One DALY unit is equivalent to one lost year of healthy life. As there are no separate coefficients for each cancer site associated with glycidol, we employed an indirect method. This involved multiplying the total burden of disease (in DALYs) for all cancers in Malaysia (excluding non-melanoma skin cancer) by the fraction of cancer cases attributable to glycidol (Equation (8)). The total cancer incidence data were obtained from the 2022 Global Cancer Observatory (GLOBOCAN) census [23], while age-standardised DALY values for total cancers in Malaysia were retrieved from the Global Burden of Disease (GBD) online interactive tool (2021 report) [24]. Results are expressed as DALYs per 100,000 population, with the 95% Uncertainty Interval (UI) shown.

Calculation of DALY estimate:

(8) $$\mathrm{FGR}\;=\;\frac{\mathrm{ACR}}{{\mathrm{INC}}^{\mathrm{total}}}\;$$

$${\mathrm{DALY}}^{\mathrm{glycidol}}=\mathrm{FGR}\;\times {\;\mathrm{DALY}}^{\mathrm{total}\;\mathrm{cancers}}$$

FGR: Fraction of glycidol-related cancers;
ACR: Annual cancer risk from glycidol exposure;
INC^total^: Incidence of total cancers in Malaysia for the year 2022 (total population 2697.9, males: 2684.5, females: 2737.7) [23];
DALY^total cancers^: Age-standardised DALYs estimated for total cancers in Malaysia for the year 2021 (2710.2 per 100,000 population) [24].

### 2.4. Data Analysis

Prior to analysis, data cleaning and validation were performed to ensure analytical rigour. Initial screening involved a check for transcriptional errors and consistency across technical replicates. A substitution method was employed, wherein the ND results were assigned equal to the LOD, and the non-quantified results equal to the LOQ. Normality was assessed using the Kolmogorov–Smirnov and Shapiro–Wilk tests, which indicated a non-normal distribution. Consequently, non-parametric methods were employed for all descriptive and inferential statistics. Outliers were cross-referenced with laboratory notes to rule out experimental error before being included in the final dataset to maintain relevance. Occurrence levels were reported as medians, interquartile ranges (IQRs), and percentile ranges for each food item. Calculations for dietary exposure, risk characterisation (MOE), cancer risk (LADD, LCR, ACR, FGR), and burden of disease (DALY) were performed using Microsoft Excel 2024 (Microsoft Corp., Redmond, Washington, DC, USA) and SPSS (version 26, IBM Software Group, Chicago, IL, USA). Differences in occurrence levels, dietary exposure, and LCR for GE between food groups were assessed using the Kruskal–Wallis (KW) test for three or more groups and the Mann–Whitney (MW) U test for pairwise comparisons. The significance level was set at *p* < 0.05. All figures and graphical illustrations were generated using GraphPad Prism software 10.5 (GraphPad Software Inc., San Diego, CA, USA).

## 3. Results

### 3.1. 3-MCPDE and GE Occurrence Levels in Malaysian Foods

In total, 251 samples across eight categories comprising 58 different food items were analysed. The occurrence levels of 3-MCPDEs and GEs, categorised by food group, are summarised in Table 1. The detected levels of both compounds fell within the working ranges of 0.3–9.3 μg/g for 3-MCPDE and 0.6–21.3 μg/g for GE. Final results are reported as the concentration of 3-MCPDEs and GEs in the sample, expressed on a food weight basis in milligrams per kilogram (mg/kg). For Laboratory A (which analysed cooked/prepared food samples), the LOD and LOQ were 0.11 mg/kg and 0.16 mg/kg for 3-MCPDE, and 0.04 mg/kg and 0.31 mg/kg for GE, respectively. For Laboratory B (which analysed retail food samples), the LOD and LOQ were 0.07 mg/kg and 0.21 mg/kg for 3-MCPDE, and 0.09 mg/kg and 0.27 mg/kg for GE, respectively.

As 3-MCPDE contamination has been detailed in our previous publication [10], this section emphasises GE occurrence. GE was detected in 209 (83.3%) of the sampled food items. Of the total samples analysed, 134 (53.4%) showed analyte levels exceeding the LOQ, allowing for accurate quantification. Broken down by food category, quantifiable levels were found in 25 samples of vegetable fats and oils (59.5% of n = 42), 27 local *kuih-muih* (90.0% of n = 30), 21 fried cooked foods (39.6% of n = 48), four stewed cooked foods (26.7% of n = 15), 22 snacks (75.9% of n = 29), three fast food items (16.6% of n = 18), five milk and dairy items (31.3% of n = 16), and 27 confectioneries (50.9% of n = 53). The remaining 123 (49%) samples contained detectable residues below the LOQ, while 42 (16.7%) were below the LOD (non-detects).

Overall, individual occurrence levels of GE exhibited wide variability across all analysed samples, ranging from ND to 9.41 mg/kg (wet weight), with a median of 0.34 (IQR: 0.27–0.94) mg/kg. A total of 59 (23.5%) samples exceeded the permissible level of 1.0 mg/kg. The highest levels were consistently found in shortening (median 4.78 [IQR 3.52–6.14] mg/kg), followed by margarine (median 3.60 [IQR 1.18–5.26] mg/kg). Among snacks, the highest levels were detected in seafood-flavoured snacks (median 1.55 [IQR 0.92–1.69] mg/kg) and *murukku* (median 0.92 [IQR 0.57–3.68] mg/kg). For confectionery, high levels were observed in plain biscuits (median 1.25 [IQR 0.52–1.25] mg/kg). In the cooked/prepared foods category, GE levels were notably high in fried butter prawns (median 1.30 [IQR 0.34–0.47] mg/kg) and fried anchovies (median 1.22 [IQR 1.18–5.26] mg/kg). Finally, among the *kuih-muih*, levels above 1.0 mg/kg were observed in the fried *cempedak* and curry puff samples (median: 1.17 mg/kg).

**Figure 2:**
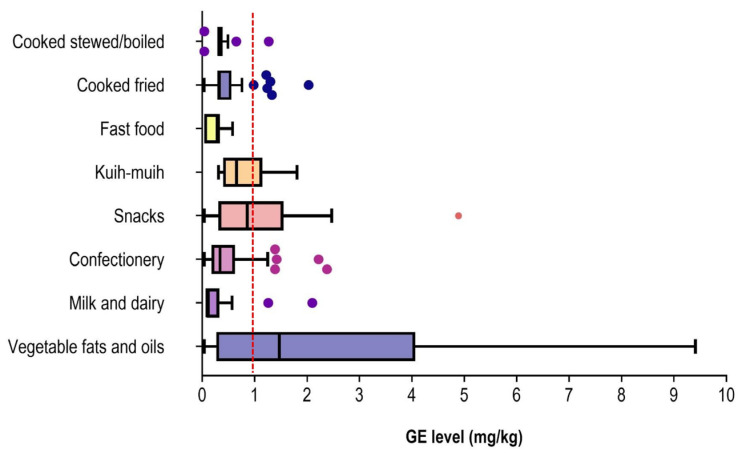
GE occurrence level (mg/kg) according to food category. Box plots represent the median, IQR, minimum and maximum values of each food sample analysed. Low outlier values were detected in chicken soto (n = 2) within the cooked stewed/boiled category. High outlier values were detected in fried anchovies (n = 2) and butter prawn (n = 3) for the cooked fried category; flavoured biscuits (n = 4) and plain biscuits (n = 1) for confectioneries; *murukku* (n = 1) from snacks; and butter (n = 2) from milk and dairy. The red vertical line indicates the maximum permissible limit of 1.0 mg/kg (JECFA).

The difference in GE levels between the eight food groups was statistically significant (ꭓ^2KW^ = 42.13; *p* = 0.001) (Figure 2). Category-wise, vegetable fats and oils recorded the highest median values of 1.47 (IQR 0.10–4.05) mg/kg, significantly higher than fast food and milk/dairy products (*p* < 0.05). Among the cooked/prepared foods, the highest overall levels were detected in the *kuih-muih* category. *Kuih-muih* (median 0.62 [IQR 0.40–1.09] mg/kg) had significantly higher levels than confectionery (median 0.35 [IQR 0.11–0.61] mg/kg) and fried cooked foods (median 0.31 [IQR 0.09–0.51] mg/kg). The lowest values were recorded in the fast-food group, where 82% of samples were non-quantified. Despite the higher potential for exposure from vegetable oils, pairwise comparison showed GE levels in fried cooked food did not differ significantly when compared to stewed or boiled food samples.

### 3.2. Dietary Exposure to 3-MCPDE and GE for the Malaysian Population

Dietary exposure to 3-MCPDE and GE was assessed across various food groups for the total adult population, with further stratification by sex (males, females) and ethnicity (Malay, Chinese, Indian, Others (*Bumiputera*), and Others (minorities)). For the general population, the total dietary exposure to 3-MCPDE, considering all food categories, was 5.58 µg/kg BW/day (range: 0.01–4.37 µg/kg BW/day) for the mean consumers. For high consumers (p95), exposure reached up to 18.98 µg/kg BW/day (range: 0.08–13.53 µg/kg BW/day). Similarly, total GE exposure was 5.52 µg/kg BW/day (range: 0.01–2.50 µg/kg BW/day) for mean consumers and 18.73 µg/kg BW/day (range: 0.08–7.99 µg/kg BW/day) for high consumers (Table 2). Detailed results are provided in Supplementary File S2.

#### 3.2.1. Exposure Comparison Across Food Categories and Subpopulations

The difference in dietary exposure levels across food groups showed marginal significance for both 3-MCPDE (ꭓ^2KW^: 13.82, *p* = 0.06) and GE (ꭓ^2KW^: 12.86, *p* = 0.07) for the total population. However, both compounds showed similar trends, with higher exposure ranks observed for cooked fried, *kuih-muih*, and cooked stewed categories compared with milk and dairy or fast foods. Across all ethnic groups, the highest mean exposures resulted from the consumption of cooked fried foods. This category recorded 3-MCPDE exposures ranging from 3.60 µg/kg BW per day in Indians and 5.85 µg/kg BW/day in Others (*Bumiputera*). GE exposure ranged from 1.88 µg/kg BW/day in Indians and from 3.21 µg/kg BW/day in Others (*Bumiputera*).

**Figure 3:**
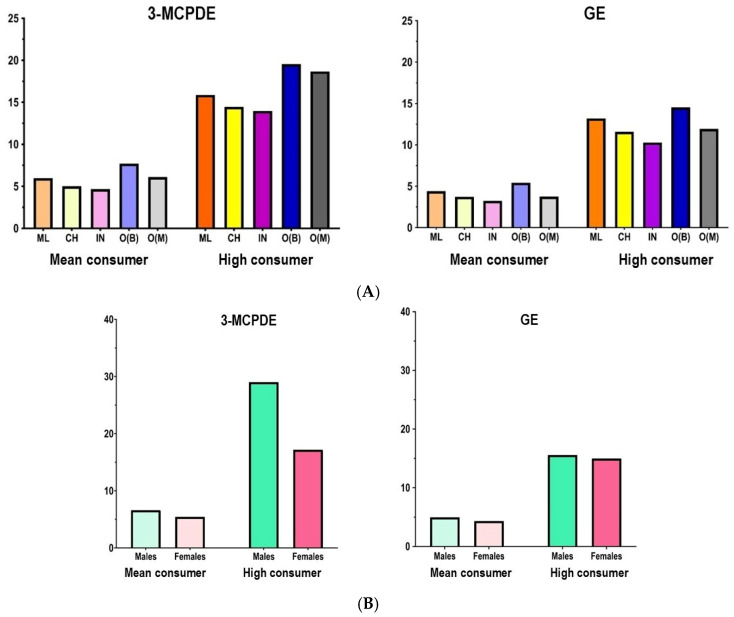
Total dietary exposure of 3-MCPDE and GE for mean and high consumers considering all food categories reported in µg/kg BW per day, comparing (**A**) between races and (**B**) between sexes (male/female). ML: Malay, CH: Chinese, IN: Indian, O(B): Others (*Bumiputera*)—indigenous *Orang Asli* and ethnic groups from Sabah and Sarawak, O(M): Others (minorities)—smaller ethnic and non-native populations in Malaysia (e.g., Sikhs, Serani/Eurasians, Portuguese-descended groups, Indonesians, Bangladeshis). Comparison of exposure between race groups: 3-MCPDE (ꭓ^2KW^: 8.91, *p* = 0.06) and GE (ꭓ^2KW^: 9.15, *p* =0.057).

Despite the Others (*Bumiputera)* group exhibiting the highest total cumulative exposure (3-MCPDE: 7.75 µg/kg BW/day, GE: 5.45 µg/kg BW/day) (Figure 3A), Malays recorded the highest overall mean rank. This disparity was attributable to the greater diversity of contaminated food vehicles within the Malay diet, leading to more frequent exposure across multiple food categories. Regarding sex, males were exposed to 0.01 to 4.85 µg/kg BW/day for 3-MCPDE and 0.001 to 2.98 µg/kg BW/day for GE. Exposure in females ranged from 0.01 to 3.88 µg/kg BW/day for 3-MCPDE and 0.00 to 2.40 µg/kg BW/day for GE (Figure 3B). Although there were no significant differences in exposure levels between males and females for either compound, the total cumulative exposure for males was found to be 19.5% higher for 3-MCPDE and 13.5% higher for GE compared to females. This observation is likely due to notable preferences among males for foods such as fried rice, concentrated creamers, and instant noodles.

#### 3.2.2. Exposure Relative to HBGV for 3-MCPDE

For the total population, mean consumers exceeded the risk threshold by 39.5% of the PMTDI. This was largely driven by cooked fried foods, which contributed 4.37 µg/kg BW/day to the total daily exposure. (109.2% of the PMTDI). Specifically, the consumption of fried rice alone accounted for 3.16 µg/kg BW per day (79% of the PMTDI) for mean consumers, with potentially elevated risks observed among high consumers, where exposure reached 8.27 µg/kg BW per day (206.9% of PMTDI). All other food items contributed less than 15% of the HBGV based on average intake. Dietary exposure was more pronounced in males, who exceeded the safety threshold by a wider margin (64.7%) than their female counterparts (35.6%). Among the ethnicities, the Others (*Bumiputera*) group was exposed to levels nearly double the safety threshold, with cumulative exposure reaching 7.75 µg/kg BW/day.

### 3.3. Risk Characterisation Through the MOE Estimate

**Figure 4:**
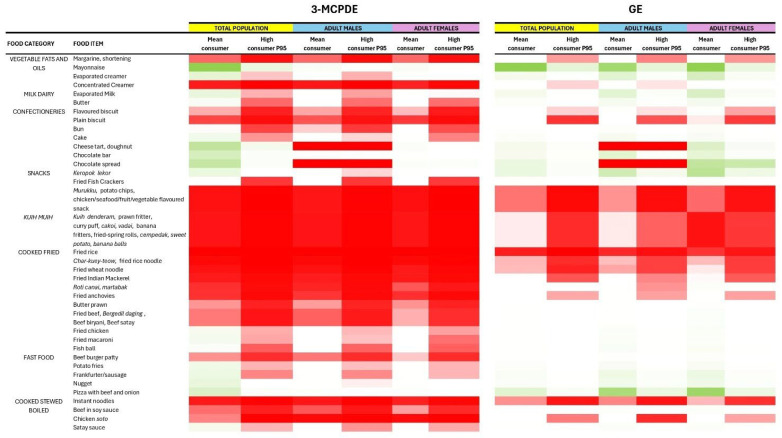
Heatmap of MOE estimate for 3-MCPDE and GE for the total population, male, and female subgroups (mean consumers and high consumers). Red colour: MOE < 10,000 indicating potential concern for toxicity risk. White and green colour: MOE > 10,000 indicating low concern for toxicity risk.

Food items posing a potential toxicity risk for 3-MCPDE and GE were identified and visualised in Figure 4. For mean consumers, MOE values for 3-MCPDE ranged from 63 to 182122. A total of 38 out of 59 food items (64.4%) recorded low MOE values (<10,000). Notably, all *kuih-muih* samples and 80% of the fried and stewed cooked foods analysed yielded values below 10,000. This pattern was also observed in vegetable-based fats and oils (concentrated creamer, shortening, and margarine) as well as foods containing hydrolysed vegetable protein (HVP), e.g., instant noodles, and chicken *soto*. In the fast-food category, only beef burgers posed a risk, primarily due to the lower consumption pattern associated with the remaining food types. Consumption of milk and dairy products (butter, evaporated milk), and certain deep-fried foods (potato fries, sausages, fried chicken, fish crackers) was found to be at risk only for high consumers.

GE exposure presented a less extensive health concern. Only 23.7% of food samples yielded MOE < 10,000, with values ranging from 1763 to 7,831,250 on average consumption. The food categories posing the highest risk were those with high content of savoury seasonings, including flavoured snacks, instant noodles, and fried dishes (rice and noodles). Elevated risk was restricted primarily to high consumers of biscuits, assorted *kuih-muih*, and vegetable-oil spreads (margarine, shortening, and creamer). Despite preparation methods involving deep-frying, prepared snacks (*keropok lekor*, fried fish crackers), some fried foods (fried chicken, butter prawns), and fast-food samples were classified as low risk even at high consumption levels.

In the sex-stratified analysis, similar patterns were observed across males and females. Both groups faced the highest risk of 3-MCPDE and GE health concerns from the *kuih-muih*, cooked fried food, and snack categories at mean consumption levels. Regarding ethnicity, Malays consumed the highest percentage of food items yielding low MOE values (3-MCPDE: 51.4%, GE: 16.2%), followed by the Chinese (3-MCPDE: 37.8%, GE: 13.5%), Others (*Bumiputera*) (3-MCPDE: 21.6%, GE: 21.6%), Indians (3-MCPDE: 18.9%, GE: 16.2%), and Others (minorities) (3-MCPDE: 16.2%, GE: 8.1%). The implicated food categories were consistent across all racial groups, with staple foods such as fried rice, fried wheat/rice noodles, and instant noodles persistently yielding low MOE values across all subpopulations. Detailed data are available in Supplementary File S2.

### 3.4. Estimation of the Potential Risk of Cancer and Burden of Disease from Exposure to Glycidol

Based on the total consumption of foods in our study, the estimated risk for the average Malaysian consumer of developing cancer on lifetime exposure to glycidol was 515.2 cases per 100,000 of the population. The LCR revealed notable variability across the different food groups, primarily due to heterogeneous levels of glycidol contamination. The highest LCR was attributed to the cooked fried food category (301.8 cases/100,000), with a significant contribution from fried rice (164.7 cases/100,000). This was followed by snacks (61.9 cases/100,000) and cooked stewed or boiled foods (56.9 cases/100,000), all of which far exceeded the NSRL threshold value. Only the milk and dairy (1.6 cases/100,000), confectionery (4.1 cases/100,000) and fast food (7.8 cases/100,000) categories presented a comparatively lower risk, with fewer than 10 cases per 100,000 (Table 3A). The mean ACR values mirrored this trend, with higher risks attributed to constant exposure to specific items such as fried rice (2.2 cases/year/100,000), seasoned snacks (0.8 cases/year/100,000), and instant noodles (0.7 cases/year/100,000). Overall, considering all food categories, chronic exposure to glycidol could cause from 7 (mean consumers) to 25 (high consumers) cancer cases/year/100,000.

To estimate the overall health burden, the DALYs were calculated for each food group. The results confirmed that food categories with high LCRs also imposed the greatest burden in terms of DALYs. An evaluation for both mean and high consumers indicated that cooked fried foods were associated with 72.8 DALYs (95% UI: 60.2–87.2) per 100,000 people, followed by snacks with 14.9 DALYs [95% UI: 12.3–17.9] per 100,000, and cooked stewed/boiled foods with 13.7 DALYs [95% UI: 11.4–16.4] per 100,000. The total DALYs for all food categories amounted to 124.2 DALYs (95% UI: 102.7–148.8) per 100,000 for mean consumers, and 450.4 DALYs (95% UI: 372.6–539.6) per 100,000 for high consumers. Individual food items with MOE < 10,000 were identified and tabulated for LCR and DALY in Table 3B. Detailed values are available in the Supplementary File S3.

**Figure 5:**
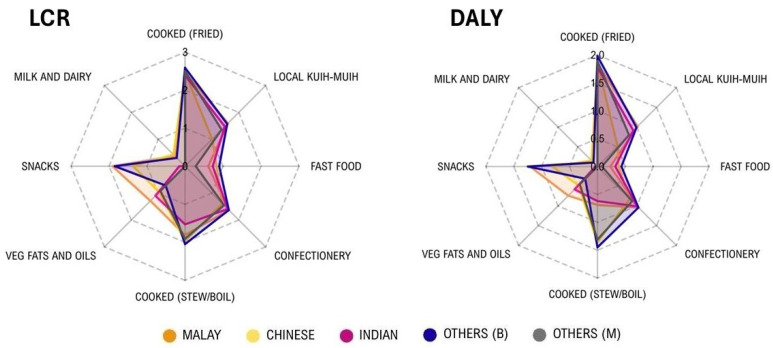
Comparison of risk profiles for GE exposure among race groups according to food categories. The radar chart illustrates the point estimates for LCR and DALY, normalised using a log_10_ (ꭓ + 1) transformation to visualise the distribution across food categories with varying magnitudes of risk. The central axis represents the log-transformed risk value, with the outermost vertices indicating the primary dietary drivers. Larger polygon areas indicate a higher cumulative health burden driven by specific dietary patterns within each subpopulation. Others (B): Others (*Bumiputera*), Others (M): Others (minorities).

The distribution of LCR and DALYs attributable to GE exposure exhibited distinct, culturally mediated profiles across the five ethnic groups, as illustrated in Figure 5. The Others (*Bumiputera*) group displayed the highest cumulative risk burden (LCR: 663 cases/100,000, DALYs: 159.8/100,000), characterised by a broad contribution from multiple categories, particularly fried cooked and cooked stewed foods. The Malay population exhibited a risk profile dominated by the consumption of vegetable fats and oils, snacks, and local *kuih-muih*. While the Chinese and the Other (minorities) cohorts demonstrated relatively lower risk estimates across most food groups, cooked stewed or boiled foods remained a consistent contributor for these ethnicities. For the Indian population, confectionery and local *kuih-muih* emerged as relevant secondary drivers. Notably, the symmetry between the LCR and DALY radar plots suggests that the food categories driving carcinogenic risk are identical to those contributing to the overall chronic disease burden within these populations, thereby highlighting specific dietary targets for risk mitigation.

## 4. Discussion

### 4.1. Dietary Exposure and Risk Assessment of 3-MCPDE and GE Among Malaysians

This study characterises the occurrence of 3-MCPDE and GE contamination in common Malaysian foods and their associated human health risk. We identified the primary sources of contamination for both 3-MCPDE and GE in retail commercial foods to be precursor vegetable oil fats (i.e., margarine, shortening) and snacks (i.e., *murukku*, flavoured snacks). Among cooked foods, the most contaminated categories (*kuih-muih,* fried cooked dishes) involved deep-frying. This aligns with similar studies investigating prepared meals, which indicate that contamination levels are influenced by the mode of preparation and choice of base oil [25]. The practice of using the same oil for multiple cycles of cooking may also play a role, as the conversion of 3-MCPDE to GE has been found to increase significantly with prolonged heating time, emphasising the need to reduce heating cycles during the cooking process [26].

The aggregate dietary exposure to 3-MCPDE reached 5.58 µg/kg BW/day, representing 39.5% above the PMTDI, indicating a potential public health concern. This level significantly exceeds the 0.982 µg/kg BW/day reported in neighbouring Singapore [27]. This divergence is likely attributable to our targeted sampling strategy, which prioritised food matrices with high contamination potential and a high prevalence of palm-based ingredients, such as palm olein, which are known precursors to 3-MCPDE. The difference in local consumption patterns, especially within the Malay-majority and *Bumiputera* demography in Malaysia, may heavily influence the overall exposure risk.

Regarding GE, since there is no established safety threshold, the contamination observed in our samples warrants deep concern. Our shortening and margarine samples contained the highest levels of GE, ranging from 3.35 to 6.34 mg/kg and 0.27 to 9.41 mg/kg, respectively. These findings are consistent with a study from Turkey on precursor fats, which reported values of 1.98–6.46 mg/kg for shortening and 0.19–3.53 mg/kg for margarines [28]. The presence of these contaminants in these fat-rich intermediate products, coupled with high-heat processing (e.g., baking, frying), serves as a direct and prominent pathway for their transfer into baked end-products, including biscuits and confectionery [18,29]. Low MOE values indicate potential health concerns regarding GE, particularly for high consumers of savoury snacks (e.g., potato chips, *murukku*). Vulnerable populations, such as children, frequently consume these types of processed foods, thereby increasing their susceptibility to the associated risks [18].

Combining the exposure from all food categories resulted in an estimated ACR of 6.9 cases per year per 100,000 population. Direct comparison of this value is challenging due to the scarcity of recent studies using the same metric to evaluate risks for the local adult population. For context, an Italian study reported ACR values from glycidol exposure ranging between 0.08 and 0.52 cancer cases per year per 100,000, although this study primarily evaluated toddlers and children [30]. Findings from a recent review study on cancer risk concluded that GE exposure among adults and the elderly has led to a considerable number of cancer cases, with emphasis on breast, lung, and colorectal cancers in certain Asian and European countries [31].

In our study, ethnic-specific risks were highest among the Others (*Bumiputera*) and Malay groups, with 8.8 and 7.08 excess cases per year per 100,000, respectively. The primary factors driving the risks are distinctly different between ethnicities, reflecting unique consumption patterns. Risks for the Others (*Bumiputera*) and Chinese groups were volume-driven, stemming from the high-frequency consumption of cooked staples, such as fried dishes (rice, noodles) and stewed dishes (*soto*, instant noodles). Conversely, exposure in Malay and other minority populations was concentration-driven, primarily through a high intake of contaminated lipid-based products, specifically vegetable fats and oils. The Indian population showed the lowest risk, attributed to a diversified diet that avoids a high intake of refined oils. Despite having lower risk projections, our findings from a previous case–control study on renal cancer prevalence among Malaysians showed a significant presence of the disease among the Chinese. In that study, we concluded there was insufficient clinical evidence to suggest that the ingestion of these contaminants directly contributes to the development of renal malignancies [9].

In terms of DALYs, chronic exposure to GE accounted for 124.2 DALYs per 100,000 for the average Malaysian adult. This translates to a total burden of 42,352 years lost to disability for the total Malaysian population. These results suggest that the cumulative exposure from multiple sources may pose a substantial risk of disease burden, based on the dietary choices of consumers. It should be emphasised that this study focuses on the dietary esterified form of glycidol, rather than free glycidol. A direct comparison of total DALYs for GE across different countries is challenging due to the absence of a standardised global database for this specific food contaminant. However, a preliminary comparison with data reported by Yabani et al. indicates that the estimated GE-related DALYs per capita for the Malaysian population are higher than those for Taiwan (6.69), Spain (30.2), and Poland (19.7), yet considerably lower than the rates reported for China (1020) [31].

### 4.2. Strengths and Limitations of the Study

This investigation provides the first surveillance study regarding 3-MCPDE and GE contamination in prepared and cooked foods within Malaysia. A key strength of this work lies in providing initial insights into contaminant occurrence and the associated risk assessment for the local population. The study utilised a cross-sectional sampling design across six states over two months, enabling a clear estimation of contamination levels within the supply chain during the specified timeframe. Furthermore, the selection of food samples was purposively aligned with typical adult dietary habits. The robustness of the analytical method was enhanced by duplicate analysis for cooked samples, ensuring the accuracy of chloropropanol concentration data. Another strength of our study is the utilisation of nationally published food consumption data, evaluated nationwide through a multi-stage stratified cluster-sampling design, which is representative of the whole adult population. Although the latest consumption data dated back to 2014, it was assumed that cultural influences kept dietary habits consistent, rendering food consumption patterns stable throughout the study period [32].

Despite these strengths, we acknowledge several limitations that may introduce uncertainty into the exposure and risk characterisation. A primary constraint is sample variability, as cooked samples were collected across diverse localities where ingredient composition and recipes were operator-dependent, leading to significant outliers in GE levels among cooked categories. This necessitated the assumption that all similarly named foods are representative of the national average. Additionally, the specific selection of cooked foods known to have high vegetable oil content may result in an overestimation of the true population’s dietary exposure. This uncertainty is compounded by extrapolation factors, such as grouping the consumption of diverse local cakes and desserts under a single *kuih-muih* estimate. The small and uneven sample sizes across food categories may have reduced the statistical power to detect significant differences in exposures between food groups.

Finally, we adopted the deterministic risk assessment approach for average and high consumers. This relies on simplifying assumptions (e.g., all individuals have the same body weight and consume the same contaminant concentration for a given food item) [33], and does not utilise the use of statistical distributions to model the full range of possible exposures and their probabilities. The exclusion of other potential carcinogenic factors and the necessary use of an indirect method for calculating the burden of disease also contribute to the uncertainty and potential overestimation of actual risks and health burden [34]. However, as the primary aim of this study was to provide surveillance data for risk management, the deterministic assessment was deemed sufficient for screening and guiding policy decision-making [35].

### 4.3. Future Research Directions and Recommendations

The effective mitigation of 3-MCPDE and GE contamination in vegetable oil remains a critical, ongoing challenge that requires both technical and policy-driven interventions. Contaminant formation occurs primarily during deodorisation, highlighting the urgent need for processors to adopt optimised refining techniques [36]. Continued research is vital, focusing on new mitigation technologies like pre-deacidification, adsorbent treatments, and enzymatic processing, which show varying efficacy based on the oil type [37,38]. Other proven strategies, like crude palm oil washing and vacuum stripping, should see broader adoption in initial processing [39]. Upstream interventions must prioritise mitigating agricultural practices, specifically aiming to reduce chloride-based fertilisers and minimise post-harvest delays to limit precursor formation [8]. Furthermore, emerging research on natural inhibitors, such as nettles, offers a promising avenue for reducing GE formation during frying and thermal processing [40].

Safeguarding public health and maintaining global confidence in Malaysian palm oil necessitates the rigorous implementation and monitoring of national standards aligned with international benchmarks. In adherence to the 2019 Codex Alimentarius Commission Code of Practice (CXC 79-2019) regarding the reduction of 3-MCPDE and GE in refined oils and related foods [41], integration between the Malaysian Palm Oil Board (MPOB) and key stakeholders, including local planters, millers, and refiners, must be strengthened. Given that this study reports on contamination data from samples collected in 2017, it is recommended that a follow-up surveillance study be conducted subsequent to the full implementation of these regulatory standards. Such an initiative is essential to comprehensively evaluate the effectiveness of mitigation strategies in controlling these contaminant levels within the food supply.

## 5. Conclusions

This research provides the first comprehensive assessment of the occurrence and human health risks associated with 3-MCPDE and GE contamination in selected Malaysian food products. Our results show that exposure to these contaminants across the adult population is concerning. The estimated aggregate daily intake of 3-MCPDE reached 5.58 µg/kg BW/day, which exceeds the PMTDI set by JECFA. Because this high exposure is largely driven by everyday staples like fried rice, it highlights a pressing and previously unquantified public health issue in the Malaysian diet. Elevated risks are particularly pronounced among high consumers of specific food categories, notably those containing refined vegetable oils and prepared via deep-frying.

Beyond 3-MCPDE, chronic exposure to GE also points to a substantial potential disease burden under chronic exposure scenarios. GE contamination was found to be widespread, primarily in vegetable fats, packaged snacks, and traditional local delicacies (*kuih-muih*). The estimated LCR and DALYs from exposure to GE reveals that these risks are not evenly distributed across subpopulations; rather, they are strongly tied to cultural dietary habits. These demographic differences are crucial for public health authorities to understand, as they suggest that targeted, culturally specific dietary interventions will likely be much more effective than generic, population-wide advisories.

Tackling 3-MCPDE and GE contamination will ultimately require a coordinated effort across the entire supply chain. While raising consumer awareness and encouraging better cooking practices, such as avoiding repeated oil heating can help reduce exposure at home, the core issue stems from industrial processing. These findings necessitate the immediate implementation of a multi-faceted national strategy, integrating effective regulation, collaborative industry efforts, and technological innovation. Such measures are essential to ensure the sustainable reduction of 3-MCPDE and GE levels, protecting consumer health as well as maintaining global confidence in Malaysia’s agricultural exports.

## Figures and Tables

**Table 1:** 3-MCPDE and GE occurrence levels (mg/kg) in Malaysian food items (by category).

FOOD CATEGORY	No. of Samples	Total 3-MCPDE (mg/kg Wet Weight)					Total GE (mg/kg Wet Weight)				
		LOD	LOQ	UB Mean	UB Median	Range	LOD	LOQ	UB Mean	UB Median	Range
**Vegetable Fats and Oils**	**42**			**1.60**	**0.71**				**2.55**	**1.47**	
Margarine	23	0.07	0.21	2.50	2.39	0.22–6.79	0.09	0.27	3.70	3.60	<LOQ–9.41
Mayonnaise	3	0.11	0.16	0.14	0.16	<LOQ	0.04	0.31	0.13	0.04	<LOQ
Shortening	4	0.07	0.21	4.38	3.53	2.70–7.78	0.09	0.27	4.81	4.78	3.35–6.34
Evaporated creamer	7	0.07	0.21	0.19	0.21	<LOQ	0.09	0.27	0.14	0.09	<LOQ
Concentrated creamer	5	0.07	0.21	0.76	0.71	0.41–1.21	0.09	0.27	0.23	0.27	<LOQ
**Milk and Dairy**	**16**			**0.29**	**0.39**				**0.28**	**0.09**	
Evaporated milk	3	0.07	0.21	0.21	0.21	<LOQ	0.09	0.27	0.15	0.09	<LOQ
Butter	13	0.07	0.21	0.38	0.39	<LOQ–0.91	0.09	0.27	0.42	0.09	<LOQ–2.10
**Confectionery**	**53**			**0.35**	**0.29**				**0.55**	**0.35**	
Flavoured biscuit	8	0.07	0.21	0.53	0.40	<LOQ–1.19	0.09	0.27	1.05	1.07	<LOQ–2.22
Plain biscuit	6	0.07	0.21	0.45	0.43	0.26–0.70	0.09	0.27	1.31	1.25	0.34–2.38
Bun	6	0.07	0.21	0.11	0.07	<LOQ	0.09	0.27	0.14	0.04	<LOQ–0.35
Cake	13	0.07	0.21	0.40	0.21	<LOQ–1.45	0.09	0.27	0.39	0.35	<LOQ–1.08
Doughnut	3	0.11	0.16	0.56	0.41	0.26–1.01	0.04	0.31	0.56	0.35	<LOQ–1.03
Cheese tart	2	0.07	0.21	0.15	0.15	<LOQ–0.22	0.09	0.27	0.36	0.36	0.31–0.42
Chocolate bar	7	0.07	0.21	0.09	0.07	<LOQ	0.09	0.27	0.14	0.09	<LOQ
Chocolate spread	8	0.07	0.21	0.52	0.38	<LOQ–1.07	0.09	0.27	0.42	0.27	<LOQ–0.81
**Snacks**	**29**			**0.54**	**0.45**				**0.96**	**0.86**	
*Keropok lekor*	3	0.11	0.16	0.13	0.11	<LOQ	0.04	0.31	0.04	0.04	<LOQ
Fried fish crackers	3	0.11	0.16	0.74	0.59	0.30–1.34	0.04	0.31	0.78	0.61	0.53–1.20
*Murukku*	5	0.11	0.16	1.37	0.94	0.58–3.20	0.04	0.31	1.88	0.92	0.47–4.89
Potato chips	5	0.07	0.21	0.42	0.45	<LOQ–0.70	0.09	0.27	0.90	0.96	0.30–1.41
Chicken-flavoured snack	4	0.07	0.21	0.29	0.21	<LOQ–0.52	0.09	0.27	0.92	0.76	0.30–1.85
Seafood-flavoured snack	4	0.07	0.21	0.47	0.46	0.26–0.70	0.09	0.27	1.31	1.55	1.53–1.82
Fruit/vegetable-flavoured snack	5	0.07	0.21	0.39	0.41	<LOQ–0.76	0.09	0.27	0.88	0.86	<LOQ–1.65
**Local *Kuih-Muih***	**30**			**0.65**	**0.68**				**0.77**	**0.62**	
*Kuih denderam*	3	0.11	0.16	1.08	1.27	0.49–1.49	0.04	0.31	0.46	0.4	<LOQ–0.67
Prawn fritter	3	0.11	0.16	1.02	0.68	0.43–1.94	0.04	0.31	0.76	0.64	0.34–1.31
Curry puff	3	0.11	0.16	0.73	0.73	0.43–1.04	0.04	0.31	1.08	1.17	0.82–1.24
*Cakoi*	3	0.11	0.16	0.71	0.76	0.42–0.94	0.04	0.31	0.46	0.36	0.36–0.72
*Vadai*	3	0.11	0.16	0.69	0.67	0.58–0.82	0.04	0.31	0.57	0.6	0.43–0.68
Banana fritters	3	0.11	0.16	0.67	0.66	0.34–1.01	0.04	0.31	0.98	0.95	0.33–1.65
Fried spring rolls	3	0.11	0.16	0.67	0.73	0.51–0.76	0.04	0.31	0.95	0.93	0.40–1.53
Fried *cempedak*	3	0.11	0.16	0.44	0.37	0.35–0.60	0.04	0.31	1.37	1.17	1.14–1.81
Fried sweet potato	3	0.11	0.16	0.36	0.35	0.25–0.48	0.04	0.31	0.66	0.53	0.50–0.94
Fried banana balls	3	0.11	0.16	0.17	0.16	<LOQ–0.24	0.04	0.31	0.41	0.42	<LOQ–0.51
**Cooked (Fried)**	**48**			**0.57**	**0.41**				**0.45**	**0.31**	
Fried rice	3	0.11	0.16	0.66	0.72	0.39–0.87	0.04	0.31	0.35	0.31	<LOQ–0.42
*Char-kuey-teow*	3	0.11	0.16	0.50	0.59	0.30–0.62	0.04	0.31	0.22	0.31	<LOQ
Fried rice noodles	3	0.11	0.16	0.33	0.41	<LOQ–0.47	0.04	0.31	0.22	0.31	<LOQ
Fried wheat noodle	3	0.11	0.16	0.24	0.23	<LOQ–0.33	0.04	0.31	0.22	0.31	<LOQ
Fried Indian mackerel	3	0.11	0.16	0.23	0.24	<LOQ–0.33	0.04	0.31	0.22	0.31	<LOQ
*Roti canai*	3	0.11	0.16	0.50	0.45	0.43–0.61	0.04	0.31	0.42	0.36	<LOQ–0.58
Fried anchovies	3	0.11	0.16	1.60	1.67	1.43–1.71	0.04	0.31	1.41	1.22	0.98–2.03
Butter prawn	3	0.11	0.16	2.38	1.05	1.04–5.04	0.04	0.31	1.29	1.3	1.24–1.33
Fried beef	3	0.11	0.16	0.73	0.84	0.35–0.99	0.04	0.31	0.47	0.48	<LOQ–0.63
*Bergedil daging*	3	0.11	0.16	0.45	0.46	0.43–0.47	0.04	0.31	0.64	0.62	0.60–0.70
Beef *biryani*	3	0.11	0.16	0.42	0.41	0.37–0.47	0.04	0.31	0.42	0.39	0.39–0.48
Beef *martabak*	3	0.11	0.16	0.15	0.16	<LOQ–0.17	0.04	0.31	0.13	0.04	<LOQ
Beef *satay*	3	0.11	0.16	0.27	0.24	0.21–0.35	0.04	0.31	0.50	0.42	<LOQ–0.76
Fried chicken	3	0.11	0.16	0.24	0.11	<LOQ–0.51	0.04	0.31	0.31	0.31	<LOQ
Fried macaroni	3	0.11	0.16	0.23	0.11	<LOQ–0.46	0.04	0.31	0.22	0.31	<LOQ
Fish ball	3	0.11	0.16	0.25	0.24	0.23–0.28	0.04	0.31	0.22	0.31	<LOQ
**Fast Food**	**18**			**0.20**	**0.17**				**0.22**	**0.31**	
Beef burger patty	3	0.11	0.16	0.33	0.30	0.26–0.42	0.04	0.31	0.41	0.34	<LOQ–0.58
Potato fries	3	0.11	0.16	0.24	0.16	<LOQ–0.46	0.04	0.31	0.31	0.31	<LOQ
Frankfurter/sausage	3	0.11	0.16	0.18	0.17	<LOQ–0.21	0.04	0.31	0.04	0.04	<LOQ
Nuggets	3	0.11	0.16	0.20	0.20	<LOQ–0.23	0.04	0.31	0.22	0.31	<LOQ
Hot dog	3	0.11	0.16	0.14	0.16	<LOQ	0.04	0.31	0.33	0.31	<LOQ–0.36
Pizza with beef	3	0.11	0.16	0.11	0.11	<LOQ	0.04	0.31	0.04	0.04	<LOQ
**Cooked (Stew/Boil)**	**15**			**0.29**	**0.18**				**0.38**	**0.31**	
Instant noodles	3	0.11	0.16	0.28	0.18	<LOQ–0.49	0.04	0.31	0.66	0.39	<LOQ–1.27
Beef in soy sauce	3	0.11	0.16	0.28	0.26	<LOQ–0.46	0.04	0.31	0.31	0.31	<LOQ
Chicken *soto*	3	0.11	0.16	0.11	0.11	<LOQ	0.04	0.31	0.13	0.04	<LOQ
Beef curry	3	0.11	0.16	0.70	0.81	0.24–1.05	0.04	0.31	0.48	0.49	<LOQ–0.65
*Satay* sauce	3	0.11	0.16	0.11	0.11	<LOQ	0.04	0.31	0.31	0.31	<LOQ

LOQ: Level of quantification (following values reported by respective laboratories), UB: upper border estimate, which assumes all non-quantified values are present at the maximum possible concentration and substituted with the LOQ level.

**Table 2:** Dietary exposure (µg/kg BW/day) to (**A**) 3-MCPDE and (**B**) GE per food category for the total adult population.

(**A**)						
**FOOD CATEGORY**	**3-MCPDE**					
	**MEAN CONSUMER**			**HIGH CONSUMER (p95)**		
	**IR**	**DE**	**% of** **total PMTDI**	**IR**	**DE**	**% of** **total PMTDI**
Vegetable Fats and Oils	0.02	0.23	5.76	0.09	1.22	30.50
Milk And Dairy	0.00	0.01	0.33	0.02	0.08	2.00
Confectionery	0.03	0.14	3.41	0.13	0.58	14.50
Snacks	0.04	0.27	6.78	0.17	1.26	31.50
Local *Kuih-Muih*	0.02	0.23	5.75	0.09	0.92	23.00
Cooked (Fried)	0.49	4.37	109.22	1.56	13.53	338.25
Fast Food	0.02	0.05	1.35	0.06	0.22	5.50
Cooked (Stewed/Boiled)	0.10	0.28	6.90	1.00	1.17	29.25
**TOTAL**	**0.71**	**5.58**	**139.50**	**3.12**	**18.98**	**474.50**
(**B**)						
**FOOD CATEGORY**	**GE**					
	**MEAN CONSUMER**			**HIGH CONSUMER (p95)**		
	**IR**	**DE**	**% of** **total DE**	**IR**	**DE**	**% of** **total DE**
Vegetable Fats and Oils	0.02	0.13	2.31	0.09	0.68	3.60
Milk and Dairy	0.00	0.01	0.24	0.02	0.08	0.42
Confectionery	0.03	0.32	5.82	0.13	1.37	7.34
Snacks	0.04	0.51	9.28	0.17	2.37	12.64
Local *Kuih-Muih*	0.02	0.26	4.69	0.09	1.03	5.52
Cooked (Fried)	0.49	2.50	45.20	1.56	7.99	42.62
Fast Food	0.01	0.07	1.19	0.06	0.27	1.46
Cooked (Stewed/Boiled)	0.35	1.73	31.27	1.00	4.94	26.39
**TOTAL**	**0.96**	**5.52**	**100**	**0.09**	**18.73**	**100**

IR: Intake rate of food category expressed in kg/day. % of total PMTDI: The percentage contribution of each food category to the Provisional maximum tolerable daily intake (PMTDI) for 3-MCPDE, established by JECFA at 4 µg/kg BW/day. DE: Representative dietary exposure value of food category using the consumption-weighted average concentration of constituent food items (µg/kg BW/day). % of total DE: The percentage contribution of each food category to the total DE.

**Table 3:** Estimation of cancer risk potential and burden of disease (DALY) from exposure to GE following (**A**) consumption pattern (mean and high consumer) according to food categories, and (**B**) mean consumption of food items with a low MOE (<10,000), stratified by sex.

(**A**)												
**FOOD CATEGORY**	**MEAN CONSUMER**						**HIGH CONSUMER (p95)**					
	**LCR**		**ACR**		**DALY**		**LCR**		**ACR**		**DALY**	
Vegetable Fats and Oils	15.4		0.2		3.7		81.6		1.1		19.7	
Milk And Dairy	1.6		0.0		0.4		9.4		0.1		2.3	
Confectionery	4.1		0.1		0.4		17.5		0.2		1.7	
Snacks	61.9		0.8		14.9		285.8		3.8		68.9	
Local *Kuih-Muih*	31.2		0.4		7.5		124.6		1.7		30.0	
Cooked (Fried)	301.8		4.0		72.8		928.5		12.3		223.8	
Fast Food	7.8		0.1		1.9		32.6		0.4		7.9	
Cooked (Stewed/Boiled)	56.9		0.8		13.7		241.2		3.2		58.1	
**Total**	**515.2**		**6.9**		**124.2**		**1868.8**		**24.9**		**450.4**	
(**B**)												
**FOOD ITEMS**	**TOTAL POPULATION**				**ADULT MALES**				**ADULT FEMALES**			
	**LCR**	**ACR**		**DALY**	**LCR**	**ACR**		**DALY**	**LCR**	**ACR**		**DALY**
Fried rice	164.7	2.2		16.1	219.9	2.7		41.1	152.1	2.0		27.7
Seasoned snacks (*murukku*, potato chips, flavoured snacks)	59.4	0.8		14.3	54.5	0.8		27.9	75.5	1.0		34.5
Instant noodles	48.6	0.7		4.8	59.9	0.8		12.5	42.7	0.6		7.8
*Char kuey teow*, fried rice noodles	39.1	0.5		9.4	41.7	0.6		21.4	42.4	0.5		19.3
Fried wheat noodle	38.6	0.5		3.8	33.8	0.7		9.9	24.1	0.4		6.2
Fried Indian mackarel	28.0	0.4		2.7	28.8	0.4		6.0	31.7	0.4		5.8
*Kuih-muih*	31.2	0.4		7.5	54.5	0.8		27.9	33.0	0.4		15.1
Plain biscuit	26.2	0.4		2.6	23.4	0.3		4.9	33.9	0.4		6.2
Chicken *soto*	21.3	0.3		2.1	25.4	0.4		5.3	19.6	0.3		3.6
Concentrated creamer	6.59	0.09		0.64	8.1	0.1		1.7	5.8	0.1		1.1
Fried beef, *biryani*, *bergedil*, *satay*	4.93	0.1		1.19	6.59	0.1		3.38	3.7	0.1		1.7

LCR: Lifetime cancer risk (per 100,000 population); ACR: annual carcinogenic risk (cases/year/100,000 population); DALY: Disability-Adjusted Life Years (per 100,000 cases).

## Data Availability

The original contributions presented in this study are included in the article/Supplementary Material. Further inquiries can be directed to the corresponding author.

## Supplementary Materials

The following supporting information can be downloaded at https://www.mdpi.com/article/10.3390/toxics14040331/s1, Supplementary File S1.1: Analytical Methodology for the Determination of 3-MCPD and Glycidyl Esters, Supplementary File S1: Food mapping and consumption data of the total adult population obtained from the Malaysian Adult Nutrition Survey (MANS) 2014; Supplementary File S2: Dietary exposure and MOE for subpopulations; Supplementary File S3: LCR, ACR, and DALYs for subpopulations.

## Abbreviations

The following abbreviations are used in this manuscript:

3-MCPDEs	3-Monochloropropane-1,2-diol Esters
AOCS	American Oil Chemists’ Society
BW	Body Weight
CSF	Cancer Slope Factor
DALYs	Disability-Adjusted Life Years
EC	European Commission
EDI	Estimated Daily Intake
EFSA	The European Food Safety Authority
GC-MS	Gas Chromatography–Mass Spectrometry
GEs	Glycidyl Esters
HBGV	Health-Based Guidance Value
HVP	Hydrolysed Vegetable Protein
JECFA	The Joint FAO/WHO Expert Committee on Food Additives
LADD	Lifetime Average Daily Dose
LCR	Lifetime Cancer Risk
LOD	Level of Detection
LOQ	Level of Quantification
MANS	Malaysian Adult Nutrition Survey
MOE	Margin of Exposure
NSRL	No Significant Risk Level
PMTDI	Provisional Maximum Tolerable Daily Intake
UB	Upper Bound
UI	Uncertainty Interval

## References

[1] Singh S.K., Awasthi N.P., Yadav P. (2025). Challenges and Mitigation Strategies for Control of 3-MCPDEs and GEs in Edible Oil Processing Industries—A comprehensive review. Food Chem. Adv..

[2] Yung Y.L., Lakshmanan S., Kumaresan S., Chu C.M., Tham H.J. (2023). Mitigation of 3-monochloropropane 1,2 diol ester and glycidyl ester in refined oil—A review. Food Chem..

[3] International Agency for Research on Cancer (IARC) (2013). Some Chemicals Present in Industrial and Consumer Products, Food and Drinking-Water; IARC Monographs on the Evaluation of Carcinogenic Risks to Humans.

[4] Office of Environmental Health Hazard Assessment (OEHHA) (2010). No Significant Risk Level (NSRL) for the Proposition 65 Carcinogen Glycidol.

[5] National Toxicology Program (NTP) (2021). RoC Profile: Glycidol CAS No. 556-52-5. Report on Carcinogens.

[6] European Commission (2023). Commission Regulation (EU) 2023/915 of 25 April 2023 on maximum levels for certain contaminants in food and repealing Regulation (EC) No 1881/2006. Off. J. Eur. Union.

[7] Joint FAO/WHO Expert Committee on Food Additives (JECFA) (2018). 3-Monochloro-1,2-Propanediol Esters and 3-Monochloro-1,2-Propanediol. Safety Evaluation of Certain Contaminants in Food: Prepared by the Eighty-Third Meeting of the Joint FAO/WHO Expert Committee on Food Additives (JECFA).

[8] Ong Y.H., Song C.P., Choo W.S., Lee Y.Y., Qua K.S., Quek W.P., Chan E.S. (2025). Fatty acid esters of 3-monochloropropane-1,2-diol and glycidol in palm oil: A review on current industrial-scale mitigation strategies, challenges and perspectives. Food Res. Int..

[9] Muhamad Rosli S.H., Lau M.S., Khalid T., Maarof S.K., Jeyabalan S., Sirdar Ali S., Mustafa Khalid N., Md Noh M.F., Salleh R., Palaniveloo L. (2023). Association between dietary 3-monochloropropane-1, 2-diol esters (3-MCPDE) and renal cancer in Peninsular Malaysia: Exposure assessment and matched case-control study. Food Addit. Contam. Part A.

[10] European Commission (EC) (2014). Commission Recommendation of 10 September 2014 on the monitoring of the presence of 2 and 3-monochloropropane-1,2-diol (2 and 3-MCPD), 2- and 3-MCPD fatty acid esters and glycidyl fatty acid esters in food Text with EEA relevance (2014/661/EU). Off. J. Eur. Union.

[11] Institute for Public Health (IPH) (2014). National Health and Morbidity Survey 2014: Malaysian Adult Nutrition Survey (MANS).

[12] American Oil Chemists’ Society (AOCS) (2017). Official Method Cd 29c-13. 2- and 3-MCPD Fatty Acid Esters and Glycidol Fatty Acid Esters in Edible Oils and Fats by GC/MS (Difference Method), Revised 2017. Official Methods and Recommended Practises of the AOCS.

[13] European Food Safety Authority (EFSA) (2010). Management of left—Censored data in dietary exposure assessment of chemical substances. EFSA J..

[14] Nutrition Section (Family Health Development Division) (2008). Nutritional Status of Adults Aged 18–59 Years.

[15] The European Food Safety Authority (EFSA) (2012). Statement on the applicability of the Margin of Exposure approach for the safety assessment of impurities which are both genotoxic and carcinogenic in substances added to food/feed. EFSA J..

[16] Edler L., Hart A., Greaves P., Carthew P., Coulet M., Boobis A., Williams G.M., Smith B. (2014). Selection of appropriate tumour data sets for Benchmark Dose Modelling (BMD) and derivation of a Margin of Exposure (MoE) for substances that are genotoxic and carcinogenic: Considerations of biological relevance of tumour type, data quality and uncertainty assessment. Food Chem. Toxicol..

[17] EFSA Scientific Committee (2017). Update: Use of the benchmark dose approach in risk assessment. EFSA J..

[18] EFSA Panel on Contaminants in the Food Chain (CONTAM) (2016). Risks for human health related to the presence of 3- and 2-monochloropropanediol (MCPD), and their fatty acid esters, and glycidyl fatty acid esters in food. EFSA J..

[19] World Health Organisation (WHO) (2017). Evaluation of Certain Contaminants in Food: Eighty-Third Report of the Joint FAO/WHO Expert Committee on Food Additives.

[20] Department of Statistics Malaysia (DOSM) Demography: Life Expectancy. https://open.dosm.gov.my/dashboard/life-expectancy.

[21] Office of Environmental Health Hazard Assessment (OEHHA) (2020). Proposition 65 Maximum Allowable Dose Level (MADL) for Developmental and Reproductive Toxicants and No Significant Risk Level (NSRL) for Carcinogens (Glycidol).

[22] World Health Organisation (WHO) The Global Health Observatory. Global Health Estimates: Deaths by Cause, Age, Sex, by Country and by Region 2000–2021. https://www.who.int/data/gho/data/themes/mortality-and-global-health-estimates/global-health-estimates-leading-causes-of-dalys.

[23] Bray F., Laversanne M., Sung H., Ferlay J., Siegel R.L., Soerjomataram I., Jemal A. (2024). Global cancer statistics 2022: GLOBOCAN estimates of incidence and mortality worldwide for 36 cancers in 185 countries. CA A Cancer J. Clin..

[24] Global Burden of Disease Collaborative Network (GBD). Global Burden of Disease Study 2021. https://vizhub.healthdata.org/gbd-results/.

[25] Goh K.M., Wong Y.H., Tan C.P., Nyam K.L. (2021). A summary of 2-, 3-MCPD esters and glycidyl ester occurrence during frying and baking processes. Curr. Res. Food Sci..

[26] Mahiran S.N.S.N., Abd Kadir N.H., Maulidiani M., Mohamad T.R.T., Gooderham N.J., Alam M. (2023). Multivariate modelling analysis for prediction of glycidyl esters and 3-monochloropropane-1, 2-diol (3-MCPD) formation in periodically heated palm oil. Heliyon.

[27] Shi R.R.S., Shen P., Yu W.Z., Cai M., Tay A.J., Lim I., Chin Y.S., Ang W.M., Er J.C., Lim G.S. (2023). Occurrence and dietary exposure of 3-MCPD esters and glycidyl esters in domestically and commercially prepared food in Singapore. Foods.

[28] Gündüz A., Ceylan M., Baştürk A. (2023). 3-MCPD and glycidol levels in edible oils and fats obtained from local markets in Türkiye. Grasas Y Aceites.

[29] Goh K.M., Wong Y.H., Abas F., Lai O.M., Cheong L.Z., Wang Y., Wang Y., Tan C.P. (2019). Effects of shortening and baking temperature on quality, MCPD ester and glycidyl ester content of conventional baked cake. LWT.

[30] Mihalache O.A., Dall’Asta C. (2023). Food processing contaminants: Dietary exposure to 3-MCPD and glycidol and associated burden of disease for Italian consumers. Environ. Res..

[31] Yabani D.S., Ofosu I.W., Ankar-Brewoo G.M., Lutterodt H.E. (2024). Exposure to Dietary Glycidyl and 3-MCPD Fatty Acid Esters and Associated Burden of Cancer in Selected Asian and European Countries: A Review and Data Synthesis. Environ. Health Insights.

[32] Nemeth N., Rudnak I., Ymeri P., Fogarassy C. (2019). The role of cultural factors in sustainable food consumption—An investigation of the consumption habits among international students in Hungary. Sustainability.

[33] World Health Organization (WHO) (2020). Principles and Methods for the Risk Assessment of Chemicals in Food; Environmental Health Criteria 240. (Revised Edition).

[34] Jang S., Shao K., Chiu W.A. (2023). Beyond the cancer slope factor: Broad application of Bayesian and probabilistic approaches for cancer dose-response assessment. Environ. Int..

[35] The European Food Safety Authority (EFSA) (2007). Opinion of the Scientific Committee related to Uncertainties in Dietary Exposure Assessment. EFSA J..

[36] Kantekin-Erdogan M.N., Emektar K., Yorulmaz A., Tekin A. (2024). Effects of pre-deacidification on 3-MCPDE and GE contents of high-acid oils during refining. Int. J. Food Sci. Technol..

[37] Yıldız K., Özdikicierler O., Ergönül P.G. (2025). Investigating the role of adsorbent type and ratio in mitigating 3-MCPD and GE formation during the inhibition of palm oil chemical interesterification via earth treatment. Food Chem..

[38] Zhang Y., Feng Z., Sun S. (2025). Investigating the synergistic effects of acylglycerols on the selective formation of 3-MCPD and glycidyl esters in model soybean oil systems. Food Control.

[39] Ozluk G., González-Curbelo M.Á., Kabak B. (2024). Chloropropanols and Their Esters in Food: An Updated Review. Foods.

[40] Yıldız K., İyilikeden E., Yıldız O., Günç Ergönül P. (2025). Investigation of 3-monochloropropane-1, 2-diol and glycidyl ester levels in French fries obtained after frying process using palm olein oil with artificial and natural antioxidants. Nutr. Food Sci..

[41] Codex Alimentarius (2019). Code of Practice for the Reduction of 3-Monochloropropane-1, 2-Diol Esters (3-MCPDEs) and Glycidyl Esters (GEs) in Refined Oils and Food Products Made with Refined Oils (CXC 79-2019).

