# Supplementary material for: Hidden Hazards: Assessment of Exposure Risks from 3-Monochloropropane-1,2-diol Ester (3-MCPDE) and Glycidyl Ester (GE) Consumption Among Malaysian Consumers
Source: Toxics. 2026 Apr 16;14(4):331. doi: 10.3390/toxics14040331 (PMC13119714; doi:10.3390/toxics14040331)
Supplement: Supplementary file 1 [file toxics-14-00331-s001.zip › Supplementary S1 (MANS consumption data).pdf]

Supplementary S1: Food mapping and consumption data of the total adult population obtained from the Malaysian Adult Nutrition Survey (MANS) 2014

Representative food item (*names as listed in MANS 2014)*	Selected items that compose the food	Mean Consumer (g/day/person)	High consumer (p95) (g/day/person**)**
**VEGETABLE FATS AND OILS**			
Salad dressing	Mayonnaise	0.48	1.99
Marjerin	Margarine<br>Shortening	1.03	5.55
Susu pekat manis	Concentrated creamer	14.59	76.00
**MILK AND DAIRY**			
Mentega	Butter	1.48	7.97
Susu sejat/cair	Evaporated milk<br>Evaporated creamer	1.30	8.54
**FAST FOOD**			
Nugget	Nugget	1.36	5.91
Kentang goreng	French fries	2.37	11.10
Frankfurter/sausage/hotdog	Frankfurter/sausage/Hot dog	1.91	14.52
Pizza	Pizza with beef and onion	1.74	7.59
Burger	Beef burger	7.32	22.21
**CONFECTIONERIES**			
Roti ban	Bun	11.69	44.13
Biskut berperisa/berkrim/berinti	Biscuit (cream-filled)	3.43	17.08
Biskut tawar/krim kraker	Plain biscuit	10.38	45.00
Kek	Cake<br>Cheesecake	1.94	10.07
Pastri	Doughnut<br>Cheese tart	0.41	1.64
Coklat bar	Chocolate bar	2.42	11.39
Sapuan coklat	Chocolate spread	0.24	0.00
**LOCAL *KUIH-MUIH***			
Kuih Muih	Deep-fried banana balls<br>Prawn fritter<br>Fried banana fritter<br>*Vadai*<br>Curry puff<br>Fried spring rolls<br>*Cakoi*<br>*Kuih denderam*<br>Fried sweet potato<br>Fried *cempedak*	22.52	90.00
**SNACKS**			
Keropok lekor	Fried fish sausage *(Keropok lekor)*	2.79	11.39
Keropok ikan/udang/ikan/ketam	Fish crackers *(Keropok Ikan)*	1.89	9.11
Snek/kerepek	*Murukku*<br>Potato chips<br>Chicken flavoured snack<br>Seafood flavoured snack<br>Fruit/vegetable flavoured snack	32.24	150.00
**COOKED FOOD (FRIED/STEWED/BOILED**			

Ikan laut	Fried Indian Mackerel	46.82	128.00
Mee kuning/mee siput/mee segera	Fried wheat noodle<br>Instant Noodles	64.51	246.01
Udang basah	Butter Prawn	2.00	8.54
Bebola ikan/udang/sotong/ketam	Fish Ball	2.96	14.24
Nasi putih	Fried rice<br>Chicken *soto*	275.03	720.00
Pasta	Fried macaroni	4.27	17.37
Mihun/kuayteow/laksa	Fried rice noodle<br>*Char kuay teow*	65.34	211.42
Lembu/kerbau (beef)	Fried beef<br>Beef *beriani*<br>Beef curry<br>Beef in soya sauce<br>Beef skewers<br>*Bergedil daging*	5.34	17.08
Ikan bilis	Fried anchovies	3.78	18.00
Ayam goreng	Fried chicken	4.57	16.80
Roti canai	Indian flat bread *(Roti canai)*<br>Beef *murtabak*	14.12	52.96
Sambal	Satay sauce	4.20	16.00
