# Supplementary material for: Hidden Hazards: Assessment of Exposure Risks from 3-Monochloropropane-1,2-diol Ester (3-MCPDE) and Glycidyl Ester (GE) Consumption Among Malaysian Consumers
Source: Toxics. 2026 Apr 16;14(4):331. doi: 10.3390/toxics14040331 (PMC13119714; doi:10.3390/toxics14040331)
Supplement: Supplementary file 1 [file toxics-14-00331-s001.zip › Supplementary S1.1 (GCMS).pdf]

Supplementary S1.1

Analytical Methodology for the Determination of 3-MCPD and Glycidyl Esters

Performing Laboratory: Department of Chemistry Malaysia
(ISO/IEC 17025 & 17043)
Report Date: 26 July 2019.

Reference Methods: The protocol is strictly aligned with **AOCS Official Method Cd 29a-13** for the analysis of 3-MCPD and Glycidyl esters in edible fats and oils.

1. Scope and Principle

This validated indirect method facilitates the simultaneous determination of fatty acid esters of 2-MCPD, 3-MCPD, and glycidol in edible oils and oil-based food products. The procedure relies on acid transesterification to release free diols, followed by derivatisation with phenylboronic acid (PBA) to form volatile derivatives suitable for GC-MS analysis.

- Working Range: 0.3 μg/g to 9.3 μg/g for 2- and 3-MCPD and 0.6 μg/g to 21.3 μg/g for glycidol.
- Quantification: Results are expressed as free-form equivalents using deuterated internal standards for matrix compensation.

2. Standard Preparation and Calibration

2.1. Reference Standards

The following analytical standards were utilized for calibration and internal standardization:

- Native Standards: rac 1,2-bis-palmitoyl-3-chloropropanediol (PP-3-MCPD), 1,3-distearoyl-2-chloropropanediol (PP-2-MCPD), and Glycidyl palmitate (Gly-P).
- Internal Standards (IS): Deuterated analogs PP-3-MCPD-d5, PP-2-MCPD-d5, and Glycidyl oleate-D5 (Gly-O-d5).

2.2. Calibration Matrix

Calibration was performed using a matrix-matched approach with 0.1 g of blank olive oil.

- IS Concentration: Constant addition of 0.39 μg for 3-MCPD-d5 and 2-MCPD-d5, and 0.62 μg for Gly-d5 across all calibration levels (Cal 0–Cal 8).

- Analyte Range: Calibration levels for native 3-MCPD and 2-MCPD ranged from 0.03 µg to 0.93 µg per 0.1 g sample.

3. Detailed Sample Processing

3.1. Extraction from Oil-Based Matrices

1. Initial Extraction: 0.5 g of food sample is treated with 3 mL n-heptane/MTBE (1:2) and 2 mL UHQ water.
2. Phase Separation: Samples are vortexed, incubated at 60°C for 10 min, sonicated, and centrifuged at 4500 rpm (20°C)
3. Purification: The aqueous layer is discarded; the organic layer is washed with UHQ water and evaporated to dryness under nitrogen at 85°C

3.2. Transesterification and Derivatisation

1. Glycidol Conversion: Glycidyl esters are converted to 3-monobromopropanediol (3-MBPD) monoesters using an acidic NaBr solution at 50°C for 15 min.
2. Cleavage: Free forms are released using 1.8% sulphuric acid/methanol solution, with incubation at 60°C for 16–20 hours.
3. PBA Derivatisation: Following a defatting step with n-heptane, 250 µL of saturated PBA solution is added to the residue to form cyclic boronates.
4. Final Recovery: Derivatives are extracted into n-heptane and evaporated before being re-dissolved in 800 µL n-heptane for injection.

4. GC-MS Instrumental Parameters

Analytical separation and detection were conducted under the following optimized settings:

**Parameter**	**Setting**
Column	DB-5ms (30 m, 0.25 mm ID, 0.25 µm film)
Carrier Gas	Helium at 0.8 mL/min (Constant flow)
Injection	2 µL, Pulsed Splitless, 250°C
Oven Program	80°C (1 min), increased at 10°C/min to 170°C, then at 3°C/min to 200°C, and finally at 15°C/min to 300°C (held for 15 min).

**Parameter**	**Setting**
MS Temperatures	Transfer Line: 300 °C<br>Source: 250 °C;<br>Quadrupole: 150 °C

Selected Ion Monitoring (SIM) Ions (m/z):

- 3-MCPD: 147 (Quant), 196, 198 (Qual)
- 3-MCPD-d5: 150 (Quant), 201 (Qual)
- 2-MCPD: 196 (Quant), 198 (Qual)
- 2-MCPD-d5: 201 (Quant), 203 (Qual)
- 3-MBPD (Glycidol): 147 (Quant), 240 (Qual)
