# Supplementary material for: Hidden Hazards: Assessment of Exposure Risks from 3-Monochloropropane-1,2-diol Ester (3-MCPDE) and Glycidyl Ester (GE) Consumption Among Malaysian Consumers
Source: Toxics. 2026 Apr 16;14(4):331. doi: 10.3390/toxics14040331 (PMC13119714; doi:10.3390/toxics14040331)
Supplement: Supplementary file 1 [file toxics-14-00331-s001.zip › Supplementary S2_Dietary exposure, MOE.pdf]

TOTAL POPULATION (3-MCPDE)

FOOD ITEM	Representative Occurrence level (mg/kg)	Intake Mean Consumer (kg/day)	Dietary exposure (µg/kg BW/day) Mean consumer	% PMTDI	% of total DE	Mean consumer MOE ($BMDL_{10}$/EDI)	Intake High Consumer P95 (kg/day)	Dietary exposure (µg/kg BW/day) High consumer P95	% PMTDI	% of total DE	High consumer MOE ($BMDL_{10}$/EDI)
**VEGETABLE FATS AND OILS**	**0.83**	**0.017**	**0.2305**	**5.76**	**4.13**		**0.092**	**1.2200**	**30.50**	**6.43**	
Margarine, Shortening	3.02	0.001	0.0497	1.24		4028	0.006	0.2671	5.54		749
Mayonnaise	0.14	0.000	0.0011	0.03		182122	0.002	0.0051	0.13		39353
Evaporated creamer	0.19	0.001	0.0039	0.10		50729	0.009	0.0259	0.65		7722
Concentrated Creamer	0.76	0.015	0.1761	4.40		1136	0.076	0.9173	22.93		218
**MILK AND DAIRY**	**0.30**	**0.003**	**0.0133**	**0.33**	**0.24**		**0.017**	**0.0800**	**2.00**	**0.42**	
Evaporated Milk	0.21	0.001	0.0044	0.11		45897	0.009	0.0286	0.72		6987
Butter	0.38	0.001	0.0089	0.22		22361	0.008	0.0482	1.20		4152
**CONFECTIONARY**	**0.28**	**0.031**	**0.1364**	**3.41**	**2.44**		**0.131**	**0.5800**	**14.50**	**3.06**	
Flavoured biscuit	0.53	0.003	0.0290	0.72		6904	0.017	0.1443	3.61		1386
Plain biscuit	0.45	0.010	0.0738	1.85		2709	0.045	0.3201	8.00		625
Bun	0.11	0.012	0.0202	0.51		9894	0.044	0.0763	1.91		2621
Cake	0.21	0.002	0.0065	0.16		30756	0.010	0.0338	0.84		5925
Cheese tart, doughnut	0.28	0.000	0.0018	0.05		109146	0.002	0.0073	0.10		27434
Chocolate bar	0.07	0.002	0.0027	0.07		73967	0.011	0.0127	0.32		15716
Chocolate spread	0.52	0.000	0.0020	0.05		100449	0.001	0.0106	0.27		18834
**SNACKS**	**0.46**	**0.037**	**0.2711**	**6.78**	**4.86**		**0.171**	**1.2600**	**31.50**	**6.64**	
Fried fish sausage (Keropok lekor)	0.11	0.003	0.0049	0.12		40828	0.011	0.0200	0.50		10001
Fried Fish Crackers (Keropok Ikan)	0.59	0.002	0.0178	0.44		11237	0.009	0.0858	2.14		2331
Murukku (Indian savoury crackers)<br>Potato chips<br>Chicken-flavoured snack<br>Seafood-flavoured snack<br>Fruit/vegetable-flavoured snack	0.49	0.032	0.2522	6.30		793	0.150	1.1600	22.91		172
**LOCAL KUIH-MUIH**	**0.64**	**0.023**	**0.2301**	**5.75**	**4.12**		**0.090**	**0.9200**	**23.00**	**4.85**	
Kuih denderam<br>Prawn fritter<br>Curry puff<br>Cakoi<br>Vadai<br>Banana fritters<br>Fried spring rolls<br>Fried cempedak<br>Fried sweet potato<br>Fried banana balls	0.64	0.023	0.2301	5.75		869	0.090	0.9200	22.91		217
**COOKED FOOD (FRIED)**	**0.56**	**0.489**	**4.3686**	**109.22**	**78.29**		**1.563**	**13.5300**	**338.25**	**71.29**	
Fried rice	0.72	0.275	3.1608	79.02		63	0.720	8.2745	206.86		24
Char-kuey-teow, fried rice noodle	0.50	0.065	0.5215	13.04		384	0.211	1.6873	42.18		119
Fried wheat noodle	0.23	0.065	0.2368	5.92		844	0.246	0.9031	22.58		221
Fried Indian Mackerel	0.24	0.047	0.1794	4.48		1115	0.128	0.4903	12.26		408
Indian flatbread (Roti canai), beef *martabak*	0.45	0.014	0.1014	2.54		1972	0.053	0.3635	9.09		550
Fried anchovies	1.67	0.004	0.1008	2.52		1985	0.018	0.4798	12.00		417
Butter prawn	1.05	0.002	0.0335	0.84		5967	0.009	0.1431	3.58		1397
Fried beef<br>Bergedil daging<br>Beef biryani<br>Beef satay	0.49	0.005	0.0418	1.04		4789	0.130	0.2290	5.73		873
Fried chicken	0.11	0.005	0.0080	0.20		24925	0.017	0.0295	0.74		6780
Fried macaroni	0.11	0.004	0.0075	0.19		26677	0.017	0.0305	0.76		6558
Fish ball	0.24	0.003	0.0113	0.28		17638	0.014	0.0546	1.36		3666
**FAST FOOD**	**0.23**	**0.015**	**0.0540**	**1.35**	**0.97**		**0.061**	**0.2200**	**5.50**	**1.16**	
Beef burger patty	0.30	0.007	0.0351	0.88		5706	0.022	0.1064	2.66		1881
Potato fries	0.16	0.002	0.0061	0.15		33043	0.011	0.0283	0.71		7055
Frankfurter/sausage/hotdog	0.17	0.002	0.0052	0.13		38589	0.015	0.0382	0.96		5230
Nugget	0.20	0.001	0.0043	0.11		46066	0.006	0.0189	0.47		10601
Pizza with beef and onion	0.11	0.002	0.0031	0.08		65465	0.008	0.0133	0.33		15008
**COOKED FOOD (STEWED/BOILED)**	**0.18**	**0.096**	**0.2762**	**6.90**	**4.95**		**0.999**	**1.1700**	**29.25**	**6.16**	
Instant noodles	0.18	0.065	0.1853	4.63		1079	0.246	0.7068	17.67		283
Beef in soy sauce/curry	0.54	0.005	0.0460	1.15		4345	0.017	0.1459	3.65		1371
Chicken *soto*	0.11	0.022	0.0388	0.97		5161	0.720	1.2642	31.60		158
Satay sauce	0.11	0.004	0.0074	0.18		27121	0.016	0.0281	0.70		7119
**TOTAL (by category)**		**0.71**	**5.58**	**139.50**			**3.12**	**18.98**	**474.50**		

* values reported for food category represent the consumption-weighted average concentration of constituent food items

*food mapping conducted for similar food items with a single exposure value (using a representative mean concentration)

MALE ADULTS (3-MCPDE)

FOOD ITEM	Representative Occurrence level (mg/kg)	Intake Mean Consumer (kg/day)	Dietary exposure (μg/kg BW/day) Mean consumer	% PMTDI	% of total DE	Mean consumer MOE ($BMDL_{10}$/EDI)	Intake High Consumer P95 (kg/day)	Dietary exposure (μg/kg BW/day) High consumer P95	% PMTDI	% of total DE	High consumer MOE ($BMDL_{10}$/EDI)
**VEGETABLE FATS AND OILS**	**0.82**	**0.021**	**0.2590**	**6.47**	**3.93**		**0.095**	**1.1752**	**29.38**	**4.05**	
Margarine, Shortening	3.02	0.001	0.0495	1.24		4044	0.007	0.3358	8.39		596
Mayonnaise	0.14	0.001	0.0012	0.03		168863	0.002	0.0043	0.11		46671
Evaporated creamer	0.19	0.002	0.0045	0.11		44626	0.010	0.0285	0.71		7006
Concentrated Creamer	0.76	0.018	0.2023	5.06		988	0.076	0.8634	21.59		232
**MILK AND DAIRY**	**0.30**	**0.003**	**0.0145**	**0.36**	**0.22**		**0.018**	**0.0810**	**2.02**	**0.28**	
Evaporated Milk	0.21	0.002	0.0050	0.12		40376	0.010	0.0316	0.79		6339
Butter	0.38	0.002	0.0093	0.23		21439	0.008	0.0453	1.13		4412
**CONFECTIONARY**	**0.26**	**0.033**	**0.1271**	**3.18**	**1.93**		**0.127**	**0.4975**	**12.44**	**1.71**	
Flavoured biscuit	0.53	0.004	0.0307	0.77		6518	0.017	0.1358	3.39		1473
Plain biscuit	0.45	0.009	0.0615	1.54		3254	0.038	0.2574	6.43		777
Bun	0.11	0.015	0.0247	0.62		8090	0.053	0.0862	2.15		2320
Cake	0.21	0.002	0.0061	0.29		32676	0.007	0.0236	1.12		8463
Cheese tart, doughnut	0.28	0.000	0.0000	0.00		0	0.000	0.0000	0.00		0
Chocolate bar	0.07	0.002	0.0025	0.08		80241	0.011	0.0120	0.39		16696
Chocolate spread	0.52	0.000	0.0000	0.00		0	0.000	0.0000	0.00		0
**SNACKS**	**0.46**	**0.035**	**0.2392**	**5.98**	**3.63**		**1.516**	**10.4805**	**262.01**	**36.12**	
Fried fish sausage (Keropok lekor)	0.11	0.003	0.0056	0.16		35804	0.014	0.0235	0.68		8498
Fried Fish Crackers (Keropok Ikan)	0.59	0.002	0.0177	0.56		11281	0.010	0.0909	2.86		2201
Murukku (Indian savoury crackers)<br>Potato chips<br>Chicken-flavoured snack<br>Seafood-flavoured snack<br>Fruit/vegetable-flavoured snack	0.49	0.029	0.2152	5.38		929	0.142	1.0454	26.13		191
**LOCAL KUIH-MUIH**	**0.64**	**0.024**	**0.2325**	**5.81**	**3.53**		**0.090**	**0.8654**	**21.63**	**2.98**	
Kuih denderam<br>Prawn fritter<br>Curry puff<br>Cakoi<br>Vadai<br>Banana fritters<br>Fried spring rolls<br>Fried cempedak<br>Fried sweet potato<br>Fried banana balls	0.64	0.024	0.2325	5.81		860	0.0900	0.8654	36.62		231
**COOKED FOOD (FRIED)**	**0.57**	**0.566**	**4.8451**	**121.13**	**73.54**		**1.592**	**13.6326**	**340.82**	**46.98**	
Fried rice	0.72	0.325	3.5128	80.50		57	0.720	7.7885	178.49		26
Char-kuey-teow, fried rice noodle	0.50	0.069	0.5177	12.94		386	0.282	2.1176	52.94		94
Fried wheat noodle	0.23	0.079	0.2716	7.09		736	0.288	0.9952	25.96		201
Fried Indian Mackerel	0.24	0.048	0.1715	4.05		1166	0.137	0.4928	11.64		406
Indian flatbread (Roti canai), beef *martabak*	0.45	0.020	0.1365	3.41		1465	0.066	0.4476	11.19		447
Fried anchovies	1.67	0.004	0.0968	2.32		2065	0.018	0.4501	10.80		444
Butter prawn	1.05	0.002	0.0323	1.83		6184	0.009	0.1347	7.62		1485
Fried beef<br>Bergedil daging<br>Beef biryani<br>Beef satay	0.49	0.007	0.0520	1.30		3843	0.026	0.1885	7.00		1061
Fried chicken	0.11	0.006	0.0095	0.52		21120	0.017	0.0278	1.54		7203
Fried macaroni	0.11	0.004	0.0064	0.33		31190	0.016	0.0265	1.36		7549
Fish ball	0.24	0.003	0.0114	0.30		17497	0.014	0.0513	1.34		3895
**FAST FOOD**	**0.23**	**0.018**	**0.0612**	**1.53**	**0.93**		**0.066**	**0.2278**	**5.69**	**0.78**	
Beef burger patty	0.30	0.010	0.0430	1.17		4646	0.026	0.1155	3.14		1731
Potato fries	0.16	0.003	0.0063	0.24		32000	0.011	0.0267	1.01		7495
Frankfurter/sausage/hotdog	0.17	0.002	0.0053	0.13		37467	0.015	0.0370	0.93		5400
Nugget	0.20	0.002	0.0045	0.11		44079	0.007	0.0213	0.52		9375
Pizza with beef and onion	0.11	0.002	0.0032	0.08		61744	0.008	0.0125	0.31		15944
**COOKED FOOD (STEWED/BOILED)**	**0.13**	**0.415**	**0.8098**	**20.25**	**12.29**		**1.054**	**2.0576**	**51.44**	**7.09**	
Instant noodles	0.18	0.079	0.2126	8.17		941	0.288	0.7788	29.93		257
Beef in soy sauce/curry	0.54	0.007	0.0574	1.43		3487	0.025	0.2028	5.07		986
Chicken *soto*	0.11	0.325	0.5367	13.42		373	0.720	1.1899	29.75		168
Satay sauce	0.11	0.004	0.0070	0.17		28677	0.021	0.0339	0.85		5903
**TOTAL (by category)**			**6.59**	**164.71**				**29.02**	**725.44**		

FEMALE ADULTS (3-MCPDE)

FOOD ITEM	Representative Occurrence level (mg/kg)	Intake Mean Consumer (kg/day)	Dietary exposure (µg/kg BW/day) Mean consumer	% PMTDI	% of total DE	Mean consumer MOE ($BMDL_{10}$/EDI)	Intake High Consumer P95 (kg/day)	Dietary exposure (µg/kg BW/day) High consumer P95	% PMTDI	% of total DE	High consumer MOE ($BMDL_{10}$/EDI)
**VEGETABLE FATS AND OILS**	**0.86**	**0.013**	**0.1976**	**4.94**	**3.65**		**0.057**	**0.8316**	**20.79**	**4.84**	
Margarine, Shortening	3.02	0.001	0.0496	1.24		4031	0.006	0.2868	7.17		697
Mayonnaise	0.14	0.000	0.0010	0.02		203860	0.002	0.0049	0.12		40977
Evaporated creamer	0.19	0.001	0.0033	0.08		61516	0.006	0.0185	0.46		10811
Concentrated Creamer	0.76	0.011	0.1432	3.58		1396	0.043	0.5600	14.00		357
**MILK AND DAIRY**	**0.31**	**0.002**	**0.0121**	**0.30**	**0.22**		**0.013**	**0.0673**	**1.68**	**0.39**	
Evaporated Milk	0.21	0.001	0.0036	0.09		55657	0.006	0.0204	0.51		9782
Butter	0.38	0.001	0.0084	0.21		23931	0.007	0.0454	1.13		4410
**CONFECTIONARY**	**0.31**	**0.028**	**0.1460**	**3.65**	**2.69**		**0.125**	**0.6626**	**16.57**	**3.86**	
Flavoured biscuit	0.53	0.003	0.0269	0.67		7437	0.021	0.1856	4.64		1078
Plain biscuit	0.45	0.012	0.0892	2.23		2242	0.045	0.3432	8.58		583
Bun	0.11	0.008	0.0146	0.36		13744	0.035	0.0655	1.64		3055
Cake	0.21	0.002	0.0070	0.17		28689	0.011	0.0389	0.97		5144
Cheese tart, doughnut	0.28	0.000	0.0017	0.04		119265	0.002	0.0079	0.20		25453
Chocolate bar	0.07	0.002	0.0030	0.07		67057	0.011	0.0136	0.34		14659
Chocolate spread	0.52	0.000	0.0020	0.05		102217	0.000	0.0023	0.06		86491
**SNACKS**	**0.47**	**0.039**	**0.3174**	**7.93**	**5.85**		**0.204**	**1.6373**	**40.93**	**9.53**	
Fried fish sausage (Keropok lekor)	0.11	0.002	0.0040	0.10		49652	0.009	0.0161	0.40		12442
Fried Fish Crackers (Keropok Ikan)	0.59	0.002	0.0179	0.45		11192	0.009	0.0862	2.16		2320
Murukku (Indian savoury crackers)<br>Potato chips<br>Chicken-flavoured snack<br>Seafood-flavoured snack<br>Fruit/vegetable-flavoured snack	0.49	0.036	0.2981	7.45		671	0.187	1.5637	39.09		128
**LOCAL KUIH-MUIH**	**0.64**	**0.021**	**0.2116**	**5.29**	**3.90**		**0.090**	**0.9856**	**24.64**	**5.74**	
Kuih denderam<br>Prawn fritter<br>Curry puff<br>Cakoi<br>Vadai<br>Banana fritters<br>Fried spring rolls<br>Fried cempedak<br>Fried sweet potato<br>Fried banana balls	0.64	0.021	0.2268	5.67		882	0.090	0.9856	24.64		203
**COOKED FOOD (FRIED)**	**0.56**	**0.405**	**3.8770**	**96.92**	**71.51**		**1.159**	**11.1103**	**277.76**	**64.68**	
Fried rice	0.72	0.221	2.7179	67.95		74	0.540	6.6530	166.32		30
Char-kuey-teow, fried rice noodle	0.50	0.061	0.5259	13.15		380	0.188	1.6079	40.20		124
Fried wheat noodle	0.23	0.049	0.1937	4.84		1032	0.185	0.7262	18.15		275
Fried Indian Mackerel	0.24	0.046	0.1890	4.72		1058	0.128	0.5257	13.14		380
Indian flatbread (Roti canai), beef *martabak*	0.45	0.007	0.0574	1.44		3482	0.026	0.2039	5.10		981
Fried anchovies	1.67	0.004	0.1054	2.64		1897	0.018	0.5144	12.86		389
Butter prawn	1.05	0.002	0.0349	0.87		5738	0.008	0.1446	3.62		1383
Fried beef<br>Bergedil daging<br>Beef biryani<br>Beef satay	0.49	0.003	0.0289	0.72		6914	0.013	0.1074	2.69		1862
Fried chicken	0.11	0.003	0.0062	0.16		32101	0.017	0.0316	0.79		6325
Fried macaroni	0.11	0.005	0.0088	0.22		22607	0.024	0.0453	1.13		4418
Fish ball	0.24	0.003	0.0112	0.28		17839	0.013	0.0526	1.32		3802
**FAST FOOD**	**0.22**	**0.011**	**0.0430**	**1.08**	**0.79**		**0.055**	**0.2072**	**5.18**	**1.21**	
Beef burger patty	0.30	0.005	0.0253	0.63		7919	0.022	0.1140	2.85		1754
Potato fries	0.16	0.002	0.0058	0.15		34458	0.010	0.0281	0.70		7127
Frankfurter/sausage/hotdog	0.17	0.002	0.0049	0.12		40443	0.010	0.0282	0.70		7103
Nugget	0.20	0.001	0.0041	0.10		48700	0.005	0.0182	0.46		10985
Pizza with beef and onion	0.11	0.001	0.0028	0.07		71312	0.008	0.0143	0.36		13999
**COOKED FOOD (STEWED/BOILED)**	**0.13**	**0.277**	**0.6171**	**15.43**	**11.38**		**0.753**	**1.6758**	**41.89**	**9.76**	
Instant noodles	0.18	0.049	0.1516	3.79		1319	0.185	0.5683	14.21		352
Beef in soy sauce/curry	0.54	0.003	0.0319	0.80		6274	0.013	0.1184	2.96		1690
Chicken *soto*	0.11	0.221	0.4152	10.38		482	0.540	1.0164	25.41		197
Satay sauce	0.11	0.004	0.0078	0.20		25542	0.016	0.0301	0.75		6641
**TOTAL (by category)**			**5.42**	**135.55**				**17.18**	**429.44**		

MALAY (3-MCPDE)

FOOD ITEM	Representative Occurrence level (mg/kg)	Intake Mean Consumer (kg/day)	Dietary exposure (µg/kg BW/day) Mean consumer	% PMTDI	% of total DE	Mean consumer MOE ($BMDL_{10}$/EDI)	Intake High Consumer P95 (kg/day)	Dietary exposure (µg/kg BW/day) High consumer P95	% PMTDI	% of total DE	High consumer MOE ($BMDL_{10}$/EDI)
**VEGETABLE FATS AND OILS**	**0.82**	**0.022**	**0.2865**	**7.16**	**4.81**		**0.096**	**1.2564**	**31.41**	**7.90**	
Margarine, Shortening	3.02	0.001	0.0569	1.42		3516	0.007	0.3374	8.44		28571
Mayonnaise	0.14	0.001	0.0014	0.04		140998	0.003	0.0068	0.17		66890
Evaporated creamer	0.19	0.002	0.0049	0.12		41217	0.010	0.0303	0.76		20000
Concentrated Creamer	0.76	0.018	0.2232	5.58		896	0.076	0.9173	22.93		2632
**MILK AND DAIRY**	**0.28**	**0.003**	**0.0123**	**0.31**	**0.21**		**0.016**	**0.0715**	**1.79**	**0.45**	
Evaporated Milk	0.21	0.002	0.0054	0.13		37292	0.010	0.0335	0.84		20000
Butter	0.38	0.001	0.0070	0.18		28530	0.006	0.0363	0.91		33333
**CONFECTIONARY**	**0.29**	**0.029**	**0.1350**	**3.37**	**2.26**		**0.138**	**0.6381**	**15.95**	**4.01**	
Flavoured biscuit	0.53	0.004	0.0339	0.85		5905	0.021	0.1731	4.33		9756
Plain biscuit	0.45	0.011	0.0792	1.98		2524	0.045	0.3200	8.00		4445
Bun	0.11	0.012	0.0202	0.50		9911	0.053	0.0916	2.29		3776
Cake	0.21	0.001	0.0049	0.12		40590	0.005	0.0181	0.45		36969
Cheese tart, doughnut	0.28	0.000	0.0013	0.03		154310	0.002	0.0089	0.22		100000
Chocolate bar	0.07	0.000	0.0003	0.01		617241	0.011	0.0123	0.31		18182
Chocolate spread	0.52	0.000	0.0024	0.06		83130	0.001	0.0083	0.21		200000
**SNACKS**	**0.46**	**0.048**	**0.3545**	**8.86**	**5.95**		**0.246**	**1.8062**	**45.16**	**11.36**	
Fried fish sausage (Keropok lekor)	0.11	0.004	0.0078	0.20		896	0.020	0.0351	0.88		10000
Fried Fish Crackers (Keropok Ikan)	0.59	0.003	0.0265	0.66		896	0.012	0.1130	2.83		16667
Murukku (Indian savoury crackers)<br>Potato chips<br>Chicken-flavoured snack<br>Seafood-flavoured snack<br>Fruit/vegetable-flavoured snack	0.49	0.041	0.3207	8.02		896	0.214	1.6737	41.84		935
**LOCAL KUIH-MUIH**	**0.64**	**0.028**	**0.2860**	**7.15**	**4.80**		**0.090**	**0.9194**	**22.98**	**5.78**	
Kuih denderam<br>Prawn fritter<br>Curry puff<br>Cakoi<br>Vadai<br>Banana fritters<br>Fried spring rolls<br>Fried cempedak<br>Fried sweet potato<br>Fried banana balls	0.64	0.028	0.2860	7.15		699	0.090	0.9194	22.98		2222
**COOKED FOOD (FRIED)**	**0.57**	**0.462**	**4.2030**	**105.07**	**70.50**		**1.113**	**10.1263**	**253.16**	**63.68**	
Fried rice	0.72	0.264	3.0295	75.74		66	0.480	5.5164	137.91		417
Char-kuey-teow, fried rice noodle	0.50	0.055	0.4354	10.88		459	0.188	1.5004	37.51		1064
Fried wheat noodle	0.23	0.045	0.1649	4.12		1213	0.164	0.6021	15.05		1220
Fried Indian Mackerel	0.24	0.054	0.2060	5.15		971	0.128	0.4903	12.26		1563
Indian flatbread (Roti canai), beef *martabak*	0.45	0.019	0.1383	3.46		1446	0.053	0.3807	9.52		3774
Fried anchovies	1.67	0.004	0.1101	2.75		1817	0.018	0.4798	12.00		11111
Butter prawn	1.05	0.002	0.0364	0.91		5499	0.009	0.1508	3.77		22222
Fried beef<br>Bergedil daging<br>Beef biryani<br>Beef satay	0.49	0.008	0.0626	1.56		3196	0.026	0.2034	5.08		7692
Fried chicken	0.11	0.005	0.0091	0.23		21864	0.017	0.0298	0.75		11765
Fried macaroni	0.11	0.004	0.0072	0.18		27783	0.017	0.0298	0.75		11765
Fish ball	0.24	0.002	0.0085	0.21		23412	0.013	0.0498	1.25		15385
**FAST FOOD**	**0.16**	**0.007**	**0.0179**	**0.45**	**0.30**		**0.036**	**0.0919**	**2.30**	**0.58**	
Beef burger patty	0.30	0.000	0.0000	0.00		0	0.000	0.0000	0.00		0.00
Potato fries	0.16	0.002	0.0051	0.13		39156	0.011	0.0281	0.70		18182
Frankfurter/sausage/hotdog	0.17	0.001	0.0027	0.07		73706	0.010	0.0271	0.68		20000
Nugget	0.20	0.002	0.0064	0.16		31325	0.007	0.0223	0.56		28571
Pizza with beef and onion	0.11	0.002	0.0035	0.09		56955	0.008	0.0140	0.35		25000
**COOKED FOOD (STEWED/BOILED)**	**0.13**	**0.321**	**0.6661**	**16.65**	**11.17**		**0.478**	**0.9919**	**24.80**	**6.24**	
Instant noodles	0.18	0.045	0.1293	3.23		1547	0.164	0.4712	11.78		1220
Beef in soy sauce/curry	0.54	0.008	0.0690	1.72		2900	0.026	0.2241	5.60		7692
Chicken *soto*	0.11	0.263	0.4618	11.54		433	0.264	0.4635	11.59		758
Satay sauce	0.11	0.005	0.0088	0.22		22782	0.024	0.0421	1.05		8333
**TOTAL (by category)**			**5.96**	**149.03**				**15.90**	**397.54**		

CHINESE (3-MCPDE)

FOOD ITEM	Representative Occurrence level (mg/kg)	Intake Mean Consumer (kg/day)	Dietary exposure (µg/kg BW/day) Mean consumer	% PMTDI	% of total DE	Mean consumer MOE ($BMDL_{10}$/EDI)	Intake High Consumer P95 (kg/day)	Dietary exposure (µg/kg BW/day) High consumer P95	% PMTDI	% of total DE	High consumer MOE ($BMDL_{10}$/EDI)
**VEGETABLE FATS AND OILS**	**3.01**	**0.001**	**0.0481**	**1.20**	**0.96**		**0.006**	**0.2887**	**7.22**	**2.00**	
Margarine, Shortening	3.02	0.001	0.0482	1.21		4149	0.006	0.2892	7.23		692
Mayonnaise	0.14	0.000	0.0000	0.00		0	0.000	0.0000	0.00		0
Evaporated creamer	0.19	0.000	0.0000	0.00		0	0.000	0.0000	0.00		0
Concentrated Creamer	0.76	0.000	0.0000	0.00		0	0.000	0.0000	0.00		0
**MILK AND DAIRY**	**0.38**	**0.003**	**0.0184**	**0.46**	**0.37**		**0.012**	**0.0726**	**1.81**	**0.50**	
Evaporated Milk	0.21	0.001	0.0035	0.09		57372	0.000	0.0000	0.00		0
Butter	0.38	0.002	0.0121	0.30		16547	0.012	0.0725	1.81		2758
**CONFECTIONARY**	**0.25**	**0.021**	**0.0835**	**2.09**	**1.67**		**0.105**	**0.4173**	**10.43**	**2.88**	
Flavoured biscuit	0.53	0.002	0.0169	0.42		11840	0.010	0.0845	2.11		2368
Plain biscuit	0.45	0.006	0.0427	1.07		4686	0.036	0.2561	6.40		781
Bun	0.11	0.009	0.0156	0.39		12851	0.035	0.0605	1.51		3305
Cake	0.21	0.002	0.0067	0.17		29833	0.011	0.0369	0.92		5424
Cheese tart, doughnut	0.28	0.000	0.0000	0.00		0	0.002	0.0089	0.22		22375
Chocolate bar	0.07	0.002	0.0022	0.06		89500	0.011	0.0123	0.31		16273
Chocolate spread	0.52	0.000	0.0000	0.00		0	0.000	0.0000	0.00		0
**SNACKS**	**0.47**	**0.012**	**0.0902**	**2.26**	**1.80**		**0.078**	**0.5864**	**14.66**	**4.05**	
Fried fish sausage (Keropok lekor)	0.11	0.000	0.0000	0.00		0	0.002	0.0035	0.09		56955
Fried Fish Crackers (Keropok Ikan)	0.59	0.000	0.0000	0.00		0	0.000	0.0000	0.00		0
Murukku (Indian savoury crackers)<br>Potato chips<br>Chicken-flavoured snack<br>Seafood-flavoured snack<br>Fruit/vegetable-flavoured snack	0.49	0.012	0.0939	2.35		2131	0.076	0.5944	14.86		336
**LOCAL KUIH-MUIH**	**0.64**	**0.007**	**0.0713**	**1.78**	**1.42**		**0.034**	**0.3462**	**8.66**	**2.39**	
Kuih denderam<br>Prawn fritter<br>Curry puff<br>Cakoi<br>Vadai<br>Banana fritters<br>Fried spring rolls<br>Fried cempedak<br>Fried sweet potato<br>Fried banana balls	0.64	0.007	0.0715	1.79		2797	0.034	0.3473	8.68		576
**COOKED FOOD (FRIED)**	**0.53**	**0.472**	**3.9779**	**99.45**	**79.47**		**1.293**	**10.8971**	**272.43**	**75.32**	
Fried rice	0.72	0.220	2.5283	63.21		79	0.489	5.6198	140.49		36
Char-kuey-teow, fried rice noodle	0.50	0.102	0.8140	20.35		246	0.330	2.6337	65.84		76
Fried wheat noodle	0.23	0.096	0.3524	8.81		567	0.288	1.0573	26.43		189
Fried Indian Mackerel	0.24	0.026	0.0996	2.49		2008	0.064	0.2452	6.13		816
Indian flatbread (Roti canai), beef *martabak*	0.45	0.007	0.0503	1.26		3978	0.026	0.1868	4.67		1071
Fried anchovies	1.67	0.002	0.0533	1.33		3751	0.009	0.2399	6.00		834
Butter prawn	1.05	0.002	0.0335	0.84		5967	0.009	0.1508	3.77		1326
Fried beef<br>Bergedil daging<br>Beef biryani<br>Beef satay	0.49	0.002	0.0156	0.39		12786	0.009	0.0704	1.76		2841
Fried chicken	0.11	0.004	0.0070	0.18		28477	0.017	0.0298	0.75		6701
Fried macaroni	0.11	0.006	0.0105	0.26		18985	0.035	0.0615	1.54		3255
Fish ball	0.24	0.005	0.0192	0.48		10442	0.017	0.0651	1.63		3071
**FAST FOOD**	**0.23**	**0.010**	**0.0370**	**0.93**	**0.74**		**0.047**	**0.1740**	**4.35**	**1.20**	
Beef burger patty	0.30	0.006	0.0287	0.72		6961	0.022	0.1053	2.63		1898
Potato fries	0.16	0.002	0.0051	0.13		39156	0.011	0.0281	0.70		7119
Frankfurter/sausage/hotdog	0.17	0.001	0.0027	0.07		73706	0.004	0.0109	0.27		18426
Nugget	0.20	0.000	0.0000	0.00		0	0.002	0.0064	0.16		31325
Pizza with beef and onion	0.11	0.001	0.0018	0.04		113909	0.008	0.0140	0.35		14239
**COOKED FOOD (STEWED/BOILED)**	**0.13**	**0.320**	**0.6793**	**16.98**	**13.57**		**0.794**	**1.6856**	**42.14**	**11.65**	
Instant noodles	0.18	0.096	0.2758	6.90		725	0.288	0.8275	20.69		242
Beef in soy sauce/curry	0.54	0.002	0.0172	0.43		11602	0.009	0.0776	1.94		2578
Chicken *soto*	0.11	0.220	0.3863	9.66		518	0.489	0.8586	21.46		233
Satay sauce	0.11	0.002	0.0035	0.09		56955	0.008	0.0140	0.35		14239
**TOTAL (by category)**			**5.01**	**125.14**				**14.47**	**361.70**		

INDIAN (3-MCPDE)

FOOD ITEM	Representative Occurrence level (mg/kg)	Intake Mean Consumer (kg/day)	Dietary exposure (µg/kg BW/day) Mean consumer	% PMTDI	% of total DE	Mean consumer MOE ($BMDL_{10}$/EDI)	Intake High Consumer P95 (kg/day)	Dietary exposure (µg/kg BW/day) High consumer P95	% PMTDI	% of total DE	High consumer MOE ($BMDL_{10}$/EDI)
**VEGETABLE FATS AND OILS**	**3.27**	**0.002**	**0.1044**	**2.61**	**2.27**		**0.007**	**0.3655**	**9.14**	**2.62**	
Margarine, Shortening	3.02	0.001	0.0482	1.21		200000	0.007	0.3374	8.44		28571
Mayonnaise	0.14	0.000	0.0000	0.00		0	0.000	0.0000	0.00		0
Evaporated creamer	0.19	0.001	0.0030	0.08		200000	0.000	0.0000	0.00		0
Concentrated Creamer	0.76	0.000	0.0000	0.00		0	0.000	0.0000	0.00		0
**MILK AND DAIRY**	**0.00**	**0.000**	**0.0000**	**0.00**	**0.00**		**0.000**	**0.0000**	**0.00**	**0.00**	
Evaporated Milk	0.21	0.000	0.0000	0.00		0	0.000	0.0000	0.00		0
Butter	0.38	0.000	0.0000	0.00		0	0.000	0.0000	0.00		0
**CONFECTIONARY**	**0.42**	**0.017**	**0.1126**	**2.82**	**2.45**		**0.065**	**0.4306**	**10.76**	**3.09**	
Flavoured biscuit	0.53	0.000	0.0000	0.00		0	0.000	0.0000	0.00		0
Plain biscuit	0.45	0.015	0.1067	2.67		13333	0.054	0.3841	9.60		3704
Bun	0.11	0.000	0.0000	0.00		0	0.000	0.0000	0.00		0
Cake	0.21	0.002	0.0067	0.17		100000	0.011	0.0369	0.92		18182
Cheese tart, doughnut	0.28	0.000	0.0000	0.00		0	0.000	0.0000	0.00		0
Chocolate bar	0.07	0.000	0.0000	0.00		0	0.000	0.0000	0.00		0
Chocolate spread	0.52	0.000	0.0000	0.00		0	0.000	0.0000	0.00		0
**SNACKS**	**0.59**	**0.000**	**0.0000**	**0.00**	**0.00**		**0.002**	**0.0188**	**0.47**	**0.14**	
Fried fish sausage (Keropok lekor)	0.11	0.000	0.0000	0.00		0	0.000	0.0000	0.00		0
Fried Fish Crackers (Keropok Ikan)	0.59	0.000	0.0000	0.00		0	0.002	0.0188	0.47		100000
Murukku (Indian savoury crackers)<br>Potato chips<br>Chicken-flavoured snack<br>Seafood-flavoured snack<br>Fruit/vegetable-flavoured snack	0.49	0.000	0.0000	0.00		0	0.000	0.0000	0.00		0
**LOCAL KUIH-MUIH**	**0.64**	**0.021**	**0.2145**	**5.36**	**4.66**		**0.085**	**0.8683**	**21.71**	**6.23**	
Kuih denderam<br>Prawn fritter<br>Curry puff<br>Cakoi<br>Vadai<br>Banana fritters<br>Fried spring rolls<br>Fried cempedak<br>Fried sweet potato<br>Fried banana balls	0.64	0.021	0.2145	5.36		9524	0.085	0.8683	21.71		2353
**COOKED FOOD (FRIED)**	**0.56**	**0.403**	**3.6022**	**90.06**	**78.27**		**1.193**	**10.6637**	**266.59**	**76.56**	
Fried rice	0.72	0.213	2.4479	61.20		939	0.480	5.5164	137.91		417
Char-kuey-teow, fried rice noodle	0.50	0.073	0.5826	14.57		2740	0.251	2.0032	50.08		797
Fried wheat noodle	0.23	0.058	0.2129	5.32		3448	0.238	0.8737	21.84		840
Fried Indian Mackerel	0.24	0.035	0.1341	3.35		5714	0.128	0.4903	12.26		1563
Indian flatbread (Roti canai), beef *martabak*	0.45	0.014	0.1006	2.51		14286	0.053	0.3807	9.52		3774
Fried anchovies	1.67	0.004	0.1066	2.67		50000	0.013	0.3465	8.66		15385
Butter prawn	1.05	0.001	0.0168	0.42		200000	0.005	0.0838	2.09		40000
Fried beef<br>Bergedil daging<br>Beef biryani<br>Beef satay	0.49	0.000	0.0000	0.00		0	0.000	0.0000	0.00		0
Fried chicken	0.11	0.002	0.0035	0.09		100000	0.012	0.0211	0.53		16667
Fried macaroni	0.11	0.000	0.0000	0.00		0	0.000	0.0000	0.00		0
Fish ball	0.24	0.003	0.0115	0.29		66667	0.013	0.0498	1.25		15385
**FAST FOOD**	**0.25**	**0.007**	**0.0280**	**0.70**	**0.61**		**0.037**	**0.1482**	**3.71**	**1.06**	
Beef burger patty	0.30	0.004	0.0192	0.48		50000	0.022	0.1053	2.63		9091
Potato fries	0.16	0.002	0.0051	0.13		100000	0.008	0.0204	0.51		25000
Frankfurter/sausage/hotdog	0.17	0.000	0.0000	0.00		0	0.000	0.0000	0.00		0
Nugget	0.20	0.001	0.0032	0.08		200000	0.007	0.0223	0.56		28571
Pizza with beef and onion	0.11	0.000	0.0000	0.00		0	0.000	0.0000	0.00		0
**COOKED FOOD (STEWED/BOILED)**	**0.13**	**0.271**	**0.5407**	**13.52**	**11.75**		**0.718**	**1.4326**	**35.81**	**10.29**	
Instant noodles	0.18	0.058	0.1666	4.17		3448	0.238	0.6838	17.09		840
Beef in soy sauce/curry	0.54	0.000	0.0000	0.00		0	0.000	0.0000	0.00		0
Chicken *soto*	0.11	0.213	0.3740	9.35		939	0.480	0.8428	21.07		417
Satay sauce	0.11	0.000	0.0000	0.00		0	0.000	0.0000	0.00		0
**TOTAL (by category)**			**4.60**	**115.06**				**13.93**	**348.19**		

OTHERS (bumiputras) (3-MCPDE)

FOOD ITEM	Representative Occurrence level (mg/kg)	Intake Mean Consumer (kg/day)	Dietary exposure (µg/kg BW/day) Mean consumer	% PMTDI	% of total DE	Mean consumer MOE ($BMDL_{10}$/EDI)	Intake High Consumer P95 (kg/day)	Dietary exposure (µg/kg BW/day) High consumer P95	% PMTDI	% of total DE	High consumer MOE ($BMDL_{10}$/EDI)
**VEGETABLE FATS AND OILS**	**0.68**	**0.010**	**0.1085**	**2.71**	**1.40**		**0.045**	**0.4851**	**12.13**	**2.27**	
Margarine, Shortening	3.02	0.000	0.0000	0.00		0	0.000	0.0000	0.00		0
Mayonnaise	0.14	0.000	0.0000	0.00		0	0.000	0.0000	0.00		0
Evaporated creamer	0.19	0.001	0.0030	0.08		200000	0.007	0.0203	0.51		29895
Concentrated Creamer	0.76	0.009	0.1086	2.72		22222	0.038	0.4587	11.47		5263
**MILK AND DAIRY**	**0.28**	**0.002**	**0.0094**	**0.23**	**0.12**		**0.013**	**0.0564**	**1.41**	**0.26**	
Evaporated Milk	0.21	0.001	0.0042	0.10		161290	0.007	0.0224	0.56		29895
Butter	0.38	0.001	0.0052	0.13		232558	0.006	0.0361	0.90		33445
**CONFECTIONARY**	**0.30**	**0.029**	**0.1390**	**3.48**	**1.79**		**0.123**	**0.5783**	**14.46**	**2.71**	
Flavoured biscuit	0.53	0.003	0.0270	0.68		62500	0.021	0.1731	4.33		9756
Plain biscuit	0.45	0.012	0.0879	2.20		16181	0.049	0.3500	8.75		4065
Bun	0.11	0.014	0.0241	0.60		14358	0.053	0.0916	2.29		3776
Cake	0.21	0.000	0.0000	0.00		0	0.000	0.0000	0.00		0
Cheese tart, doughnut	0.28	0.000	0.0000	0.00		0	0.000	0.0000	0.00		0
Chocolate bar	0.07	0.000	0.0000	0.00		0	0.000	0.0000	0.00		0
Chocolate spread	0.52	0.000	0.0000	0.00		0	0.000	0.0000	0.00		0
**SNACKS**	**0.48**	**0.039**	**0.2996**	**7.49**	**3.86**		**0.181**	**1.3746**	**34.36**	**6.43**	
Fried fish sausage (Keropok lekor)	0.11	0.001	0.0024	0.06		145985	0.006	0.0097	0.24		36364
Fried Fish Crackers (Keropok Ikan)	0.59	0.001	0.0105	0.26		178571	0.006	0.0536	1.34		35149
Murukku (Indian savoury crackers)<br>Potato chips<br>Chicken-flavoured snack<br>Seafood-flavoured snack<br>Fruit/vegetable-flavoured snack	0.49	0.037	0.2894	7.23		5405	0.170	1.3296	33.24		1176
**LOCAL KUIH-MUIH**	**0.64**	**0.026**	**0.2648**	**6.62**	**3.42**		**0.090**	**0.9165**	**22.91**	**4.29**	
Kuih denderam<br>Prawn fritter<br>Curry puff<br>Cakoi<br>Vadai<br>Banana fritters<br>Fried spring rolls<br>Fried cempedak<br>Fried sweet potato<br>Fried banana balls	0.64	0.026	0.2656	6.64		7692	0.090	0.9194	22.98		2222
**COOKED FOOD (FRIED)**	**0.57**	**0.638**	**5.8468**	**146.17**	**75.43**		**1.666**	**15.2715**	**381.79**	**71.49**	
Fried rice	0.72	0.405	4.6529	116.32		494	0.900	10.3432	258.58		222
Char-kuey-teow, fried rice noodle	0.50	0.059	0.4731	11.83		3374	0.211	1.6873	42.18		946
Fried wheat noodle	0.23	0.101	0.3699	9.25		1985	0.288	1.0573	26.43		694
Fried Indian Mackerel	0.24	0.055	0.2097	5.24		3654	0.192	0.7355	18.39		1042
Indian flatbread (Roti canai), beef *martabak*	0.45	0.006	0.0417	1.04		34483	0.026	0.1902	4.75		7553
Fried anchovies	1.67	0.000	0.0000	0.00		0	0.000	0.0000	0.00		0
Butter prawn	1.05	0.002	0.0297	0.74		112994	0.008	0.1289	3.22		26008
Fried beef<br>Bergedil daging<br>Beef biryani<br>Beef satay	0.49	0.003	0.0235	0.59		66667	0.010	0.0782	1.96		20000
Fried chicken	0.11	0.005	0.0081	0.20		43103	0.017	0.0306	0.77		11461
Fried macaroni	0.11	0.000	0.0000	0.00		0	0.000	0.0000	0.00		0
Fish ball	0.24	0.003	0.0115	0.29		66667	0.013	0.0498	1.25		15385
**FAST FOOD**	**0.21**	**0.014**	**0.0467**	**1.17**	**0.60**		**0.067**	**0.2271**	**5.68**	**1.06**	
Beef burger patty	0.30	0.005	0.0222	0.55		43197	0.022	0.1064	2.66		9005
Potato fries	0.16	0.002	0.0048	0.12		105820	0.008	0.0196	0.49		26008
Frankfurter/sausage/hotdog	0.17	0.006	0.0156	0.39		34722	0.029	0.0788	1.97		6887
Nugget	0.20	0.001	0.0045	0.11		140845	0.008	0.0245	0.61		26008
Pizza with beef and onion	0.11	0.000	0.0000	0.00		0	0.000	0.0000	0.00		0
**COOKED FOOD (STEWED/BOILED)**	**0.13**	**0.513**	**1.0370**	**25.92**	**13.38**		**1.214**	**2.4533**	**61.33**	**11.48**	
Instant noodles	0.18	0.101	0.2895	7.24		1985	0.288	0.8275	20.69		694
Beef in soy sauce/curry	0.54	0.003	0.0300	0.75		57471	0.010	0.0850	2.12		20284
Chicken *soto*	0.11	0.405	0.7109	17.77		494	0.900	1.5802	39.51		0
Satay sauce	0.11	0.004	0.0070	0.18		50000	0.016	0.0281	0.70		12500
**TOTAL (by category)**			**7.75**	**193.79**				**21.36**	**534.07**		

OTHERS (minorities) (3-MCPDE)

FOOD ITEM	Representative Occurrence level (mg/kg)	Intake Mean Consumer (kg/day)	Dietary exposure (µg/kg BW/day) Mean consumer	% PMTDI	% of total DE	Mean consumer MOE ($BMDL_{10}$/EDI)	Intake High Consumer P95 (kg/day)	Dietary exposure (µg/kg BW/day) High consumer P95	% PMTDI	% of total DE	High consumer MOE ($BMDL_{10}$/EDI)
**VEGETABLE FATS AND OILS**	**0.83**	**0.016**	**0.2120**	**5.30**	**3.41**		**0.076**	**1.0069**	**25.17**	**5.33**	
Margarine, Shortening	3.02	0.000	0.0000	0.00		0	0.000	0.0000	0.00		0
Mayonnaise	0.14	0.000	0.0000	0.00		0	0.000	0.0000	0.00		0
Evaporated creamer	0.19	0.000	0.0000	0.00		0	0.000	0.0000	0.00		0
Concentrated Creamer	0.76	0.016	0.1931	4.83		12500	0.076	0.9173	22.93		2632
**MILK AND DAIRY**	**0.30**	**0.000**	**0.0000**	**0.00**	**0.00**		**0.000**	**0.0000**	**0.00**	**0.00**	
Evaporated Milk	0.21	0.000	0.0000	0.00		0	0.000	0.0000	0.00		0
Butter	0.38	0.000	0.0000	0.00		0	0.000	0.0000	0.00		0
**CONFECTIONARY**	**0.28**	**0.011**	**0.0492**	**1.23**	**0.79**		**0.057**	**0.2525**	**6.31**	**1.34**	
Flavoured biscuit	0.53	0.003	0.0253	0.63		66667	0.021	0.1731	4.33		9756
Plain biscuit	0.45	0.008	0.0569	1.42		25000	0.036	0.2561	6.40		5556
Bun	0.11	0.000	0.0000	0.00		0	0.000	0.0000	0.00		0
Cake	0.21	0.000	0.0000	0.00		0	0.000	0.0000	0.00		0
Cheese tart, doughnut	0.28	0.000	0.0000	0.00		0	0.000	0.0000	0.00		0
Chocolate bar	0.07	0.000	0.0000	0.00		0	0.000	0.0000	0.00		0
Chocolate spread	0.52	0.000	0.0000	0.00		0	0.000	0.0000	0.00		
**SNACKS**	**0.46**	**0.000**	**0.0000**	**0.00**	**0.00**		**0.000**	**0.0000**	**0.00**	**0.00**	0
Fried fish sausage (Keropok lekor)	0.11	0.000	0.0000	0.00		0	0.000	0.0000	0.00		0
Fried Fish Crackers (Keropok Ikan)	0.59	0.000	0.0000	0.00		0	0.000	0.0000	0.00		0
Murukku (Indian savoury crackers)<br>Potato chips<br>Chicken-flavoured snack<br>Seafood-flavoured snack<br>Fruit/vegetable-flavoured snack	0.49	0.000	0.0000	0.00		0	0.000	0.0000	0.00		0
**LOCAL KUIH-MUIH**	**0.64**	**0.016**	**0.1634**	**4.09**	**2.63**		**0.090**	**0.9194**	**22.98**	**4.87**	
Kuih denderam<br>Prawn fritter<br>Curry puff<br>Cakoi<br>Vadai<br>Banana fritters<br>Fried spring rolls<br>Fried cempedak<br>Fried sweet potato<br>Fried banana balls	0.64	0.016	0.1634	4.09		12500	0.090	0.9194	22.98		2222
**COOKED FOOD (FRIED)**	**0.56**	**0.516**	**4.6123**	**115.31**	**74.22**		**1.518**	**13.5645**	**339.11**	**71.79**	
Fried rice	0.72	0.332	3.8155	95.39		602	0.720	8.2745	206.86		278
Char-kuey-teow, fried rice noodle	0.50	0.048	0.3831	9.58		4167	0.188	1.4998	37.50		1064
Fried wheat noodle	0.23	0.065	0.2386	5.97		3077	0.284	1.0419	26.05		705
Fried Indian Mackerel	0.24	0.046	0.1762	4.41		4348	0.192	0.7355	18.39		1042
Indian flatbread (Roti canai), beef *martabak*	0.45	0.009	0.0646	1.62		22222	0.053	0.3804	9.51		3776
Fried anchovies	1.67	0.003	0.0800	2.00		66667	0.013	0.3364	8.41		15848
Butter prawn	1.05	0.001	0.0168	0.42		200000	0.006	0.0984	2.46		34072
Fried beef<br>Bergedil daging<br>Beef biryani<br>Beef satay	0.49	0.004	0.0313	0.78		50000	0.024	0.1877	4.69		8333
Fried chicken	0.11	0.005	0.0088	0.22		40000	0.024	0.0427	1.07		8220
Fried macaroni	0.11	0.000	0.0000	0.00		0	0.000	0.0000	0.00		0
Fish ball	0.24	0.003	0.0115	0.29		66667	0.014	0.0537	1.34		14265
**FAST FOOD**	**0.23**	**0.003**	**0.0110**	**0.28**	**0.18**		**0.029**	**0.1066**	**2.67**	**0.56**	
Beef burger patty	0.30	0.000	0.0000	0.00		0	0.000	0.0000	0.00		0
Potato fries	0.16	0.000	0.0000	0.00		0	0.000	0.0000	0.00		0
Frankfurter/sausage/hotdog	0.17	0.003	0.0081	0.20		66667	0.029	0.0788	1.97		6887
Nugget	0.20	0.000	0.0000	0.00		0	0.000	0.0000	0.00		0
Pizza with beef and onion	0.11	0.000	0.0000	0.00		0	0.000	0.0000	0.00		0
**COOKED FOOD (STEWED/BOILED)**	**0.18**	**0.406**	**1.1665**	**29.16**	**18.77**		**1.060**	**3.0459**	**76.15**	**16.12**	
Instant noodles	0.18	0.065	0.1868	4.67		3077	0.284	0.8154	20.38		705
Beef in soy sauce/curry	0.54	0.004	0.0345	0.86		50000	0.024	0.2099	5.25		8214
Chicken *soto*	0.11	0.332	0.5829	14.57		602	0.720	1.2642	31.60		278
Satay sauce	0.11	0.005	0.0088	0.22		40000	0.032	0.0562	1.40		6250
**TOTAL (by category)**			**6.21**	**155.36**				**18.90**	**472.40**		

TOTAL POPULATION (GE)

FOOD CATEGORY	Representative Occurrence level (mg/kg)	Intake Mean Consumer (kg/day)	Dietary exposure (µg/kg BW/day) Mean consumer	% of total DE	Mean consumer MOE ($BMDL_{10}$/EDI)	Intake High Consumer P95 (kg/day)	Dietary exposure (µg/kg BW/day) High consumer P95	% of total DE	High consumer MOE ($BMDL_{10}$/EDI)
**VEGETABLE FATS AND OILS**	**0.46**	**0.017**	**0.1275**	**2.31**		**0.092**	**0.6746**	**3.60**	
Margarine, Shortening	4.24	0.001	0.0697		34470	0.006	0.3756		6397
Mayonnaise	0.13	0.000	0.0010		7831250	0.002	0.0041		1888945
Evaporated creamer	0.14	0.001	0.0029		817809	0.009	0.0193		124491
Concentrated Creamer	0.23	0.015	0.0545		44041	0.076	0.2839		8455
**MILK AND DAIRY**	**0.30**	**0.003**	**0.0133**	**0.24**		**0.017**	**0.0791**	**0.42**	
Evaporated Milk	0.15	0.001	0.0031		771077	0.009	0.0204		117377
Butter	0.42	0.001	0.0099		241936	0.008	0.0534		44927
**CONFECTIONARY**	**0.66**	**0.031**	**0.3214**	**5.82**		**0.131**	**1.3757**	**7.34**	
Flavoured biscuit	1.05	0.003	0.0577		41581	0.017	0.2874		8350
Plain biscuit	1.31	0.010	0.2162		11099	0.045	0.9374		2560
Bun	0.14	0.012	0.0259		92757	0.044	0.0977		24571
Cake	0.35	0.002	0.0108		221443	0.010	0.0563		42661
Cheese tart, doughnut	0.46	0.000	0.0030		790512	0.002	0.0120		197628
Chocolate bar	0.09	0.002	0.0035		690358	0.011	0.0164		146678
Chocolate spread	0.42	0.000	0.0016		1495673	0.001	0.0086		280439
**SNACKS**	**0.87**	**0.037**	**0.5127**	**9.28**		**0.171**	**2.3677**	**12.64**	
Fried fish sausage (Keropok lekor)	0.04	0.003	0.0018		1347312	0.011	0.0073		330026
Fried Fish Crackers (Keropok Ikan)	0.61	0.002	0.0184		130419	0.009	0.0887		27057
Murukku (Indian savoury crackers)<br>Potato chips<br>Chicken-flavoured snack<br>Seafood-flavoured snack<br>Fruit/vegetable-flavoured snack	0.95	0.032	0.4889		4886	0.150	2.2745		1050
**LOCAL KUIH-MUIH**	**0.72**	**0.023**	**0.2588**	**4.69**		**0.090**	**1.0343**	**5.52**	
Kuih denderam<br>Prawn fritter<br>Curry puff<br>Cakoi<br>Vadai<br>Banana fritters<br>Fried spring rolls<br>Fried cempedak<br>Fried sweet potato<br>Fried banana balls	0.72	0.023	0.2588		9312	0.090	1.0343		2330
**COOKED FOOD (FRIED)**	**0.32**	**0.489**	**2.4964**	**45.20**		**1.563**	**7.9851**	**42.62**	
Fried rice	0.31	0.275	1.3609		1764	0.720	3.5626		674
Char-kuey-teow, fried rice noodle	0.31	0.065	0.3233		7423	0.211	1.0461		2294
Fried wheat noodle	0.31	0.065	0.3192		7519	0.246	1.2173		1972
Fried Indian Mackerel	0.31	0.047	0.2317		10360	0.128	0.6334		3789
Indian flatbread (Roti canai), beef *martabak*	0.20	0.014	0.0451		53244	0.053	0.1691		14196
Fried anchovies	1.22	0.004	0.0736		32605	0.018	0.3505		6847
Butter prawn	1.30	0.002	0.0415		57831	0.009	0.1772		13544
Fried beef<br>Bergedil daging<br>Beef biryani<br>Beef satay	0.48	0.005	0.0409		58968	0.130	0.9960		18436
Fried chicken	0.31	0.005	0.0226		106134	0.017	0.1690		28871
Fried macaroni	0.31	0.004	0.0211		113591	0.017	0.1063		27924
Fish ball	0.31	0.003	0.0146		163862	0.014	0.0338		34061
**FAST FOOD**	**0.28**	**0.015**	**0.0657**	**1.19**		**0.061**	**0.2741**	**1.46**	
Beef burger patty	0.34	0.007	0.0397		60415	0.022	0.1205		19912
Potato fries	0.31	0.002	0.0117		204655	0.011	0.0549		43697
Frankfurter/sausage/hotdog	0.18	0.002	0.0055		449843	0.015	0.0417		59174
Nugget	0.31	0.001	0.0067		356641	0.006	0.0292		82070
Pizza with beef and onion	0.04	0.002	0.0011		2160345	0.008	0.0048		495257
**COOKED FOOD (STEWED/BOILED)**	**0.31**	**0.349**	**1.7273**	**31.27**		**0.999**	**4.9436**	**26.39**	
Instant noodles	0.39	0.065	0.4016		5976	0.246	1.5314		1567
Beef in soy sauce/curry	0.40	0.005	0.0341		70393	0.017	0.1091		22008
Chicken *soto*	0.04	0.275	0.1756		13668	0.720	0.4597		5221
Satay sauce	0.31	0.004	0.0208		115484	0.016	0.0792		30315
**TOTAL (by category)**		**0.96**	**5.52**			**3.12**	**18.73**		

MALE ADULTS (GE)

FOOD CATEGORY	Representative Occurrence level (mg/kg)	Intake Mean Consumer (kg/day)	Dietary exposure (µg/kg BW/day) Mean consumer	% of total DE	Mean consumer MOE ($BMDL_{10}$/EDI)	Intake High Consumer P95 (kg/day)	Dietary exposure (µg/kg BW/day) High consumer P95	% of total DE	High consumer MOE ($BMDL_{10}$/EDI)
**VEGETABLE FATS AND OILS**	**0.43**	**0.021**	**0.1358**	**2.74**		**0.095**	**0.6163**	**3.96**	
Margarine, Shortening	4.24	0.001	0.0694		34565	0.007	0.4714		5097
Mayonnaise	0.13	0.001	0.0011		2234182	0.002	0.0039		617487
Evaporated creamer	0.14	0.002	0.0033		719429	0.010	0.0212		112950
Concentrated Creamer	0.23	0.018	0.0626		38331	0.076	0.2672		8982
**MILK AND DAIRY**	**0.02**	**0.003**	**0.0010**	**0.02**		**0.018**	**0.0054**	**0.03**	
Evaporated Milk	0.15	0.002	0.0035		678318	0.010	0.0225		106496
Butter	0.42	0.002	0.0103		231959	0.008	0.0503		47731
**CONFECTIONARY**	**0.63**	**0.033**	**0.3085**	**6.22**		**0.127**	**1.2074**	**7.75**	
Flavoured biscuit	1.05	0.004	0.0611		39255	0.017	0.2705		8871
Plain biscuit	1.31	0.009	0.1800		13333	0.038	0.7537		3184
Bun	0.14	0.015	0.0316		75839	0.053	0.1103		21752
Cake	0.35	0.002	0.0102		235264	0.007	0.0394		54665
Cheese tart, doughnut	0.46	0.000	0.0000		0	0.000	0.0000		0
Chocolate bar	0.09	0.002	0.0032		748917	0.011	0.0154		99166
Chocolate spread	0.42	0.000	0.0000		0	0.000	0.0000		0
**SNACKS**	**0.85**	**0.035**	**0.4420**	**8.92**		**0.166**	**2.1261**	**13.65**	
Fried fish sausage (Keropok lekor)	0.04	0.003	0.0020		1181538	0.014	0.0086		280449
Fried Fish Crackers (Keropok Ikan)	0.61	0.002	0.0183		130938	0.010	0.0939		19980
Murukku (Indian savoury crackers)<br>Potato chips<br>Chicken-flavoured snack<br>Seafood-flavoured snack<br>Fruit/vegetable-flavoured snack	0.95	0.029	0.4172		5753	0.142	2.0267		596
**LOCAL KUIH-MUIH**	**0.72**	**0.024**	**0.2616**	**5.28**		**0.090**	**1.0343**	**6.64**	
Kuih denderam<br>Prawn fritter<br>Curry puff<br>Cakoi<br>Vadai<br>Banana fritters<br>Fried spring rolls<br>Fried cempedak<br>Fried sweet potato<br>Fried banana balls	0.72	0.024	0.2616		9176	0.0900	0.9736		3859
**COOKED FOOD (FRIED)**	**0.35**	**0.566**	**2.9751**	**60.02**		**1.592**	**8.3731**	**53.74**	
Fried rice	0.31	0.325	1.5125		1587	0.720	3.3534		640
Char-kuey-teow, fried rice noodle	0.31	0.069	0.3210		7477	0.282	1.3129		2576
Fried wheat noodle	0.31	0.079	0.3661		6555	0.288	1.3413		2521
Fried Indian Mackerel	0.31	0.048	0.2215		10837	0.137	0.6365		5313
Indian flatbread (Roti canai), beef *martabak*	0.20	0.020	0.0607		39560	0.066	0.1989		5791
Fried anchovies	1.22	0.004	0.0708		33922	0.018	0.3288		6315
Butter prawn	1.30	0.002	0.0400		59941	0.009	0.1668		14500
Fried beef<br>Bergedil daging<br>Beef biryani<br>Beef satay	0.48	0.007	0.0510		47072	0.026	0.1846		13168
Fried chicken	0.31	0.006	0.0267		89931	0.017	0.0782		30673
Fried macaroni	0.31	0.004	0.0181		132810	0.016	0.0747		45297
Fish ball	0.31	0.003	0.0148		162556	0.014	0.0663		50991
**FAST FOOD**	**0.32**	**0.018**	**0.0851**	**1.72**		**0.066**	**0.3169**	**2.03**	
Beef burger patty	0.34	0.010	0.0488		49197	0.026	0.1309		15202
Potato fries	0.31	0.003	0.0121		198194	0.011	0.0517		46424
Frankfurter/sausage/hotdog	0.18	0.002	0.0057		424625	0.015	0.0392		102269
Nugget	0.31	0.002	0.0070		341260	0.007	0.0331		102269
Pizza with beef and onion	0.04	0.002	0.0012		2037551	0.008	0.0046		526166
**COOKED FOOD (STEWED/BOILED)**	**0.12**	**0.415**	**0.7475**	**15.08**		**1.054**	**1.9004**	**12.20**	
Instant noodles	0.39	0.079	0.4606		5211	0.288	1.6875		845
Beef in soy sauce/curry	0.40	0.007	0.0425		56487	0.026	0.1538		20105
Chicken *soto*	0.04	0.325	0.1952		12298	0.720	0.4327		1707
Satay sauce	0.31	0.004	0.0197		122110	0.021	0.0955		25137
**TOTAL (by category)**		**1.11**	**4.96**			**3.21**	**15.58**		

FEMALE ADULTS (GE)

FOOD CATEGORY	Representative Occurrence level (mg/kg)	Intake Mean Consumer (kg/day)	Dietary exposure (µg/kg BW/day) Mean consumer	% of total DE	Mean consumer MOE ($BMDL_{10}$/EDI)	Intake High Consumer P95 (kg/day)	Dietary exposure (µg/kg BW/day) High consumer P95	% of total DE	High consumer MOE ($BMDL_{10}$/EDI)
**VEGETABLE FATS AND OILS**	**0.51**	**0.013**	**0.1165**	**2.69**		**0.057**	**0.4903**	**3.27**	
Margarine, Shortening	4.24	0.001	0.0697		34458	0.006	0.4027		5960
Mayonnaise	0.13	0.000	0.0009		2697231	0.002	0.0044		542157
Evaporated creamer	0.14	0.001	0.0024		991709	0.006	0.0138		174290
Concentrated Creamer	0.23	0.011	0.0443		54145	0.043	0.1733		13849
**MILK AND DAIRY**	**0.02**	**0.002**	**0.0007**	**0.02**		**0.013**	**0.0037**	**0.02**	
Evaporated Milk	0.15	0.001	0.0026		935040	0.006	0.0146		164330
Butter	0.42	0.001	0.0093		258918	0.007	0.0503		47715
**CONFECTIONARY**	**0.83**	**0.028**	**0.3918**	**9.05**		**0.125**	**1.7785**	**11.87**	
Flavoured biscuit	1.05	0.003	0.0536		44794	0.021	0.3698		6490
Plain biscuit	1.31	0.012	0.2613		9185	0.045	1.0050		2388
Bun	0.14	0.008	0.0186		128849	0.035	0.0838		28645
Cake	0.35	0.002	0.0116		206563	0.011	0.0648		37036
Cheese tart, doughnut	0.46	0.000	0.0028		871155	0.002	0.0129		185917
Chocolate bar	0.09	0.002	0.0038		625863	0.011	0.0175		136822
Chocolate spread	0.42	0.000	0.0016		1517922	0.000	0.0019		1284396
**SNACKS**	**0.89**	**0.039**	**0.6003**	**13.87**		**0.204**	**3.0969**	**20.67**	
Fried fish sausage (Keropok lekor)	0.04	0.002	0.0015		1638505	0.009	0.0058		410585
Fried Fish Crackers (Keropok Ikan)	0.61	0.002	0.0185		101591	0.009	0.0891		26924
Murukku (Indian savoury crackers)<br>Potato chips<br>Chicken-flavoured snack<br>Seafood-flavoured snack<br>Fruit/vegetable-flavoured snack	0.95	0.036	0.5779		4153	0.187	3.0317		792
**LOCAL KUIH-MUIH**	**0.72**	**0.021**	**0.2370**	**5.48**		**0.090**	**1.0300**	**6.87**	
Kuih denderam<br>Prawn fritter<br>Curry puff<br>Cakoi<br>Vadai<br>Banana fritters<br>Fried spring rolls<br>Fried cempedak<br>Fried sweet potato<br>Fried banana balls	0.72	0.021	0.2552		792	0.090	1.1088		2164
**COOKED FOOD (FRIED)**	**0.35**	**0.405**	**2.3954**	**55.34**		**1.159**	**6.8646**	**45.81**	
Fried rice	0.31	0.221	1.1702		2051	0.540	2.8645		838
Char-kuey-teow, fried rice noodle	0.31	0.061	0.3261		7360	0.188	0.9969		2407
Fried wheat noodle	0.31	0.049	0.2611		9192	0.185	0.9787		2452
Fried Indian Mackerel	0.31	0.046	0.2441		9831	0.128	0.6790		3535
Indian flatbread (Roti canai), beef *martabak*	0.20	0.007	0.0255		94005	0.026	0.0906		26483
Fried anchovies	1.22	0.004	0.0770		31156	0.018	0.3758		6387
Butter prawn	1.30	0.002	0.0432		55613	0.008	0.1791		13402
Fried beef<br>Bergedil daging<br>Beef biryani<br>Beef satay	0.48	0.003	0.0283		84696	0.013	0.1052		22810
Fried chicken	0.31	0.003	0.0176		136688	0.017	0.0891		26931
Fried macaroni	0.31	0.005	0.0249		96264	0.024	0.1276		18812
Fish ball	0.31	0.003	0.0145		165728	0.013	0.0680		35319
**FAST FOOD**	**0.31**	**0.011**	**0.0602**	**1.39**		**0.055**	**0.2901**	**1.94**	
Beef burger patty	0.34	0.005	0.0286		83845	0.022	0.1292		18574
Potato fries	0.31	0.002	0.0112		213414	0.010	0.0544		44140
Frankfurter/sausage/hotdog	0.18	0.002	0.0052		458353	0.010	0.0298		80496
Nugget	0.31	0.001	0.0064		377032	0.005	0.0282		85045
Pizza with beef and onion	0.04	0.001	0.0010		2353289	0.008	0.0052		461976
**COOKED FOOD (STEWED/BOILED)**	**0.11**	**0.277**	**0.5269**	**12.17**		**0.753**	**1.4308**	**9.55**	
Instant noodles	0.39	0.049	0.3285		7307	0.185	1.2313		1949
Beef in soy sauce/curry	0.40	0.003	0.0236		101635	0.013	0.0877		27372
Chicken *soto*	0.04	0.221	0.1510		15895	0.540	0.3696		6493
Satay sauce	0.31	0.004	0.0221		108759	0.016	0.0849		28277
**TOTAL (by category)**		**0.80**	**4.33**			**2.46**	**14.98**		

MALAY (GE)

FOOD CATEGORY	Representative Occurrence level (mg/kg)	Intake Mean Consumer (kg/day)	Dietary exposure (µg/kg BW/day) Mean consumer	% of total DE	Mean consumer MOE ($BMDL_{10}$/EDI)	Intake High Consumer P95 (kg/day)	Dietary exposure (µg/kg BW/day) High consumer P95	% of total DE	High consumer MOE ($BMDL_{10}$/EDI)
**VEGETABLE FATS AND OILS**	**0.44**	**0.0219**	**0.1528**	**3.48**		**0.0960**	**0.6696**	**3.37**	
Margarine, Shortening	4.24	0.0012	0.0812		30088	0.0070	0.4737		5066
Mayonnaise	0.13	0.0006	0.0012		6062903	0.0030	0.0062		385538
Evaporated creamer	0.14	0.0016	0.0036		664470	0.0100	0.0226		106315
Concentrated Creamer	0.23	0.0185	0.0691		34752	0.0760	0.2839		8455
**MILK AND DAIRY**	**0.26**	**0.0028**	**0.0118**	**0.27**		**0.0160**	**0.0672**	**0.73**	
Evaporated Milk	0.15	0.0016	0.0038		626500	0.0100	0.0239		100240
Butter	0.42	0.0012	0.0080		308677	0.0060	0.0402		59678
**CONFECTIONARY**	**0.68**	**0.0316**	**0.3415**	**7.77**		**0.1380**	**1.4912**	**0.10**	
Flavoured biscuit	1.05	0.0040	0.0673		35567	0.0210	0.3534		6792
Plain biscuit	1.31	0.0111	0.2312		10342	0.0450	0.9374		2560
Bun	0.14	0.0117	0.0259		92916	0.0530	0.1173		20459
Cake	0.35	0.0015	0.0084		292245	0.0050	0.0279		85920
Cheese tart, doughnut	0.46	0.0003	0.0022		1117620	0.0020	0.0147		163435
Chocolate bar	0.09	0.0027	0.0039		609732	0.0110	0.0158		151879
Chocolate spread	0.42	0.0003	0.0020		1237798	0.0010	0.0067		358000
**SNACKS**	**0.85**	**0.0484**	**0.6567**	**14.94**		**0.2460**	**3.3376**	**0.04**	
Fried fish sausage (Keropok lekor)	0.04	0.0045	0.0029		840940	0.0200	0.0128		187950
Fried Fish Crackers (Keropok Ikan)	0.61	0.0028	0.0273		87720	0.0120	0.1168		20541
Murukku (Indian savoury crackers)<br>Potato chips<br>Chicken-flavoured snack<br>Seafood-flavoured snack<br>Fruit/vegetable-flavoured snack	0.95	0.0411	0.6232		3832	0.2140	3.2450		740
**LOCAL KUIH-MUIH**	**0.72**	**0.0282**	**0.3241**	**7.37**		**0.0900**	**1.0343**	**11.46**	
Kuih denderam<br>Prawn fritter<br>Curry puff<br>Cakoi<br>Vadai<br>Banana fritters<br>Fried spring rolls<br>Fried cempedak<br>Fried sweet potato<br>Fried banana balls	0.72	0.0282	0.0000		7426	0.0900	1.0343		2320
**COOKED FOOD (FRIED)**	**0.32**	**0.4619**	**2.3593**	**53.68**		**1.1130**	**5.6849**	**1.96**	
Fried rice	0.31	0.2636	1.3043		1840	0.4800	2.3751		1010
Char-kuey-teow, fried rice noodle	0.31	0.0546	0.2702		8892	0.1880	0.9302		2580
Fried wheat noodle	0.31	0.0449	0.2222		10795	0.1640	0.8115		2958
Fried Indian Mackerel	0.31	0.0538	0.2662		9020	0.1280	0.6334		3789
Indian flatbread (Roti canai), beef *martabak*	0.20	0.0193	0.0616		39034	0.0530	0.1692		14185
Fried anchovies	1.22	0.0041	0.0798		29842	0.0180	0.3505		6847
Butter prawn	1.30	0.0022	0.0457		53300	0.0090	0.1868		12851
Fried beef<br>Bergedil daging<br>Beef biryani<br>Beef satay	0.48	0.0079	0.0605		39759	0.0260	0.1992		12048
Fried chicken	0.31	0.0052	0.0257		93096	0.0170	0.1690		14199
Fried macaroni	0.31	0.0041	0.0203		118301	0.0170	0.1063		22573
Fish ball	0.31	0.0022	0.0109		217503	0.0130	0.0338		70978
**FAST FOOD**	**0.22**	**0.0073**	**0.0258**	**0.59**		**0.0360**	**0.1270**		
Beef burger patty	0.34	0.0000	0.0000		0	0.0000	0.0000		0
Potato fries	0.31	0.0025	0.0124		197972	0.0110	0.0544		44094
Frankfurter/sausage/hotdog	0.18	0.0014	0.0040		605070	0.0100	0.0287		83533
Nugget	0.31	0.0017	0.0084		292188	0.0070	0.0346		69290
Pizza with beef and onion	0.04	0.0017	0.0011		2278182	0.0080	0.0051		469875
**COOKED FOOD (STEWED/BOILED)**	**0.10**	**0.3214**	**0.5233**	**11.91**		**0.4780**	**0.7782**	**0.14**	
Instant noodles	0.39	0.0449	0.2795		8581	0.1640	1.0209		2351
Beef in soy sauce/curry	0.40	0.0079	0.0504		47462	0.0260	0.1660		14458
Chicken *soto*	0.04	0.2633	0.1681		14276	0.2640	0.1686		14239
Satay sauce	0.31	0.0053	0.0262		91516	0.0240	0.1188		20210
**TOTAL (by category)**			**4.40**				**13.19**		

CHINESE (GE)

FOOD CATEGORY	Representative Occurrence level (mg/kg)	Intake Mean Consumer (kg/day)	Dietary exposure (µg/kg BW/day) Mean consumer	% of total DE	Mean consumer MOE ($BMDL_{10}$/EDI)	Intake High Consumer P95 (kg/day)	Dietary exposure (µg/kg BW/day) High consumer P95	% of total DE	High consumer MOE ($BMDL_{10}$/EDI)
**VEGETABLE FATS AND OILS**	**4.24**	**0.001**	**0.0677**	**1.81**		**0.006**	**0.3756**	**3.24**	
Margarine, Shortening	4.24	0.001	0.0677		35504	0.006	0.3756		6390
Mayonnaise	0.13	0.000	0.0000		0	0.000	0.0000		0
Evaporated creamer	0.14	0.000	0.0000		0	0.000	0.0000		0
Concentrated Creamer	0.23	0.000	0.0000		0	0.000	0.0000		0
**MILK AND DAIRY**	**0.42**	**0.002**	**0.0139**	**0.37**		**0.012**	**0.0804**	**0.69**	
Evaporated Milk	0.15	0.000	0.0000		0	0.000	0.0000		0
Butter	0.42	0.002	0.0139		172147	0.012	0.0804		29839
**CONFECTIONARY**	**0.57**	**0.021**	**0.1930**	**5.16**		**0.105**	**0.9551**	**8.23**	
Flavoured biscuit	1.05	0.002	0.0288		83405	0.010	0.6058		3962
Plain biscuit	1.31	0.006	0.1260		19043	0.036	0.7356		3263
Bun	0.14	0.009	0.0189		126822	0.035	0.0239		100215
Cake	0.35	0.002	0.0130		184378	0.011	0.0092		261951
Cheese tart, doughnut	0.46	0.000	0.0030		790512	0.002	0.0836		28698
Chocolate bar	0.09	0.002	0.0032		759394	0.011	0.0000		0
Chocolate spread	0.42	0.000	0.0000		0	0.000	0.5229		4590
**SNACKS**	**0.92**	**0.013**	**0.1846**	**4.94**		**0.078**	**1.1458**	**9.87**	
Fried fish sausage (Keropok lekor)	0.04	0.000	0.0003		7997872	0.002	0.0013		1879500
Fried Fish Crackers (Keropok Ikan)	0.61	0.000	0.0000		0	0.000	0.0000		0
Murukku (Indian savoury crackers)<br>Potato chips<br>Chicken-flavoured snack<br>Seafood-flavoured snack<br>Fruit/vegetable-flavoured snack	0.95	0.012	0.1835		13051	0.076	1.1524		2083
**LOCAL KUIH-MUIH**	**0.72**	**0.007**	**0.0770**	**2.06**		**0.034**	**0.3907**	**3.37**	
Kuih denderam<br>Prawn fritter<br>Curry puff<br>Cakoi<br>Vadai<br>Banana fritters<br>Fried spring rolls<br>Fried cempedak<br>Fried sweet potato<br>Fried banana balls	0.72	0.007	0.0770		31300	0.034	0.3907		6142
**COOKED FOOD (FRIED)**	**0.32**	**0.472**	**2.4120**	**64.47**		**1.293**	**6.6043**	**56.91**	
Fried rice	0.31	0.220	1.0892		2203	0.489	2.4196		992
Char-kuey-teow, fried rice noodle	0.31	0.102	0.5059		4744	0.330	1.6329		1470
Fried wheat noodle	0.31	0.096	0.4739		5064	0.288	1.4251		1684
Fried Indian Mackerel	0.31	0.026	0.1298		18484	0.064	0.3167		7579
Indian flatbread (Roti canai), beef *martabak*	0.20	0.007	0.0235		102286	0.026	0.0830		28915
Fried anchovies	1.22	0.002	0.0321		74694	0.009	0.1753		13694
Butter prawn	1.30	0.002	0.0438		54816	0.009	0.1868		12851
Fried beef<br>Bergedil daging<br>Beef biryani<br>Beef satay	0.48	0.002	0.0138		171136	0.009	0.0690		34806
Fried chicken	0.31	0.004	0.0175		137015	0.017	0.1690		14199
Fried macaroni	0.31	0.006	0.0315		76263	0.035	0.1063		22573
Fish ball	0.31	0.005	0.0249		96428	0.017	0.0338		70978
**FAST FOOD**	**0.23**	**0.011**	**0.0386**	**1.03**		**0.048**	**0.1771**	**1.53**	
Beef burger patty	0.34	0.006	0.0313		76644	0.022	0.1205		19912
Potato fries	0.31	0.002	0.0100		238932	0.011	0.0549		43697
Frankfurter/sausage/hotdog	0.18	0.001	0.0023		1087595	0.004	0.0128		186875
Nugget	0.31	0.000	0.0023		1031984	0.002	0.0121		197972
Pizza with beef and onion	0.04	0.001	0.0009		2592414	0.008	0.0051		469875
**COOKED FOOD (STEWED/BOILED)**	**0.15**	**0.319**	**0.7547**	**20.17**		**0.794**	**1.8754**	**16.16**	
Instant noodles	0.39	0.0958	0.5962		4025	0.288	1.7928		1339
Beef in soy sauce/curry	0.40	0.0018	0.0117		204293	0.009	0.0566		42379
Chicken *soto*	0.04	0.2201	0.1405		17077	0.489	0.3122		7687
Satay sauce	0.31	0.0017	0.0085		281995	0.008	0.0396		60629
**TOTAL (by category)**			**3.74**				**11.60**		

INDIAN (GE)

FOOD CATEGORY	Representative Occurrence level (mg/kg)	Intake Mean Consumer (kg/day)	Dietary exposure (µg/kg BW/day) Mean consumer	% of total DE	Mean consumer MOE ($BMDL_{10}$/EDI)	Intake High Consumer P95 (kg/day)	Dietary exposure (µg/kg BW/day) High consumer P95	% of total DE	High consumer MOE ($BMDL_{10}$/EDI)
**VEGETABLE FATS AND OILS**	**4.24**	**0.001**	**0.0947**	**3.06**	**0**	**0.007**	**0.4737**	**4.60**	
Margarine, Shortening	4.24	0.001	0.0947		0	0.007	0.4737		5066
Mayonnaise	0.13	0.000	0.0000		0	0.000	0.0000		0
Evaporated creamer	0.14	0.000	0.0000		0	0.000	0.0000		0
Concentrated Creamer	0.23	0.000	0.0000		0	0.000	0.0000		0
**MILK AND DAIRY**	**0.30**	**0.000**	**0.0000**	**0.00**	**0**	**0.000**	**0.0000**	**0.00**	
Evaporated Milk	0.15	0.000	0.0000		0	0.000	0.0000		0
Butter	0.42	0.000	0.0000		0	0.000	0.0000		0
**CONFECTIONARY**	**1.18**	**0.017**	**0.3170**	**10.23**	**7896**	**0.065**	**1.2229**	**11.87**	
Flavoured biscuit	1.05	0.000	0.0000		0	0.000	0.0000		0
Plain biscuit	1.31	0.015	0.3041		199814	0.054	1.1249		2133
Bun	0.14	0.000	0.0000		0	0.000	0.0000		0
Cake	0.35	0.002	0.0123		0	0.011	0.0604		39704
Cheese tart, doughnut	0.46	0.000	0.0000		0	0.000	0.0000		0
Chocolate bar	0.09	0.000	0.0000		0	0.000	0.0000		0
Chocolate spread	0.42	0.000	0.0000		0	0.000	0.0000		0
**SNACKS**	**0.61**	**0.003**	**0.0292**	**0.94**	**795135**	**0.002**	**0.0222**	**0.22**	
Fried fish sausage (Keropok lekor)	0.04	0.000	0.0000		0	0.000	0.0000		0
Fried Fish Crackers (Keropok Ikan)	0.61	0.003	0.0292		0	0.002	0.0222		108110
Murukku (Indian savoury crackers)<br>Potato chips<br>Chicken-flavoured snack<br>Seafood-flavoured snack<br>Fruit/vegetable-flavoured snack	0.95	0.000	0.0000		0	0.000	0.0000		0
**LOCAL KUIH-MUIH**	**0.72**	**0.021**	**0.2448**	**7.90**		**0.085**	**0.9769**	**9.48**	
Kuih denderam<br>Prawn fritter<br>Curry puff<br>Cakoi<br>Vadai<br>Banana fritters<br>Fried spring rolls<br>Fried cempedak<br>Fried sweet potato<br>Fried banana balls	0.72	0.021	0.2448		9845	0.085	0.9769		2457
**COOKED FOOD (FRIED)**	**0.32**	**0.368**	**1.8791**	**60.62**		**1.193**	**6.0945**	**59.16**	
Fried rice	0.31	0.213	1.0540		2277	0.480	2.3751		1010
Char-kuey-teow, fried rice noodle	0.31	0.073	0.3592		6680	0.251	1.2437		1930
Fried wheat noodle	0.31	0.000	0.0000		8293	0.238	1.1767		2040
Fried Indian Mackerel	0.31	0.059	0.2895		13862	0.128	0.6334		3789
Indian flatbread (Roti canai), beef *martabak*	0.20	0.014	0.0447		53738	0.053	0.1691		14196
Fried anchovies	1.22	0.004	0.0721		33400	0.013	0.2495		9621
Butter prawn	1.30	0.001	0.0249		94034	0.005	0.1064		22546
Fried beef<br>Bergedil daging<br>Beef biryani<br>Beef satay	0.48	0.000	0.0000		0	0.000	0.0000		0
Fried chicken	0.31	0.002	0.0109		218483	0.012	0.1690		14199
Fried macaroni	0.31	0.000	0.0000		0	0.000	0.1063		22573
Fish ball	0.31	0.003	0.0134		177019	0.013	0.0338		70978
**FAST FOOD**	**0.33**	**0.007**	**0.0364**	**1.18**		**0.037**	**0.1937**	**1.88**	
Beef burger patty	0.34	0.004	0.0227		105545	0.022	0.1205		19912
Potato fries	0.31	0.002	0.0079		305052	0.008	0.0381		63073
Frankfurter/sausage/hotdog	0.18	0.000	0.0000		0	0.000	0.0000		0
Nugget	0.31	0.001	0.0058		411044	0.007	0.0351		68314
Pizza with beef and onion	0.04	0.000	0.0000		0	0.000	0.0000		0
**COOKED FOOD (STEWED/BOILED)**	**0.12**	**0.271**	**0.4983**	**16.08**	**0**	**0.718**	**1.3176**	**12.79**	
Instant noodles	0.39	0.058	0.3641		6592	0.238	1.4804		1621
Beef in soy sauce/curry	0.40	0.000	0.0000		0	0.000	0.0000		0
Chicken *soto*	0.04	0.213	0.1360		17648	0.480	0.3065		7831
Satay sauce	0.31	0.000	0.0000		0	0.000	0.0000		0
**TOTAL (by category)**		**0.69**	**3.10**			**2.11**	**10.30**		

OTHERS (bumiputra) (GE)

FOOD CATEGORY	Representative Occurrence level (mg/kg)	Intake Mean Consumer (kg/day)	Dietary exposure (µg/kg BW/day) Mean consumer	% of total DE	Mean consumer MOE ($BMDL_{10}$/EDI)	Intake High Consumer P95 (kg/day)	Dietary exposure (µg/kg BW/day) High consumer P95	% of total DE	High consumer MOE ($BMDL_{10}$/EDI)
**VEGETABLE FATS AND OILS**	**0.22**	**0.010**	**0.0351**	**0.64**		**0.045**	**0.1569**	**0.97**	
Margarine, Shortening	4.24	0.000	0.0000		0	0.000	0.0000		0
Mayonnaise	0.13	0.000	0.0000		25060000	0.000	0.0000		0
Evaporated creamer	0.14	0.001	0.0000		857380	0.007	0.0151		158917
Concentrated Creamer	0.23	0.009	0.0037		74285	0.038	0.1419		16910
**MILK AND DAIRY**	**0.26**	**0.002**	**0.0087**	**0.16**		**0.013**	**0.0527**	**0.33**	
Evaporated Milk	0.15	0.001	0.0030		808387	0.007	0.0160		149836
Butter	0.42	0.001	0.0058		416355	0.006	0.0401		59877
**CONFECTIONARY**	**0.73**	**0.029**	**0.3422**	**6.28**		**0.123**	**1.4234**	**8.81**	
Flavoured biscuit	1.05	0.003	0.0538		44570	0.021	0.3450		6957
Plain biscuit	1.31	0.012	0.2575		9321	0.049	1.0249		2342
Bun	0.14	0.014	0.0308		77841	0.053	0.1172		20474
Cake	0.39	0.000	0.0000		0	0.000	0.0000		0
Cheese tart, doughnut	0.46	0.000	0.0000		0	0.000	0.0000		0
Chocolate bar	0.09	0.000	0.0000		0	0.000	0.0000		0
Chocolate spread	0.42	0.000	0.0000		0	0.000	0.0000		0
**SNACKS**	**0.91**	**0.039**	**0.5755**	**10.56**		**0.181**	**2.6405**	**16.35**	
Fried fish sausage (Keropok lekor)	0.04	0.001	0.0009		2743796	0.006	0.0035		683455
Fried Fish Crackers (Keropok Ikan)	0.61	0.001	0.0109		220082	0.006	0.0554		43320
Murukku (Indian savoury crackers)<br>Potato chips<br>Chicken-flavoured snack<br>Seafood-flavoured snack<br>Fruit/vegetable-flavoured snack	0.95	0.037	0.5611		4239	0.170	2.5778		931
**LOCAL KUIH-MUIH**	**0.72**	**0.026**	**0.2976**	**5.46**		**0.090**	**1.0300**	**6.38**	
Kuih denderam<br>Prawn fritter<br>Curry puff<br>Cakoi<br>Vadai<br>Banana fritters<br>Fried spring rolls<br>Fried cempedak<br>Fried sweet potato<br>Fried banana balls	0.72	0.026	0.2988		8035	0.090	1.0343		2320
**COOKED FOOD (FRIED)**	**0.32**	**0.638**	**3.2071**	**58.85**		**1.666**	**8.3767**	**51.87**	
Fried rice	0.31	0.405	2.0033		1198	0.900	4.4533		539
Char-kuey-teow, fried rice noodle	0.31	0.059	0.2933		8182	0.211	1.0461		2294
Fried wheat noodle	0.31	0.101	0.4985		4814	0.288	1.4251		1684
Fried Indian Mackerel	0.31	0.055	0.2709		8861	0.192	0.9500		2526
Indian flatbread (Roti canai), beef *martabak*	0.20	0.006	0.0185		130748	0.026	0.0845		28391
Fried anchovies	1.22	0.000	0.0000		0	0.000	0.0000		0
Butter prawn	1.30	0.002	0.0367		65346	0.008	0.1596		15041
Fried beef<br>Bergedil daging<br>Beef biryani<br>Beef satay	0.48	0.003	0.0230		90486	0.010	0.0766		31325
Fried chicken	0.31	0.005	0.0230		104533	0.017	0.1690		14199
Fried macaroni	0.31	0.000	0.0000		0	0.000	0.1063		22573
Fish ball	0.31	0.003	0.0148		153007	0.013	0.0338		70978
**FAST FOOD**	**0.26**	**0.014**	**0.0575**	**1.06**		**0.067**	**0.2797**	**1.73**	
Beef burger patty	0.34	0.005	0.0251		95515	0.022	0.1205		19912
Potato fries	0.31	0.002	0.0094		256631	0.008	0.0381		63073
Frankfurter/sausage/hotdog	0.18	0.006	0.0165		149167	0.029	0.0834		28765
Nugget	0.31	0.001	0.0070		341572	0.008	0.0381		63073
Pizza with beef and onion	0.04	0.000	0.0000		0	0.000	0.0000		0
**COOKED FOOD (STEWED/BOILED)**	**0.11**	**0.513**	**0.9255**	**16.98**		**1.2139**	**2.1894**	**13.56**	
Instant noodles	0.39	0.101	0.6272		3827	0.288	1.7928		1339
Beef in soy sauce/curry	0.40	0.003	0.0222		108017	0.010	0.0630		38124
Chicken *soto*	0.04	0.405	0.2585		9284	0.900	0.5746		4177
Satay sauce	0.31	0.004	0.0198		113061	0.016	0.0792		30315
**TOTAL (by category)**		**1.27**	**5.45**			**3.40**	**16.15**		

OTHERS (minorities) (GE)

FOOD CATEGORY	Representative Occurrence level (mg/kg)	Intake Mean Consumer (kg/day)	Dietary exposure (μg/kg BW/day) Mean consumer	% of total DE	Mean consumer MOE ($BMDL_{10}$/EDI)	Intake High Consumer P95 (kg/day)	Dietary exposure (μg/kg BW/day) High consumer P95	% of total DE	High consumer MOE ($BMDL_{10}$/EDI)
**VEGETABLE FATS AND OILS**	**0.23**	**0.016**	**0.0609**	**1.66**		**0.076**	**0.2839**	**2.37**	
Margarine, Shortening	4.24	0.000	0.0000		0	0.000	0.0000		0
Mayonnaise	0.13	0.000	0.0000		0	0.000	0.0000		0
Evaporated creamer	0.14	0.000	0.0000		0	0.000	0.0000		0
Concentrated Creamer	0.23	0.0163	0.0609		39349	0.076	0.2839		8455
**MILK AND DAIRY**	**0.00**	**0.000**	**0.0000**	**0.00**		**0.000**	**0.0000**	**0.00**	
Evaporated Milk	0.15	0.000	0.0000		0	0.000	0.0000		0
Butter	0.42	0.000	0.0000		0	0.000	0.0000		0
**CONFECTIONARY**	**1.24**	**0.011**	**0.2258**	**6.14**		**0.057**	**1.1174**	**9.32**	
Flavoured biscuit	1.05	0.003	0.0507		47383	0.021	0.3450		6957
Plain biscuit	1.31	0.008	0.1752		13699	0.036	0.7499		3200
Bun	0.14	0.000	0.0000		0	0.000	0.0000		0
Cake	0.39	0.000	0.0000		0	0.000	0.0000		0
Cheese tart, doughnut	0.46	0.000	0.0000		0	0.000	0.0000		0
Chocolate bar	0.09	0.000	0.0000		0	0.000	0.0000		0
Chocolate spread	0.42	0.000	0.0000		0	0.000	0.0000		0
**SNACKS**	**0.00**	**0.000**	**0.0000**	**0.00**		**0.000**	**0.0000**	**0.00**	
Fried fish sausage (Keropok lekor)	0.04	0.000	0.0000		0	0.000	0.0000		0
Fried Fish Crackers (Keropok Ikan)	0.61	0.000	0.0000		0	0.000	0.0000		0
Murukku (Indian savoury crackers)<br>Potato chips<br>Chicken-flavoured snack<br>Seafood-flavoured snack<br>Fruit/vegetable-flavoured snack	0.95	0.000	0.0000		0	0.000	0.0000		0
**LOCAL KUIH-MUIH**	**0.72**	**0.016**	**0.1816**	**4.94**		**0.090**	**1.0343**	**8.62**	
Kuih denderam<br>Prawn fritter<br>Curry puff<br>Cakoi<br>Vadai<br>Banana fritters<br>Fried spring rolls<br>Fried cempedak<br>Fried sweet potato<br>Fried banana balls	0.72	0.016	0.1816		13273	0.090	1.0343		2320
**COOKED FOOD (FRIED)**	**0.32**	**0.519**	**2.6510**	**72.11**		**1.518**	**7.7512**	**64.63**	
Fried rice	0.31	0.332	1.6449		1459	0.720	3.5626		674
Char-kuey-teow, fried rice noodle	0.31	0.048	0.2375		10105	0.188	0.9299		2581
Fried wheat noodle	0.31	0.065	0.3206		7486	0.284	1.4043		1709
Fried Indian Mackerel	0.31	0.046	0.2290		10480	0.192	0.9500		2526
Indian flatbread (Roti canai), beef *martabak*	0.20	0.009	0.0301		79809	0.053	0.1691		14196
Fried anchovies	1.22	0.003	0.0586		40945	0.013	0.2458		9766
Butter prawn	1.30	0.001	0.0309		77625	0.006	0.1218		19704
Fried beef<br>Bergedil daging<br>Beef biryani<br>Beef satay	0.48	0.004	0.0291		82217	0.024	0.1839		13052
Fried chicken	0.31	0.005	0.0232		103418	0.024	0.1690		14199
Fried macaroni	0.31	0.000	0.0000		0	0.000	0.1063		22573
Fish ball	0.31	0.003	0.0126		190209	0.014	0.0338		70978
**FAST FOOD**	**0.18**	**0.003**	**0.0071**	**0.19**		**0.029**	**0.0810**	**0.68**	
Beef burger patty	0.34	0.000	0.0000		0	0.000	0.0000		0
Potato fries	0.31	0.000	0.0000		0	0.000	0.0000		0
Frankfurter/sausage/hotdog	0.18	0.003	0.0073		336941	0.029	0.0833		28805
Nugget	0.31	0.000	0.0000		0	0.000	0.0000		0
Pizza with beef and onion	0.04	0.000	0.0000		0	0.000	0.0000		0
**COOKED FOOD (STEWED/BOILED)**	**0.10**	**0.3378**	**0.5500**	**14.96**		**1.0602**	**1.7260**	**14.39**	
Instant noodles	0.39	0.0004	0.0024		5951	0.284	1.7667		1358
Beef in soy sauce/curry	0.40	0.0004	0.0026		98146	0.024	0.1555		15437
Chicken *soto*	0.04	0.332	0.2122		11308	0.720	0.4597		5221
Satay sauce	0.31	0.005	0.0228		104759	0.032	0.1583		15157
**TOTAL (by category)**			**3.68**				**11.99**		
