# Supplementary material for: Hidden Hazards: Assessment of Exposure Risks from 3-Monochloropropane-1,2-diol Ester (3-MCPDE) and Glycidyl Ester (GE) Consumption Among Malaysian Consumers
Source: Toxics. 2026 Apr 16;14(4):331. doi: 10.3390/toxics14040331 (PMC13119714; doi:10.3390/toxics14040331)
Supplement: Supplementary file 1 [file toxics-14-00331-s001.zip › Supplementary S3_LCR,ACR,DALY.pdf]

TOTAL POPULATION (GE)														
**FOOD ITEM**	**Mean consumer LADD (mg/kg/day)**	**Mean consumer LCR (/100,000 cases)**	**Mean consumer ACR (cases/year/100,000)**	**Mean consumer Glycidol fraction related cancer cases**	**DALY (/100,000 cases)**	**DALY UI (2.5%)**	**DALY UI (97.5%)**	**High consumer LADD (p95) (mg/kg/day)**	**High consumer LCR (/100,000 cases) p95**	**High consumer ACR (cases/year/100,000)**	**High consumer Glycidol fraction related cancer cases**	**DALY (/100,000 cases)**	**DALY UI (2.5%)**	**DALY UI (97.5%)**
**VEGETABLE FATS AND OILS**	**1.19E-04**	**15.41**	**0.20**	**1.38E-03**	**3.71**	**3.07**	**4.45**	**6.27E-04**	**81.56**	**1.08**	**7.29E-03**	**19.66**	**16.26**	**23.55**
Margarine, Shortening	6.48E-05	8.43	0.11	7.53E-04	2.03	1.68	2.43	3.49E-04	45.40	0.63	4.21E-03	4.60	9.39	13.60
Mayonnaise	2.85E-07	0.04	0.00	3.31E-06	0.00	0.01	0.01	1.18E-06	0.15	0.00	1.42E-05	0.02	0.03	0.05
Evaporated creamer	2.73E-06	0.36	0.00	3.17E-05	0.03	0.07	0.10	1.79E-05	2.33	0.03	2.16E-04	0.24	0.48	0.70
Concentrated Creamer	5.07E-05	6.59	0.09	5.89E-04	0.64	1.31	1.90	2.64E-04	34.35	0.47	3.18E-03	3.48	7.10	10.29
**MILK AND DAIRY**	**1.21E-05**	**1.58**	**0.02**	**1.41E-04**	**0.38**	**0.31**	**0.46**	**7.20E-05**	**9.37**	**0.12**	**8.37E-04**	**2.26**	**1.87**	**2.70**
Evaporated Milk	2.90E-06	0.38	0.01	3.36E-05	0.04	0.08	0.11	1.90E-05	2.47	0.03	2.29E-04	0.25	0.51	0.74
Butter	9.23E-06	1.20	0.02	1.07E-04	0.12	0.24	0.35	4.97E-05	6.46	0.09	5.99E-04	0.66	1.34	1.94
**CONFECTIONARY**	**2.97E-04**	**38.58**	**0.51**	**3.45E-03**	**9.30**	**7.69**	**11.14**	**1.27E-03**	**165.11**	**2.20**	**1.48E-02**	**39.80**	**32.92**	**47.68**
Flavoured biscuit	5.37E-05	6.98	0.09	6.24E-04	0.68	1.39	2.02	2.68E-04	34.78	0.48	3.22E-03	3.53	7.19	10.42
Plain biscuit	2.01E-04	26.17	0.35	2.34E-03	2.56	5.22	7.56	8.73E-04	113.44	1.56	1.05E-02	11.50	23.46	33.98
Bun	6.95E-06	0.90	0.01	8.07E-05	0.09	0.18	0.26	9.09E-05	11.82	0.16	1.10E-03	1.20	2.44	3.54
Cake	1.01E-05	1.31	0.02	1.17E-04	0.13	0.26	0.38	5.24E-05	6.81	0.09	6.31E-04	0.69	1.41	2.04
Cheese tart, doughnut	2.83E-06	0.37	0.00	3.28E-05	0.1	0.1	0.1	1.13E-05	1.47	0.02	1.36E-04	0.15	0.30	0.44
Chocolate bar	3.24E-06	0.42	0.01	3.76E-05	0.04	0.08	0.12	1.52E-05	1.98	0.03	1.83E-04	0.20	0.41	0.59
Chocolate spread	1.49E-06	0.19	0.00	1.73E-05	0.02	0.04	0.06	7.97E-06	1.04	0.01	9.60E-05	0.10	0.21	0.31
**SNACKS**	**4.76E-04**	**61.88**	**0.82**	**5.53E-03**	**14.92**	**12.34**	**17.87**	**2.20E-03**	**285.78**	**3.80**	**2.55E-02**	**68.88**	**56.97**	**82.52**
Fried fish sausage (*Keropok lekor*)	1.66E-06	0.22	0.00	1.93E-05	0.02	0.04	0.06	6.77E-06	0.88	0.01	8.15E-05	0.09	0.18	0.26
Fried Fish Crackers (*Keropok Ikan*)	1.71E-05	2.23	0.03	1.99E-04	0.22	0.44	0.64	8.26E-05	10.73	0.15	9.95E-04	1.09	2.22	3.21
Murukku (Indian savoury crackers)<br>Potato chips<br>Chicken-flavoured snack<br>Seafood-flavoured snack<br>Fruit/vegetable-flavoured snack	4.57E-04	59.44	0.79	5.31E-03	14.33	11.85	17.16	2.13E-03	276.56	3.81	2.56E-02	28.03	57.19	82.83
**LOCAL *KUIH-MUIH***	**2.40E-04**	**31.19**	**0.41**	**2.79E-03**	**7.52**	**6.22**	**9.01**	**9.59E-04**	**124.64**	**1.66**	**1.11E-02**	**30.04**	**24.85**	**35.99**
Kuih denderam<br>Prawn fritter<br>Curry puff<br>*Cakoi*<br>*Vadai*<br>Banana fritters<br>Fried spring rolls<br>Fried *cempedak*<br>Fried sweet potato<br>Fried banana balls	2.40E-04	31.19	0.41	2.79E-03	7.52	6.22	9.01	9.59E-04	124.64	1.72	1.16E-02	12.63	25.77	37.33
**COOKED FOOD (FRIED)**	**2.32E-03**	**301.84**	**4.01**	**2.70E-02**	**72.75**	**60.18**	**87.16**	**7.14E-03**	**928.48**	**12.35**	**8.29E-02**	**223.79**	**185.10**	**268.11**
Fried rice	1.27E-03	164.68	2.19	1.47E-02	16.09	32.83	47.55	3.32E-03	431.12	5.95	4.00E-02	43.70	89.15	129.13
Char-kuey-teow, fried rice noodle	3.01E-04	39.12	0.52	3.50E-03	9.43	7.80	11.30	9.74E-04	126.59	1.75	1.17E-02	12.83	26.18	37.92
Fried wheat noodle	2.97E-04	38.63	0.51	3.45E-03	3.77	7.70	11.15	1.13E-03	147.30	2.03	1.37E-02	14.93	30.46	44.12
Fried Indian Mackerel	2.16E-04	28.03	0.37	2.50E-03	2.74	5.59	8.10	5.90E-04	76.64	1.06	7.10E-03	7.77	15.85	22.96
Indian flatbread (*Roti canai*) , beef martabak	4.20E-05	5.45	0.07	4.87E-04	1.31	1.09	1.58	1.57E-04	20.46	0.28	1.90E-03	2.07	4.23	6.13
Fried anchovies	6.85E-05	8.91	0.12	7.96E-04	0.87	1.78	2.57	3.26E-04	42.42	0.59	3.93E-03	4.30	8.77	12.70
Butter prawn	3.86E-05	5.02	0.07	4.49E-04	0.49	1.00	1.45	1.65E-04	39.70	0.55	3.68E-03	4.02	8.21	11.89
Fried beef<br>*Bergedil daging*<br>Beef *biryani*<br>Beef satay	3.79E-05	4.93	0.07	4.40E-04	1.19	0.98	1.42	1.21E-04	39.70	0.55	3.68E-03	4.02	8.21	11.89
Fried chicken	2.10E-05	2.74	0.04	2.44E-04	0.27	0.55	0.79	7.74E-05	39.70	0.55	3.68E-03	4.02	8.21	11.89
Fried macaroni	1.97E-05	2.56	0.03	2.28E-04	0.25	0.51	0.74	8.00E-05	39.70	0.55	3.68E-03	4.02	8.21	11.89
Fish ball	1.36E-05	1.77	0.02	1.58E-04	0.17	0.35	0.51	6.56E-05	39.70	0.55	3.68E-03	4.02	8.21	11.89
**FAST FOOD**	**6.02E-05**	**7.82**	**0.10**	**6.99E-04**	**1.89**	**1.56**	**2.26**	**2.51E-04**	**32.63**	**0.43**	**2.92E-03**	**7.86**	**6.50**	**9.42**
Beef burger patty	3.70E-05	4.81	0.06	4.29E-04	0.47	0.96	1.39	1.12E-04	14.59	0.20	1.35E-03	1.48	3.02	4.37
Potato fries	1.09E-05	1.42	0.02	1.27E-04	0.14	0.28	0.41	5.11E-05	6.65	0.09	6.16E-04	0.67	1.37	1.99
Frankfurter/sausage	4.97E-06	0.65	0.01	5.77E-05	0.16	0.13	0.19	3.78E-05	4.91	0.07	4.55E-04	0.50	1.01	1.47
Nugget	6.26E-06	0.81	0.01	7.28E-05	0.08	0.16	0.24	2.72E-05	3.54	0.05	3.28E-04	0.36	0.73	1.06
Pizza with beef and onion	1.03E-06	0.13	0.00	1.20E-05	0.01	0.03	0.04	4.51E-06	0.59	0.01	5.43E-05	0.06	0.12	0.18
**COOKED FOOD (STEWED/BOILED)**	**4.38E-04**	**56.94**	**0.76**	**5.09E-03**	**13.72**	**11.35**	**16.44**	**1.86E-03**	**241.24**	**3.21**	**2.16E-02**	**58.14**	**48.09**	**69.66**
Instant noodles	3.74E-04	48.60	0.65	4.34E-03	4.75	9.69	14.03	1.43E-03	185.32	2.56	1.72E-02	18.78	38.32	55.51
Beef in soy sauce, beef curry	3.17E-05	4.13	0.05	3.69E-04	0.99	0.82	1.19	1.02E-04	13.20	0.18	1.22E-03	1.34	2.73	3.95
Chicken *soto*	1.63E-04	21.25	0.28	1.90E-03	2.08	0.34	0.49	4.28E-04	55.63	0.77	5.15E-03	5.64	2.05	2.97
Satay sauce	1.93E-05	2.51	0.03	2.25E-04	0.25	0.50	0.73	7.37E-05	9.58	0.13	8.88E-04	0.97	1.98	2.87
**TOTAL (category)**		**515.24**	**6.85**		**124.19**				**1868.81**	**24.85**		**450.43**		

MALE ADULTS (GE)

FOOD ITEM	Mean consumer LADD (mg/kg/day)	Mean consumer LCR (/100,000 cases)	Mean consumer ACR (cases/year/100,000)	Mean consumer Glycidol fraction related cancer cases	DALY (/100,000 cases)	DALY UI (2.5%)	DALY UI (97.5%)	High consumer LADD (p95) (mg/kg/day)	High consumer LCR (/100,000 cases) p95	High consumer ACR (cases/year/100,000)	High consumer Glycidol fraction related cancer cases	DALY (/100,000 cases)	DALY UI (2.5%)	DALY UI (97.5%)
**VEGETABLE FATS AND OILS**	**1.36E-04**	**17.63**	**0.23**	**1.58E-03**	**4.25**	**3.52**	**5.09**	**6.16E-04**	**80.02**	**1.10**	**1.53E-02**	**41.22**	**34.10**	**49.39**
Margarine, Shortening	6.94E-05	9.02	0.12	1.72E-03	4.62	3.69	5.84	4.71E-04	61.21	0.84	1.17E-02	12.78	25.07	39.68
Mayonnaise	3.31E-07	0.04	0.00	8.21E-06	0.01	0.02	0.03	1.20E-06	0.16	0.00	2.97E-05	0.03	0.06	0.10
Evaporated creamer	3.34E-06	0.43	0.01	8.28E-05	0.09	0.18	0.28	2.12E-05	2.76	0.04	5.27E-04	0.58	1.13	1.79
Concentrated Creamer	6.26E-05	8.14	0.11	1.55E-03	1.70	3.33	5.28	2.67E-04	34.73	0.48	6.63E-03	7.26	14.23	22.52
**MILK AND DAIRY**	**1.39E-05**	**1.81**	**0.02**	**1.61E-04**	**0.44**	**0.36**	**0.52**	**5.38E-06**	**0.70**	**0.01**	**1.33E-04**	**0.36**	**0.29**	**0.45**
Evaporated Milk	3.54E-06	0.46	0.01	8.78E-05	0.10	0.19	0.30	2.25E-05	2.93	0.04	5.59E-04	0.61	1.20	1.90
Butter	1.03E-05	1.35	0.02	2.57E-04	0.28	0.55	0.87	5.03E-05	6.54	0.09	1.25E-03	1.37	2.68	4.24
**CONFECTIONARY**	**2.86E-04**	**37.21**	**0.49**	**3.32E-03**	**8.97**	**7.42**	**10.74**	**1.21E-03**	**157.06**	**2.15**	**3.00E-02**	**80.92**	**64.32**	**101.82**
Flavoured biscuit	6.11E-05	7.95	0.11	1.52E-03	1.66	3.26	5.15	2.71E-04	35.17	0.48	6.72E-03	7.35	14.40	22.80
Plain biscuit	1.80E-04	23.40	0.32	4.47E-03	4.89	9.58	15.17	7.54E-04	97.99	1.34	1.87E-02	20.47	40.13	63.52
Bun	3.16E-05	4.11	0.06	7.86E-04	0.86	1.68	2.67	3.18E-05	4.14	0.06	7.90E-04	0.86	1.69	2.68
Cake	1.02E-05	1.33	0.02	2.53E-04	0.28	0.54	0.86	3.94E-05	5.12	0.07	9.78E-04	1.07	2.10	3.32
Cheese tart, doughnut	0.00E+00	0.00	0.00	0.00E+00	0.00	0.00	0.00	0.00E+00	0.00	0.00	0.00E+00	0.00	0.00	0.00
Chocolate bar	3.20E-06	0.42	0.01	7.96E-05	0.09	0.17	0.27	1.54E-05	2.00	0.03	3.82E-04	0.42	0.82	1.30
Chocolate spread	0.00E+00	0.00	0.00	0.00E+00	0.00	0.00	0.00	0.00E+00	0.00	0.00	0.00E+00	0.00	0.00	0.00
**SNACKS**	**4.40E-04**	**57.14**	**0.76**	**5.10E-03**	**13.77**	**11.39**	**16.50**	**2.03E-04**	**26.42**	**0.36**	**5.04E-03**	**13.61**	**10.82**	**17.13**
Fried fish sausage (*Keropok lekor*)	2.03E-06	0.26	0.00	5.04E-05	0.06	0.11	0.17	8.56E-06	1.11	0.02	2.12E-04	0.23	0.46	0.72
Fried Fish Crackers (*Keropok Ikan*)	1.83E-05	2.38	0.03	4.55E-04	0.50	0.98	1.54	9.39E-05	12.21	0.17	2.33E-03	2.55	5.00	7.92
Murukku (Indian savoury crackers)<br>Potato chips<br>Chicken-flavoured snack<br>Seafood-flavoured snack<br>Fruit/vegetable-flavoured snack	4.19E-04	54.49	0.75	1.04E-02	27.94	22.32	35.33	2.04E-03	265.42	3.64	5.07E-02	55.44	108.70	172.07
**LOCAL *KUIH-MUIH***	**2.60E-04**	**33.86**	**0.45**	**3.03E-03**	**8.16**	**6.75**	**9.78**	**9.70E-04**	**126.04**	**1.73**	**2.41E-02**	**64.93**	**51.62**	**81.71**
Kuih denderam<br>Prawn fritter<br>Curry puff<br>*Cakoi*<br>*Vadai*<br>Banana fritters<br>Fried spring rolls<br>Fried *cempedak*<br>Fried sweet potato<br>Fried banana balls	1.45E-04	18.89	0.26	3.61E-03	3.95	13.87	21.95	9.70E-04	126.04	1.73	2.41E-02	26.33	51.62	81.71
**COOKED FOOD (FRIED)**	**2.70E-03**	**351.36**	**4.67**	**3.14E-02**	**84.69**	**70.05**	**101.46**	**8.28E-03**	**1076.02**	**14.74**	**2.05E-01**	**554.36**	**440.67**	**697.58**
Fried rice	1.51E-03	196.62	2.69	3.75E-02	41.07	80.52	127.47	3.35E-03	435.94	5.97	8.32E-02	91.06	178.53	282.62
Char-kuey-teow, fried rice noodle	3.21E-04	41.73	0.57	7.97E-03	21.39	17.09	27.05	1.31E-03	170.68	2.34	3.26E-02	35.65	69.90	110.65
Fried wheat noodle	3.66E-04	47.60	0.65	9.09E-03	9.94	19.49	30.86	1.34E-03	174.38	2.39	3.33E-02	36.42	71.41	113.05
Fried Indian Mackerel	2.21E-04	28.79	0.39	5.50E-03	6.01	11.79	18.66	6.37E-04	82.75	1.13	1.58E-02	17.28	33.89	53.65
Indian flatbread (*Roti canai*), beef martabak	6.07E-05	7.89	0.11	1.51E-03	4.04	3.23	5.11	1.99E-04	25.86	0.35	4.94E-03	5.40	10.59	16.76
Fried anchovies	7.08E-05	9.20	0.13	1.76E-03	1.92	3.77	5.96	3.29E-04	42.75	0.59	8.16E-03	8.93	17.51	27.71
Butter prawn	4.00E-05	5.21	0.07	9.94E-04	1.09	2.13	3.37	1.67E-04	21.68	0.30	4.14E-03	4.53	8.88	14.06
Fried beef<br>*Bergedil daging*<br>Beef *biryani*<br>Beef satay	5.07E-05	6.59	0.09	1.26E-03	3.38	2.70	4.27	1.84E-04	23.90	0.33	4.56E-03	4.99	9.79	15.50
Fried chicken	2.67E-05	3.47	0.05	6.63E-04	0.72	1.42	2.25	7.82E-05	10.17	0.14	1.94E-03	2.12	4.17	6.59
Fried macaroni	1.81E-05	2.35	0.03	4.49E-04	0.49	0.96	1.52	7.47E-05	9.71	0.13	1.85E-03	2.03	3.97	6.29
Fish ball	1.48E-05	1.92	0.03	3.67E-04	0.40	0.79	1.24	6.63E-05	8.62	0.12	1.65E-03	1.80	3.53	5.59
**FAST FOOD**	**7.46E-05**	**9.70**	**0.13**	**8.66E-04**	**2.34**	**1.93**	**2.80**	**3.12E-04**	**40.60**	**0.56**	**7.75E-03**	**20.92**	**16.63**	**26.32**
Beef burger patty	4.88E-05	6.34	0.09	1.21E-03	1.32	2.60	4.11	1.31E-04	17.02	0.23	3.25E-03	3.56	6.97	11.03
Potato fries	1.21E-05	1.57	0.02	3.01E-04	0.33	0.64	1.02	5.17E-05	6.72	0.09	1.28E-03	1.40	2.75	4.36
Frankfurter/sausage	5.50E-06	0.71	0.01	1.36E-04	0.37	0.29	0.46	3.31E-05	4.30	0.06	8.21E-04	0.90	1.76	2.79
Nugget	7.03E-06	0.91	0.01	1.75E-04	0.19	0.37	0.59	3.31E-05	4.30	0.06	8.21E-04	0.90	1.76	2.79
Pizza with beef and onion	1.18E-06	0.15	0.00	2.92E-05	0.03	0.06	0.10	4.56E-06	0.59	0.01	1.13E-04	0.12	0.24	0.38
**COOKED FOOD (STEWED/BOILED)**	**7.18E-04**	**93.33**	**1.24**	**8.34E-03**	**22.49**	**18.61**	**26.95**	**1.83E-03**	**237.26**	**3.25**	**4.53E-02**	**122.24**	**97.17**	**153.82**
Instant noodles	4.61E-04	59.88	0.82	1.14E-02	12.51	24.52	38.82	1.69E-03	219.38	3.01	4.19E-02	45.82	89.84	142.22
Beef in soy sauce, beef curry	4.25E-05	5.52	0.08	1.05E-03	2.83	2.26	3.58	1.54E-04	20.02	0.27	3.82E-03	4.18	8.20	12.98
Chicken *soto*	1.95E-04	25.37	0.35	4.84E-03	5.30	10.39	16.45	4.33E-04	56.25	0.77	1.07E-02	11.75	23.04	36.47
Satay sauce	1.97E-05	2.56	0.04	4.88E-04	0.53	1.05	1.66	9.55E-05	12.41	0.17	2.37E-03	2.59	5.08	8.05
**TOTAL (category)**		**602.03**	**8.01**		**145.10**				**1744.11**	**23.89**		**898.56**		

FEMALE ADULTS (GE)

FOOD ITEM	Mean consumer LADD (mg/kg/day)	Mean consumer LCR (/100,000 cases)	Mean consumer ACR (cases/year/100,000)	Mean consumer Glycidol fraction related cancer cases	DALY (/100,000 cases)	DALY UI (2.5%)	DALY UI (97.5%)	High consumer LADD (p95) (mg/kg/day)	High consumer LCR (/100,000 cases) p95	High consumer ACR (cases/year/100,000)	High consumer Glycidol fraction related cancer cases	DALY (/100,000 cases)	DALY UI (2.5%)	DALY UI (97.5%)
**VEGETABLE FATS AND OILS**	**1.17E-04**	**15.16**	**0.19**	**2.53E-03**	**6.92**	**5.43**	**8.61**	**4.91E-04**	**63.77**	**0.82**	**1.06E-02**	**28.68**	**22.86**	**36.24**
Margarine, Shortening	2.74E-07	0.04	0.00	5.93E-06	0.02	0.01	0.02	4.02E-04	52.29	0.67	8.72E-03	9.53	18.74	29.71
Mayonnaise	2.74E-07	0.04	0.00	5.93E-06	0.01	0.01	0.02	1.36E-06	0.18	0.00	1.64E-05	0.02	0.06	0.10
Evaporated creamer	2.42E-06	0.31	0.00	5.24E-05	0.06	0.11	0.18	1.38E-05	1.79	0.02	1.66E-04	0.18	0.64	1.02
Concentrated Creamer	4.43E-05	5.76	0.07	9.60E-04	1.05	2.07	3.27	1.73E-04	22.53	0.31	2.09E-03	2.28	8.07	12.80
**MILK AND DAIRY**	**1.17E-05**	**1.53**	**0.02**	**2.54E-04**	**0.70**	**0.55**	**0.87**	**3.60E-06**	**0.47**	**0.01**	**7.79E-05**	**0.21**	**0.17**	**0.27**
Evaporated Milk	2.57E-06	0.33	0.00	5.56E-05	0.06	0.12	0.19	1.46E-05	1.90	0.03	1.76E-04	0.19	0.68	1.08
Butter	9.27E-06	1.21	0.02	2.01E-04	0.22	0.43	0.68	5.03E-05	6.54	0.09	6.06E-04	0.66	2.34	3.72
**CONFECTIONARY**	**3.48E-04**	**45.28**	**0.58**	**7.55E-03**	**20.66**	**16.23**	**25.73**	**1.78E-03**	**231.26**	**2.97**	**3.85E-02**	**104.00**	**82.88**	**131.42**
Flavoured biscuit	5.36E-05	6.97	0.09	1.16E-03	1.27	2.50	3.96	3.70E-04	48.08	0.66	4.46E-03	4.87	17.23	27.32
Plain biscuit	2.61E-04	33.97	0.44	5.66E-03	6.19	12.17	19.30	1.00E-03	130.65	1.80	1.21E-02	13.24	46.82	74.24
Bun	1.86E-05	2.42	0.03	4.04E-04	0.44	0.87	1.38	8.38E-05	10.89	0.15	1.01E-03	1.10	3.90	6.19
Cake	1.16E-05	1.51	0.02	2.52E-04	0.28	0.54	0.86	6.48E-05	8.42	0.12	7.81E-04	0.85	3.02	4.79
Cheese tart, doughnut	1.98E-05	2.57	0.03	4.28E-04	1.17	0.92	1.46	1.30E-05	1.69	0.02	2.82E-04	0.31	0.61	0.96
Chocolate bar	3.83E-06	0.50	0.01	8.31E-05	0.09	0.18	0.28	1.75E-05	2.28	0.03	2.11E-04	0.23	0.82	1.30
Chocolate spread	1.58E-06	0.20	0.00	3.42E-05	0.04	0.07	0.12	1.86E-06	0.24	0.00	2.24E-05	0.02	0.09	0.14
**SNACKS**	**6.01E-04**	**78.08**	**1.00**	**1.30E-02**	**35.63**	**27.98**	**44.37**	**3.10E-03**	**402.81**	**5.18**	**6.71E-02**	**181.14**	**144.37**	**228.90**
Fried fish sausage (*Keropok lekor*)	1.46E-06	0.19	0.00	3.17E-05	0.03	0.07	0.11	5.85E-06	0.76	0.01	7.04E-05	0.08	0.27	0.43
Fried Fish Crackers (*Keropok Ikan*)	1.85E-05	2.40	0.03	4.00E-04	0.44	0.86	1.36	8.91E-05	11.59	0.16	1.07E-03	1.17	4.15	6.59
Murukku (Indian savoury crackers)<br>Potato chips<br>Chicken-flavoured snack<br>Seafood-flavoured snack<br>Fruit/vegetable-flavoured snack	5.81E-04	75.49	0.97	0.01	34.45	27.05	42.90	3.05E-03	396.01	5.09	0.07	72.20	141.93	225.04
**LOCAL *KUIH-MUIH***	**2.54E-04**	**33.03**	**0.42**	**5.51E-03**	**15.07**	**11.84**	**18.77**	**1.10E-03**	**143.55**	**1.85**	**2.39E-02**	**64.55**	**51.45**	**81.57**
Kuih denderam<br>Prawn fritter<br>Curry puff<br>*Cakoi*<br>*Vadai*<br>Banana fritters<br>Fried spring rolls<br>Fried *cempedak*<br>Fried sweet potato<br>Fried banana balls	2.54E-04	33.03	0.42	5.51E-03	15.07	11.84	18.77	9.59E-04	124.64	1.60	2.08E-02	22.72	44.67	70.83
**COOKED FOOD (FRIED)**	**2.23E-03**	**290.21**	**3.73**	**4.84E-02**	**132.43**	**104.01**	**164.91**	**6.86E-03**	**891.78**	**11.46**	**1.49E-01**	**401.03**	**319.61**	**506.76**
Fried rice	1.17E-03	152.12	1.96	2.54E-02	27.74	54.52	86.45	2.49E-03	323.34	4.46	3.00E-02	32.77	115.88	183.74
Char-kuey-teow, fried rice noodle	3.26E-04	42.39	0.54	7.07E-03	19.3	15.2	24.1	8.66E-04	112.53	1.45	1.88E-02	20.52	40.33	63.95
Fried wheat noodle	2.61E-04	33.94	0.44	5.66E-03	6.19	12.16	19.29	8.50E-04	110.48	1.52	1.02E-02	11.20	39.60	62.78
Fried Indian Mackerel	2.44E-04	31.74	0.41	5.29E-03	5.79	11.37	18.03	5.90E-04	76.64	1.06	7.10E-03	7.77	27.47	43.55
Indian flatbread (*Roti canai*) , beef martabak	2.55E-05	3.32	0.04	5.53E-04	1.5	1.2	1.9	7.87E-05	10.23	0.13	1.71E-03	1.87	3.67	5.81
Fried anchovies	7.70E-05	10.01	0.13	1.67E-03	1.83	3.59	5.69	3.26E-04	42.42	0.59	3.93E-03	4.30	15.20	24.10
Butter prawn	4.32E-05	5.61	0.07	9.35E-04	1.02	2.01	3.19	1.55E-04	39.70	0.55	3.68E-03	4.02	14.23	22.56
Fried beef<br>*Bergedil daging*<br>Beef *biryani*<br>Beef satay	8.59E+04	3.66	0.05	4.71E-02	1.70	1.67	1.31	9.09E-05	39.70	0.51	6.62E-03	7.24	14.23	22.56
Fried chicken	1.76E-05	2.28	0.03	3.80E-04	0.42	0.82	1.30	7.74E-05	39.70	0.55	3.68E-03	4.02	14.23	22.56
Fried macaroni	2.49E-05	3.24	0.04	5.40E-04	0.59	1.16	1.84	1.11E-04	39.70	0.55	3.68E-03	4.02	14.23	22.56
Fish ball	1.45E-05	1.88	0.02	3.14E-04	0.34	0.67	1.07	5.90E-05	39.70	0.55	3.68E-03	4.02	14.23	22.56
**FAST FOOD**	**5.23E-05**	**6.80**	**0.09**	**1.13E-03**	**3.11**	**2.44**	**3.87**	**2.90E-04**	**37.69**	**0.48**	**6.28E-03**	**16.95**	**13.51**	**21.42**
Beef burger patty	2.86E-05	3.72	0.05	6.20E-04	0.68	1.33	2.11	1.12E-04	14.59	0.20	1.35E-03	1.48	5.23	8.29
Potato fries	1.12E-05	1.46	0.02	2.44E-04	0.27	0.52	0.83	4.72E-05	6.14	0.08	5.69E-04	0.62	2.20	3.49
Frankfurter/sausage	5.09E-06	0.66	0.01	1.10E-04	0.30	0.24	0.38	2.52E-05	3.27	0.04	5.45E-04	0.60	1.17	1.86
Nugget	6.37E-06	0.83	0.01	1.38E-04	0.15	0.30	0.47	2.45E-05	3.19	0.04	2.95E-04	0.32	1.14	1.81
Pizza with beef and onion	1.02E-06	0.13	0.00	2.21E-05	0.02	0.05	0.08	4.51E-06	0.59	0.01	5.43E-05	0.06	0.21	0.33
**COOKED FOOD (STEWED/BOILED)**	**5.25E-04**	**68.27**	**0.88**	**1.14E-02**	**31.15**	**24.47**	**38.79**	**1.43E-03**	**185.37**	**2.38**	**3.09E-02**	**83.36**	**66.44**	**105.34**
Instant noodles	3.28E-04	42.70	0.55	7.12E-03	7.79	15.30	24.27	1.07E-03	138.99	1.92	1.29E-02	14.09	49.81	78.98
Beef in soy sauce, beef curry	2.36E-05	3.07	0.04	5.12E-04	1.40	1.10	1.74	7.61E-05	9.90	0.13	1.65E-03	1.80	3.55	5.62
Chicken *soto*	1.51E-04	19.63	0.25	3.27E-03	3.58	7.03	11.15	3.21E-04	41.72	0.58	3.87E-03	4.23	14.95	23.71
Satay sauce	2.21E-05	2.87	0.04	4.78E-04	0.52	1.03	1.63	7.37E-05	9.58	0.13	8.88E-04	0.97	3.43	5.44
**TOTAL (category)**		**538.35**	**6.92**		**245.67**				**1956.70**	**25.15**		**879.93**		

MALAY (GE)					
**FOOD ITEM**	**LADD (mg/kg/day)**	**Lifetime Cancer Risk (LCR)/100000**	**Mean annual carcinogenic risk (ACR) (cases/year/100,000 cases)**	**Glycidol fraction related cancer cases**	**DALY (per 100,000 cases)**
**VEGETABLE FATS AND OILS***	**1.42E-04**	**18.49**	**0.25**	**1.65E-03**	**4.46**
Margarine, Shortening	7.42E-05	9.65	0.13	8.62E-04	2.33
Mayonnaise	3.68E-07	0.05	0.00	4.28E-06	0.00
Evaporated creamer	3.36E-06	0.44	0.01	3.90E-05	0.04
Concentrated Creamer	6.43E-05	8.36	0.11	7.47E-04	0.82
**MILK AND DAIRY**	**1.08E-05**	**1.40**	**0.02**	**1.25E-04**	**0.34**
Evaporated Milk	3.57E-06	0.46	0.01	4.14E-05	0.05
Butter	7.24E-06	0.94	0.01	8.41E-05	0.09
**CONFECTIONARY**	**3.18E-04**	**41.34**	**0.55**	**3.69E-03**	**9.96**
Flavoured biscuit	6.28E-05	8.17	0.11	7.30E-04	0.80
Plain biscuit	2.16E-04	28.08	0.37	2.51E-03	2.74
Bun	6.94E-06	0.90	0.01	8.06E-05	0.09
Cake	7.64E-06	0.99	0.01	8.88E-05	0.10
Cheese tart, doughnut	2.00E-06	0.26	0.00	2.32E-05	0.06
Chocolate bar	3.66E-06	0.48	0.01	4.26E-05	0.05
Chocolate spread	1.80E-06	0.23	0.00	2.10E-05	0.02
**SNACKS**	**6.11E-04**	**79.45**	**1.06**	**7.10E-03**	**19.15**
Fried fish sausage (*Keropok lekor*)	2.66E-06	0.35	0.00	3.09E-05	0.03
Fried Fish Crackers (*Keropok Ikan*)	2.55E-05	3.31	0.04	2.96E-04	0.32
Murukku (Indian savoury crackers)<br>Potato chips<br>Chicken-flavoured snack<br>Seafood-flavoured snack<br>Fruit/vegetable-flavoured snack	5.83E-04	75.79	1.01	6.77E-03	18.27
**LOCAL *KUIH-MUIH***	**3.01E-04**	**39.11**	**0.52**	**3.49E-03**	**9.43**
Kuih denderam<br>Prawn fritter<br>Curry puff<br>*Cakoi*<br>*Vadai*<br>Banana fritters<br>Fried spring rolls<br>Fried *cempedak*<br>Fried sweet potato<br>Fried banana balls	3.01E-04	39.11	0.52	3.49E-03	9.43
**COOKED FOOD (FRIED)**	**2.20E-03**	**286.44**	**3.81**	**2.56E-02**	**69.04**
Fried rice	1.21E-03	157.84	2.10	1.41E-02	15.42
Char-kuey-teow, fried rice noodle	2.51E-04	32.66	0.43	2.92E-03	7.87
Fried wheat noodle	2.07E-04	26.90	0.36	2.40E-03	2.63
Fried Indian Mackerel	2.48E-04	32.20	0.43	2.88E-03	3.15
ndian flatbread (*Roti canai)* , beef martaba	5.72E-05	7.44	0.10	6.65E-04	1.79
Fried anchovies	7.49E-05	9.73	0.13	8.69E-04	0.95
Butter prawn	4.19E-05	5.45	0.07	4.87E-04	0.53
Fried beef<br>*Bergedil daging*<br>Beef *biryani*<br>Beef satay	7.30E+00	0.10	0.00	1.76E+00	1.46
Fried chicken	2.40E-05	3.12	0.04	2.79E-04	0.30
Fried macaroni	1.89E-05	2.45	0.03	2.19E-04	0.24
Fish ball	1.03E-05	1.34	0.02	1.19E-04	0.13
**FAST FOOD**	**2.36E-05**	**3.07**	**0.04**	**2.74E-04**	**0.74**
Beef burger patty	0.00E+00	0.00	0.00	0.00E+00	0.00
Potato fries	1.13E-05	1.47	0.02	1.31E-04	0.14
Frankfurter/sausage	3.69E-06	0.48	0.01	4.29E-05	0.12
Nugget	7.65E-06	0.99	0.01	8.88E-05	0.10
Pizza with beef and onion	9.81E-07	0.13	0.00	1.14E-05	0.01
**COOKED FOOD (STEWED/BOILED)**	**4.88E-04**	**63.48**	**0.84**	**5.67E-03**	**15.30**
Instant noodles	2.60E-04	33.85	0.45	3.02E-03	3.31
Beef in soy sauce, beef curry	4.71E-05	6.12	0.08	5.47E-04	1.47
Chicken *soto*	1.56E-04	20.34	0.27	1.82E-03	1.99
Satay sauce	2.44E-05	3.17	0.04	2.84E-04	0.31
**TOTAL (category)**		**532.79**	**7.08**		**128.42**

CHINESE (GE)					
**FOOD ITEM**	**LADD (mg/kg/day)**	**Lifetime Cancer Risk (LCR)/100000**	**Mean annual carcinogenic risk (ACR) (cases/year/100,000 cases)**	**Glycidol fraction related cancer cases**	**DALY (per 100,000 cases)**
**VEGETABLE FATS AND OILS**	**6.29E-05**	**8.18**	**0.11**	**7.31E-04**	**1.97**
Margarine, Shortening	6.29E-05	8.18	0.11	7.31E-04	1.97
Mayonnaise	0.00E+00	0.00	0.00	0.00E+00	0.00
Evaporated creamer	0.00E+00	0.00	0.00	0.00E+00	0.00
Concentrated Creamer	0.00E+00	0.00	0.00	0.00E+00	0.00
**MILK AND DAIRY**	**1.30E-05**	**1.69**	**0.02**	**1.51E-04**	**0.41**
Evaporated Milk	0.00E+00	0.00	0.00	0.00E+00	0.00
Butter	1.30E-05	1.69	0.02	1.51E-04	0.16
**CONFECTIONARY**	**1.80E-04**	**23.35**	**0.31**	**2.09E-03**	**5.63**
Flavoured biscuit	2.68E-05	3.48	0.05	3.11E-04	0.34
Plain biscuit	1.17E-04	15.25	0.20	1.36E-03	1.49
Bun	5.08E-06	0.66	0.01	5.90E-05	0.06
Cake	1.21E-05	1.58	0.02	1.41E-04	0.15
Cheese tart, doughnut	2.83E-06	0.37	0.00	3.28E-05	0.09
Chocolate bar	2.94E-06	0.38	0.01	3.42E-05	0.04
Chocolate spread	0.00E+00	0.00	0.00	0.00E+00	0.00
**SNACKS**	**1.71E-04**	**22.29**	**0.30**	**1.99E-03**	**5.37**
Fried fish sausage (*Keropok lekor*)	2.79E-07	0.04	0.00	3.24E-06	0.00
Fried Fish Crackers (*Keropok Ikan*)	0.00E+00	0.00	0.00	0.00E+00	0.00
Murukku (Indian savoury crackers)<br>Potato chips<br>Chicken-flavoured snack<br>Seafood-flavoured snack<br>Fruit/vegetable-flavoured snack	1.71E-04	22.25	0.30	1.99E-03	5.36
**LOCAL *KUIH-MUIH***	**7.14E-05**	**9.28**	**0.12**	**8.29E-04**	**2.24**
Kuih denderam<br>Prawn fritter<br>Curry puff<br>*Cakoi*<br>*Vadai*<br>Banana fritters<br>Fried spring rolls<br>Fried *cempedak*<br>Fried sweet potato<br>Fried banana balls	0.00	9.28	0.12	0.00	2.24
**COOKED FOOD (FRIED)**	**2.22E-03**	**288.75**	**3.84**	**2.58E-02**	**69.60**
Fried rice	1.01E-03	131.80	1.75	1.18E-02	12.88
Char-kuey-teow, fried rice noodle	4.71E-04	61.22	0.81	5.47E-03	14.76
Fried wheat noodle	4.41E-04	57.35	0.76	5.12E-03	5.60
Fried Indian Mackerel	1.21E-04	15.71	0.21	1.40E-03	1.54
ndian flatbread (*Roti canai)* , beef martaba	2.18E-05	2.84	0.04	2.54E-04	0.68
Fried anchovies	2.99E-05	3.89	0.05	3.47E-04	0.38
Butter prawn	4.08E-05	5.30	0.07	4.73E-04	0.52
Fried beef<br>*Bergedil daging*<br>Beef *biryani*<br>Beef satay	1.31E-05	1.70E+00	2.26E-02	1.52E-04	4.09E-01
Fried chicken	1.63E-05	2.12	0.03	1.89E-04	0.21
Fried macaroni	2.93E-05	3.81	0.05	3.40E-04	0.37
Fish ball	2.32E-05	3.01	0.04	2.69E-04	0.29
**FAST FOOD**	**4.36E-05**	**5.67**	**0.08**	**5.06E-04**	**1.37**
Beef burger patty	2.91E-05	3.79	0.05	3.39E-04	0.37
Potato fries	9.35E-06	1.22	0.02	1.09E-04	0.12
Frankfurter/sausage	2.05E-06	0.27	0.00	2.39E-05	0.06
Nugget	2.16E-06	0.28	0.00	2.51E-05	0.03
Pizza with beef and onion	8.62E-07	0.11	0.00	1.00E-05	0.01
**COOKED FOOD (STEWED/BOILED)**	**7.05E-04**	**91.61**	**1.22**	**8.18E-03**	**22.08**
Instant noodles	5.55E-04	72.15	0.96	6.45E-03	7.05
Beef in soy sauce, beef curry	1.09E-05	1.42	0.02	1.27E-04	0.34
Chicken *soto*	1.31E-04	17.01	0.23	1.52E-03	1.66
Satay sauce	7.92E-06	1.03	0.01	9.20E-05	0.10
**TOTAL (category)**		**450.81**	**5.99**		**108.66**

INDIAN (GE)

FOOD ITEM	LADD (mg/kg/day)	Lifetime Cancer Risk (LCR)/100000	Mean annual carcinogenic risk (ACR) (cases/year/100,000 cases)	Glycidol fraction related cancer cases	DALY (per 100,000 cases)
**VEGETABLE FATS AND OILS**	**8.81E-05**	**11.45**	**0.15**	**1.02E-03**	**2.76**
Margarine, Shortening	8.81E-05	11.45	0.15	1.02E-03	2.76
Mayonnaise	0.00E+00	0.00	0.00	0.00E+00	0.00
Evaporated creamer	0.00E+00	0.00	0.00	0.00E+00	0.00
Concentrated Creamer	0.00E+00	0.00	0.00	0.00E+00	0.00
**MILK AND DAIRY**	**0.00E+00**	**0.00**	**0.00**	**0.00E+00**	**0.00**
Evaporated Milk	0.00E+00	0.00	0.00	0.00E+00	0.00
Butter	0.00E+00	0.00	0.00	0.00E+00	0.00
**CONFECTIONARY**	**2.94E-04**	**38.23**	**0.51**	**3.42E-03**	**9.22**
Flavoured biscuit	0.00E+00	0.00	0.00	0.00E+00	0.00
Plain biscuit	2.83E-04	36.78	0.49	3.29E-03	3.59
Bun	0.00E+00	0.00	0.00	0.00E+00	0.00
Cake	1.12E-05	1.45	0.02	1.30E-04	0.14
Cheese tart, doughnut	0.00E+00	0.00	0.00	0.00E+00	0.00
Chocolate bar	0.00E+00	0.00	0.00	0.00E+00	0.00
Chocolate spread	0.00E+00	0.00	0.00	0.00E+00	0.00
**SNACKS**	**2.81E-06**	**0.37**	**0.00**	**3.26E-05**	**0.09**
Fried fish sausage (*Keropok lekor*)	0.00E+00	0.00	0.00	0.00E+00	0.00
Fried Fish Crackers (*Keropok Ikan*)	2.81E-06	0.37	0.00	3.26E-05	0.04
Murukku (Indian savoury crackers)<br>Potato chips<br>Chicken-flavoured snack<br>Seafood-flavoured snack<br>Fruit/vegetable-flavoured snack	0.00E+00	0.00E+00	0.00E+00	0.00E+00	0.00E+00
**LOCAL *KUIH-MUIH***	**2.27E-04**	**29.50**	**0.39**	**2.64E-03**	**7.11**
Kuih denderam<br>Prawn fritter<br>Curry puff<br>*Cakoi*<br>*Vadai*<br>Banana fritters<br>Fried spring rolls<br>Fried *cempedak*<br>Fried sweet potato<br>Fried banana balls	0.00	29.50	0.39	0.00	7.11
**COOKED FOOD (FRIED)**	**1.90E-03**	**247.15**	**3.29**	**2.21E-02**	**59.57**
Fried rice	9.81E-04	127.54	1.70	1.14E-02	12.46
Char-kuey-teow, fried rice noodle	3.34E-04	43.48	0.58	3.88E-03	10.48
Fried wheat noodle	2.69E-04	35.02	0.47	3.13E-03	3.42
Fried Indian Mackerel	1.61E-04	20.95	0.28	1.87E-03	2.05
ndian flatbread (*Roti canai)* , beef martaba	4.16E-05	5.40	0.07	4.83E-04	1.30
Fried anchovies	6.69E-05	8.70	0.12	7.77E-04	0.85
Butter prawn	2.38E-05	3.09	0.04	2.76E-04	0.30
Fried beef<br>*Bergedil daging*<br>Beef *biryani*<br>Beef satay	0.00E+00	0.00	0.00	0.00E+00	0.00
Fried chicken	1.02E-05	1.33	0.02	1.19E-04	0.13
Fried macaroni	0.00E+00	0.00	0.00	0.00E+00	0.00
Fish ball	1.26E-05	1.64	0.02	1.47E-04	0.16
**FAST FOOD**	**3.39E-05**	**4.41**	**0.06**	**3.94E-04**	**1.06**
Beef burger patty	2.12E-05	2.75	0.04	2.46E-04	0.27
Potato fries	7.32E-06	0.95	0.01	8.51E-05	0.09
Frankfurter/sausage	0.00E+00	0.00	0.00	0.00E+00	0.00
Nugget	5.44E-06	0.71	0.01	6.31E-05	0.07
Pizza with beef and onion	0.00E+00	0.00	0.00	0.00E+00	0.00
**COOKED FOOD (STEWED/BOILED)**	**4.66E-04**	**60.52**	**0.80**	**5.41E-03**	**14.59**
Instant noodles	3.39E-04	44.06	0.59	3.94E-03	4.31
Beef in soy sauce, beef curry	0.00E+00	0.00	0.00	0.00E+00	0.00
Chicken *soto*	1.27E-04	16.46	0.22	1.47E-03	1.61
Satay sauce	0.00E+00	0.00	0.00	0.00E+00	0.00
**TOTAL (category)**		**391.62**	**5.21**		**94.39**

OTHERS (minorities) (GE)

FOOD ITEM	LADD (mg/kg/day)	Lifetime Cancer Risk (LCR)/100000	Mean annual carcinogenic risk (ACR) (cases/year/100,000 cases)	Glycidol fraction related cancer cases	DALY (per 100,000 cases)
**VEGETABLE FATS AND OILS**	**5.68E-05**	**7.38**	**0.10**	**6.59E-04**	**1.78**
Margarine, Shortening	0.00E+00	0.00	0.00	0.00E+00	0.00
Mayonnaise	0.00E+00	0.00	0.00	0.00E+00	0.00
Evaporated creamer	0.00E+00	0.00	0.00	0.00E+00	0.00
Concentrated Creamer	5.68E-05	7.38	0.10	6.59E-04	0.72
**MILK AND DAIRY**	**0.00E+00**	**0.00**	**0.00**	**0.00E+00**	**0.00**
Evaporated Milk	0.00E+00	0.00	0.00	0.00E+00	0.00
Butter	0.00E+00	0.00	0.00	0.00E+00	0.00
**CONFECTIONARY**	**2.10E-04**	**27.33**	**0.36**	**2.44E-03**	**6.59**
Flavoured biscuit	4.71E-05	6.13	0.08	5.48E-04	0.60
Plain biscuit	1.63E-04	21.20	0.28	1.89E-03	2.07
Bun	0.00E+00	0.00	0.00	0.00E+00	0.00
Cake	0.00E+00	0.00	0.00	0.00E+00	0.00
Cheese tart, doughnut	0.00E+00	0.00	0.00	0.00E+00	0.00
Chocolate bar	0.00E+00	0.00	0.00	0.00E+00	0.00
Chocolate spread	0.00E+00	0.00	0.00	0.00E+00	0.00
**SNACKS**	**0.00E+00**	**0.00**	**0.00**	**0.00E+00**	**0.00**
Fried fish sausage (*Keropok lekor*)	0.00E+00	0.00	0.00	0.00E+00	0.00
Fried Fish Crackers (*Keropok Ikan*)	0.00E+00	0.00	0.00	0.00E+00	0.00
Murukku (Indian savoury crackers)<br>Potato chips<br>Chicken-flavoured snack<br>Seafood-flavoured snack<br>Fruit/vegetable-flavoured snack	0.00E+00	0.00E+00	0.00E+00	0.00E+00	0.00E+00
**LOCAL *KUIH-MUIH***	**1.68E-04**	**21.88**	**0.29**	**1.95E-03**	**5.27**
Kuih denderam<br>Prawn fritter<br>Curry puff<br>*Cakoi*<br>*Vadai*<br>Banana fritters<br>Fried spring rolls<br>Fried *cempedak*<br>Fried sweet potato<br>Fried banana balls	0.00	21.88	0.29	0.00	5.27
**COOKED FOOD (FRIED)**	**2.44E-03**	**316.64**	**4.21**	**2.83E-02**	**76.32**
Fried rice	1.53E-03	199.05	2.65	1.78E-02	19.45
Char-kuey-teow, fried rice noodle	2.21E-04	28.74	0.38	2.57E-03	6.93
Fried wheat noodle	2.98E-04	38.79	0.52	3.47E-03	3.79
Fried Indian Mackerel	2.13E-04	27.71	0.37	2.48E-03	2.71
ndian flatbread (*Roti canai)* , beef martaba	2.80E-05	3.64	0.05	3.25E-04	0.88
Fried anchovies	5.46E-05	7.09	0.09	6.34E-04	0.69
Butter prawn	2.88E-05	3.74	0.05	3.34E-04	0.37
Fried beef<br>*Bergedil daging*<br>Beef *biryani*<br>Beef satay	2.72E-05	3.53	0.05	3.16E-04	0.85
Fried chicken	2.16E-05	2.81	0.04	2.51E-04	0.27
Fried macaroni	0.00E+00	0.00	0.00	0.00E+00	0.00
Fish ball	1.17E-05	1.53	0.02	1.36E-04	0.15
**FAST FOOD**	**6.63E-06**	**0.86**	**0.01**	**7.70E-05**	**0.21**
Beef burger patty	0.00E+00	0.00	0.00	0.00E+00	0.00
Potato fries	0.00E+00	0.00	0.00	0.00E+00	0.00
Frankfurter/sausage	6.63E-06	0.86	0.01	7.70E-05	0.21
Nugget	0.00E+00	0.00	0.00	0.00E+00	0.00
Pizza with beef and onion	0.00E+00	0.00	0.00	0.00E+00	0.00
**COOKED FOOD (STEWED/BOILED)**	**6.17E-04**	**80.22**	**1.07**	**7.17E-03**	**19.34**
Instant noodles	3.75E-04	48.81	0.65	4.36E-03	4.77
Beef in soy sauce, beef curry	2.28E-05	2.96	0.04	2.64E-04	0.71
Chicken *soto*	1.98E-04	25.68	0.34	2.29E-03	2.51
Satay sauce	2.13E-05	2.77	0.04	2.48E-04	0.27
**TOTAL (category)**		**454.32**	**6.04**		**109.50**

OTHERS (bumiputera) (GE)

FOOD ITEM	LADD (mg/kg/day)	Lifetime Cancer Risk (LCR)/100000	Mean annual carcinogenic risk (ACR) (cases/year/100,000 cases)	Glycidol fraction related cancer cases	DALY (per 100,000 cases)
**VEGETABLE FATS AND OILS**	**3.28E-05**	**4.26**	**0.06**	**3.81E-04**	**1.03**
Margarine, Shortening	0.00E+00	0.00	0.00	0.00E+00	0.00
Mayonnaise	8.91E-08	0.01	0.00	1.04E-06	0.00
Evaporated creamer	2.61E-06	0.34	0.00	3.03E-05	0.08
Concentrated Creamer	3.01E-05	3.91	0.05	3.49E-04	0.94
**MILK AND DAIRY**	**8.13E-06**	**1.06**	**0.01**	**9.44E-05**	**0.25**
Evaporated Milk	2.76E-06	0.36	0.00	3.21E-05	0.09
Butter	5.37E-06	0.70	0.01	6.23E-05	0.17
**CONFECTIONARY**	**3.19E-04**	**41.41**	**0.55**	**3.70E-03**	**9.98**
Flavoured biscuit	5.01E-05	6.52	0.09	5.82E-04	1.57
Plain biscuit	2.40E-04	31.16	0.41	2.78E-03	7.51
Bun	8.28E-06	1.08	0.01	9.62E-05	0.26
Cake	0.00E+00	0.00	0.00	0.00E+00	0.00
Cheese tart, doughnut	0.00E+00	0.00	0.00	0.00E+00	0.00
Chocolate bar	0.00E+00	0.00	0.00	0.00E+00	0.00
Chocolate spread	0.00E+00	0.00	0.00	0.00E+00	0.00
**SNACKS**	**5.38E-04**	**69.94**	**0.93**	**6.25E-03**	**16.86**
Fried fish sausage (*Keropok lekor*)	8.14E-07	0.11	0.00	9.46E-06	0.03
Fried Fish Crackers (*Keropok Ikan*)	1.02E-05	1.32	0.02	1.18E-04	0.32
Murukku (Indian savoury crackers)<br>Potato chips<br>Chicken-flavoured snack<br>Seafood-flavoured snack<br>Fruit/vegetable-flavoured snack	5.27E-04	68.51	0.91	6.12E-03	16.51
**LOCAL *KUIH-MUIH***	**2.78E-04**	**36.15**	**0.48**	**3.23E-03**	**8.71**
Kuih denderam<br>Prawn fritter<br>Curry puff<br>*Cakoi*<br>*Vadai*<br>Banana fritters<br>Fried spring rolls<br>Fried *cempedak*<br>Fried sweet potato<br>Fried banana balls	2.78E-04	36.15	0.48	3.23E-03	8.71
**COOKED FOOD (FRIED)**	**3.01E-03**	**391.04**	**5.20**	**3.49E-02**	**94.25**
Fried rice	1.86E-03	242.43	3.22	2.17E-02	58.43
Char-kuey-teow, fried rice noodle	2.73E-04	35.50	0.47	3.17E-03	8.56
Fried wheat noodle	4.64E-04	60.33	0.80	5.39E-03	14.54
Fried Indian Mackerel	2.52E-04	32.78	0.44	2.93E-03	7.90
ndian flatbread (*Roti canai)* , beef martaba	1.71E-05	2.22	0.03	1.98E-04	0.54
Fried anchovies	0.00E+00	0.00	0.00	0.00E+00	0.00
Butter prawn	3.42E-05	4.44	0.06	3.97E-04	1.07
Fried beef<br>*Bergedil daging*<br>Beef *biryani*<br>Beef satay	2.47E-05	3.21E+00	4.27E-02	2.87E-04	7.74E-01
Fried chicken	2.14E-05	2.78	0.04	2.48E-04	0.67
Fried macaroni	0.00E+00	0.00	0.00	0.00E+00	0.00
Fish ball	1.46E-05	1.90	0.03	1.70E-04	0.46
**FAST FOOD**	**5.36E-05**	**6.97**	**0.09**	**6.23E-04**	**1.68**
Beef burger patty	2.34E-05	3.04	0.04	2.72E-04	0.73
Potato fries	8.71E-06	1.13	0.02	1.01E-04	0.27
Frankfurter/sausage	1.50E-05	1.95	0.03	1.74E-04	0.47
Nugget	6.54E-06	0.85	0.01	7.60E-05	0.20
Pizza with beef and onion	0.00E+00	0.00	0.00	0.00E+00	0.00
**COOKED FOOD (STEWED/BOILED)**	**8.65E-04**	**112.43**	**1.50**	**1.00E-02**	**27.10**
Instant noodles	5.84E-04	75.89	1.01	6.78E-03	18.29
Beef in soy sauce, beef curry	2.07E-05	2.69	0.04	2.40E-04	0.65
Chicken *soto*	2.41E-04	31.28	0.42	2.79E-03	7.54
Satay sauce	1.98E-05	2.57	0.03	2.29E-04	0.62
**TOTAL (category)**		**663.25**	**8.82**		**159.86**
